# AI Designed Conformation Locking Peptides Target STING to Restore Diabetic Wound Healing

**DOI:** 10.1002/advs.76849

**Published:** 2026-08-07

**Authors:** Xinyu Li, Haojie Fu, Zhe Wang, Xuanzhou Chen, Ruhong Zhang, Xudong Wang, Louis D. Zhang, Xiang Li, Datao Li

**Affiliations:** ^1^ Department of Plastic and Reconstructive Surgery Shanghai Ninth People's Hospital Shanghai Jiao Tong University School of Medicine Shanghai China; ^2^ Department of Oral and Craniomaxillofacial Surgery Shanghai Ninth People's Hospital Shanghai Jiao Tong University School of Medicine Shanghai China; ^3^ Institute of Bioengineering College of Chemical and Biological Engineering Zhejiang University Hangzhou China; ^4^ School of Electrical and Computer Engineering Georgia Institute of Technology Atlanta Georgia USA; ^5^ The Wallace H. Coulter Department of Biomedical Engineering Georgia Institute of Technology and Emory University Atlanta Georgia USA; ^6^ Power Dream America, Inc. Peachtree Corners Georgia USA; ^7^ School of Pharmacy Second Military Medical University Shanghai China; ^8^ Department of Plastic and Reconstructive Surgery Hainan Western Central Hospital Danzhou Hainan China

**Keywords:** AI‐designed peptide, cGAS‐STING pathway, diabetic wound healing, dual‐responsive hydrogel, immunomodulation

## Abstract

Diabetic foot ulcers are a major complication of diabetes characterized by persistent inflammation and impaired tissue repair, in part driven by aberrant activation of the cGAS‐STING innate immune pathway. Precision immunomodulation in the protease‐rich wound microenvironment remains challenging because therapeutic efficacy requires both localized retention and responsiveness to pathological cues. Here, we developed an integrated AI‐to‐biomaterial strategy for diabetic wound repair by coupling generative AI‐guided peptide discovery with microenvironment‐responsive local delivery. A structure‐guided deep‐learning pipeline integrating RFDiffusion, ProteinMPNN, and AlphaFold2‐multimer identified SCP‐1, a conformation‐locking peptide designed to stabilize the inactive STING dimer. To enable therapeutic translation, SCP‐1 was incorporated into a dual‐responsive hydrogel (Gel‐SCP‐1) that provides in situ gelation and MMP‐9‐triggered release in the wound bed. Gel‐SCP‐1 suppressed STING‐TBK1‐IRF3 signaling, reduced inflammatory and oxidative stress, promoted reparative macrophage polarization, and enhanced angiogenic activity. In a full‐thickness excisional wound model in db/db diabetic mice, Gel‐SCP‐1 significantly accelerated wound closure and improved tissue regeneration, including enhanced re‐epithelialization and collagen remodeling. These findings establish an AI‐to‐biomaterial therapeutic paradigm for precision immunoregenerative therapy in chronic diabetic wounds.

## Graphical abstract

A generative AI pipeline identifies SCP‐1, a conformation‐locking peptide that stabilizes inactive STING. Incorporated into a dual‐responsive hydrogel for localized, MMP‐9‐triggered release, SCP‐1 suppresses inflammation, promotes reparative macrophage polarization, and accelerates diabetic wound healing.

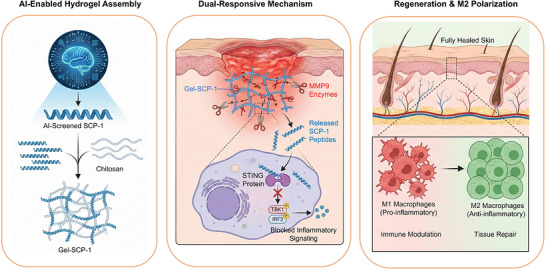

## 1 Introduction

Diabetic foot ulcers (DFUs) are among the most debilitating and costly complications of diabetes mellitus, frequently leading to infection, amputation, and long‐term disability [1]. Unlike acute wounds that resolve through a coordinated inflammatory–proliferative–remodeling cascade, diabetic wounds are typically arrested in a chronic inflammatory state [2]. This non‐resolving inflammation is sustained by a hostile microenvironment characterized by oxidative stress, elevated protease activity (notably matrix metalloproteinases, MMPs), impaired angiogenesis, and defective immune resolution [3]. A hallmark of this failure is the inability of macrophages to transition from a pro‐inflammatory (M1) to a reparative (M2) phenotype, which perpetuates cytokine production, tissue damage, and stalled regeneration [4]. Therefore, therapeutic strategies that reprogram the immune microenvironment and restore inflammatory resolution are critical for breaking the vicious cycle underlying diabetic non‐healing wounds.

Emerging evidence implicates aberrant activation of the cyclic GMP‐AMP synthase (cGAS)‐stimulator of interferon genes (STING) pathway as a central driver of sterile inflammation in metabolic and tissue‐injury settings [5, 6]. Upon sensing cytosolic DNA, STING activates downstream TBK1/IRF3 signaling and amplifies inflammatory cytokine production and reactive oxygen species (ROS) generation [7], thereby reinforcing oxidative damage and sustaining macrophages in a pro‐inflammatory M1‐dominant state. In diabetic wounds, this persistent innate immune activation disrupts the transition from inflammation to repair and ultimately impairs re‐epithelialization, extracellular matrix remodeling, and neovascularization. These features identify STING as an attractive therapeutic target for diabetic wound healing. However, achieving effective STING modulation in this setting remains challenging for two reasons. First, the relevant regulatory surfaces of STING are conformation‐dependent and resemble protein‐protein interaction interfaces, making them difficult to target with conventional small molecules. Second, even when active inhibitors are available, durable efficacy in diabetic wounds is hard to achieve because the wound bed is spatially heterogeneous, protease‐rich, and mechanically dynamic, requiring localized and temporally matched drug exposure. These challenges are even greater for peptide discovery. Conventional screening strategies, such as phage display, mRNA display, and high‐throughput library selection, sample vast but essentially random sequence spaces and identify hits only after experimental enrichment. While effective for canonical binding pockets, these methods are poorly suited for discovering a peptide that selectively stabilizes an inactive STING conformation because they do not explicitly encode target state selectivity, cannot practically explore the enormous combinatorial space of even short peptides, and provide limited structural insight into the resulting binding geometry. By contrast, generative deep‐learning frameworks invert this logic: they begin with a structurally defined target state, construct complementary backbone geometries, and then assign sequences predicted to realize those geometries. In this way, conformation selectivity becomes a design constraint rather than a post hoc screening outcome. For STING, whose activation depends on large‐scale conformational rearrangement, such an AI‐guided strategy offers a uniquely tractable route to discovering conformation‐locking peptide inhibitors that would be difficult to obtain through conventional screening.

Building on this premise, recent advances in geometric deep learning provide a powerful foundation for state‐selective ligand discovery. Diffusion‐based generative models such as RFDiffusion can create *de novo* protein or peptide backbones at near‐atomic resolution by iteratively denoising random coordinates into physically plausible structures [8, 9], whereas inverse‐folding models such as ProteinMPNN can assign amino acid sequences that are likely to adopt the designed backbone while preserving favorable biophysical properties [10]. The resulting peptide‐target complexes can then be evaluated by AlphaFold2‐based structural prediction to further prioritize high‐confidence candidates [11, 12]. Importantly, the value of AI in this context extends beyond candidate generation: it compresses an otherwise prohibitive search space, embeds conformational state selectivity into the design objective, and yields atomistic structural hypotheses that can directly guide downstream validation and mechanistic interpretation. More broadly, AI is increasingly reshaping biomaterial and drug delivery research, including recent efforts to improve formulation design and therapeutic performance through predictive modeling [13]. In chronic diabetic wounds, successful translation of an AI‐designed immunomodulator also requires a delivery platform capable of adapting to the pathological microenvironment. To address this need, we developed an integrated AI‐to‐biomaterial strategy in which a generative AI pipeline was used to identify a conformation‐locking STING‐inhibitory peptide, SCP‐1, and this peptide was then incorporated into a dual‐responsive hydrogel (Gel‐SCP‐1) for localized, on‐demand delivery. The hydrogel, composed of carboxymethyl chitosan crosslinked with an MMP‐9‐cleavable peptide linker and formulated with β‐glycerophosphate, was designed to combine temperature‐triggered in situ gelation with enzyme‐triggered release [14, 15]. Through this coupling of AI‐guided molecular design and disease‐microenvironment‐responsive biomaterial engineering, we sought to achieve spatially confined STING inhibition, attenuate inflammatory and oxidative amplification, promote reparative macrophage polarization, and ultimately restore tissue regeneration in diabetic wounds [16, 17].

**SCHEME 1:**
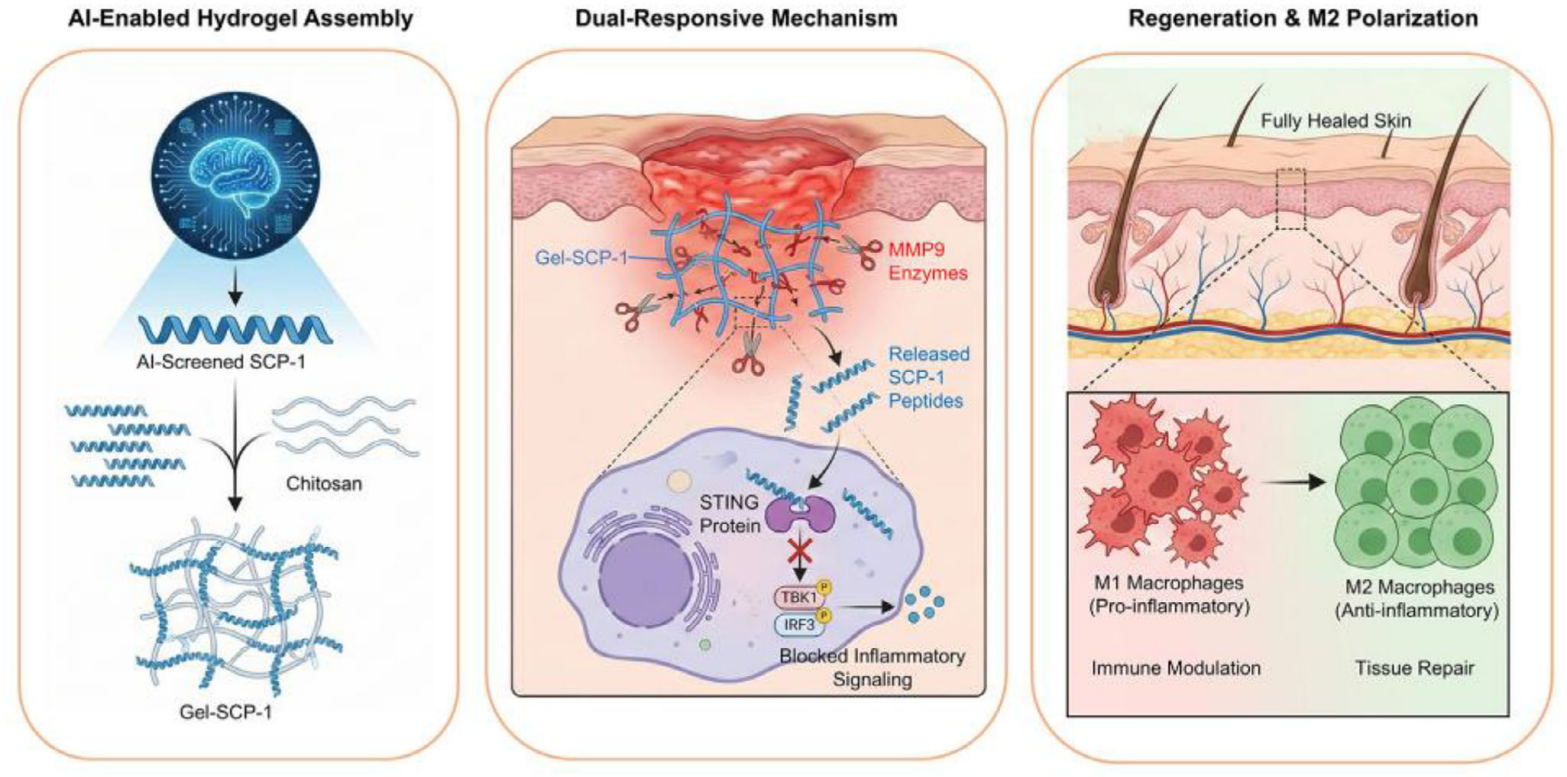
Schematic illustration of the AI‐to‐biomaterial strategy for diabetic wound repair. A generative AI‐guided pipeline identifies SCP‐1 as a conformation‐locking peptide inhibitor of STING and incorporates it into a dual‐responsive hydrogel (Gel‐SCP‐1). Following topical application, the hydrogel undergoes localized retention within the wound bed and responds to elevated MMP‐9 activity by releasing SCP‐1 in a microenvironment‐adaptive manner. The released peptide binds STING and suppresses downstream inflammatory signaling, thereby attenuating pro‐inflammatory activation, promoting macrophage polarization toward the reparative M2 phenotype, and facilitating angiogenesis, tissue regeneration, and wound closure.

In this work, we developed an integrated AI‐to‐biomaterial strategy for precision immunoregenerative therapy in diabetic wounds. A structure‐guided generative AI pipeline was first used to identify SCP‐1, a conformation‐locking peptide designed to inhibit STING, and this peptide was subsequently incorporated into a dual‐responsive hydrogel (Gel‐SCP‐1) for localized and microenvironment‐responsive delivery. We then investigated the material properties and biological performance of this platform across in vitro and in vivo models. Gel‐SCP‐1 exhibited favorable physicochemical characteristics, biocompatibility, and MMP‐responsive release behavior, while effectively suppressing STING‐associated inflammatory signaling, reducing oxidative stress, promoting reparative macrophage polarization, and enhancing angiogenic activity. In diabetic mice, these combined effects translated into accelerated wound closure and improved tissue regeneration, including enhanced re‐epithelialization and extracellular matrix remodeling. Taken together, this work establishes a broadly applicable framework for coupling generative AI‐enabled discovery of conformation‐selective peptide ligands with disease‐adaptive biomaterial delivery and highlights its potential for treating chronic wounds through precise modulation of innate immune dysfunction (Scheme 1).

## 2 Materials and Methods

### 2.1 Materials and Reagents

Chitosan (CS, degree of deacetylation ≥ 95%, viscosity 100–200 mPa s), carboxymethyl chitosan (CMCS), and β‐glycerophosphate disodium salts (β‐GP) were purchased from Macklin Biochemical Co., Ltd. (Shanghai, China). The matrix metalloproteinase‐9 (MMP‐9)‐cleavable peptide linker (GPLGVRGC) was synthesized and purified to >95% (HPLC) by Gill Biochemical Co., Ltd. (Shanghai, China). 1‐Ethyl‐3‐(3‐dimethylaminopropyl) carbodiimide (EDC), N‐hydroxysuccinimide (NHS), and recombinant human MMP‐9 enzyme were obtained from Sigma‐Aldrich (USA). Cell culture reagents, including Dulbecco's modified Eagle medium (DMEM), fetal bovine serum (FBS), and penicillin‐streptomycin, were sourced from Gibco (USA). Primary antibodies against CD31, VEGF, iNOS, CD206, CD86, STING, p‐STING, TBK1, p‐TBK1, IRF3, p‐IRF3, p65, p‐p65, and β‐actin were purchased from Abcam (UK) or Cell Signaling Technology (USA). The covalent STING antagonist H‐151 (HY‐112693) was purchased from MedChemExpress (MCE, USA). Laboratory consumables were provided by Suzhou CellPro Biotechnology Co., Ltd. All other chemicals were of analytical grade and used as received.

### 2.2 Synthesis of MMP‐9 Responsive Chitosan (MMP9‐CS)

To endow enzyme responsiveness, an MMP‐9‐cleavable peptide linker was grafted onto the CMCS backbone via EDC/NHS‐mediated amide coupling [14, 15]. Briefly, CMCS (0.5 g) was dissolved in 50 mL of 0.1 m MES buffer (pH 5.5) under magnetic stirring until fully solubilized. Subsequently, EDC (625 mg) and NHS (345 mg) were added to activate CMCS carboxyl groups, and the mixture was stirred for 30 min in the dark at room temperature. An amine‐terminated MMP‐9‐cleavable peptide (GPLGVRGC, 100 mg) was then added, and the conjugation reaction was allowed to proceed for 24 h at room temperature under gentle stirring. The reaction solution was transferred into dialysis tubing (MWCO 12 kDa) and dialyzed against deionized water for 3 days with regular water replacement to remove unreacted reagents and by‐products. The resulting MMP9‐CS conjugate was collected by lyophilization and stored at ‐20°C until use.

### 2.3 Preparation of SCP‐1‐Loaded Dual‐Responsive Hydrogel (Gel‐SCP‐1)

A dual‐responsive injectable hydrogel was prepared via a temperature‐triggered sol‐gel transition [18, 19]. CS was dissolved in 1% (v/v) acetic acid to obtain a 2% (w/v) solution (Solution A). MMP9‐CS was dissolved in phosphate‐buffered saline (PBS) to yield a 2% (w/v) solution (Solution B). β‐GP solution (Solution C, 500 mg mL^−1^) was prepared by dissolving β‐GP in deionized water, and the therapeutic peptide SCP‐1 was dissolved in Solution C to the desired concentration. For hydrogel formation, Solutions A and B were mixed at a 1:1 (v/v) ratio in an ice bath to minimize premature gelation. Solution C was then added dropwise into the mixture at a volumetric ratio of (A + B):C = 6:4 under continuous stirring (500 rpm) for 10 min at 4°C, yielding a homogeneous pre‐gel solution. Gelation occurred within ≈5 min upon incubation at 37°C, forming the SCP‐1‐loaded hydrogel (Gel‐SCP‐1). A blank hydrogel (Blank Gel) was prepared using the same procedure without SCP‐1.

### 2.4 Computational Design and Screening Pipeline

De novo peptide binders targeting STING were designed using a hierarchical generative workflow focused on the ligand‐binding domain (LBD) of the STING dimer in its inactive, V‐shaped conformation. The crystal structure of the inactive STING dimer served as the target for backbone generation utilizing RFDiffusion [8]. We employed this generative model, conditioned on protein structure motifs, to “hallucinate” peptide backbones that precisely complement the STING dimer interface. To bias generation toward productive binding geometries, residues at the STING dimer interface were specified as contig constraints (hotspots) to guide the diffusion trajectory, ensuring the generated backbones occupied the central cleft [8, 10]. Following this, ProteinMPNN was used to determine the optimal amino acid sequence for each generated backbone. By treating the protein structure as a graph and passing messages between neighboring residues, ProteinMPNN assigned sequences with a high probability of folding into the designed backbone, while simultaneously optimizing physicochemical properties such as foldability and solubility. The structural plausibility and binding modes of designed peptide‐STING complexes were assessed with AlphaFold2‐multimer [11]. Candidates were prioritized using a hierarchical filtering strategy based on: i) structural convergence, requiring a backbone root‐mean‐square deviation (RMSD) < 2.5 Å between the designed model and the AlphaFold2‐multimer prediction to ensure self‐consistency; ii) local confidence, retaining peptides with mean predicted local distance difference test (pLDDT) > 80 to enrich for high‐quality local structure; and *iii*) interaction confidence, requiring interface predicted template modeling (ipTM) > 0.8 to select complexes with high‐confidence protein‐peptide interfaces. Top‐ranked designs were further evaluated using FuzzyScore and predicted aligned error (PAE) metrics. Final candidates were selected following manual inspection of predicted interfaces in PyMOL, focusing on hydrogen‐bonding networks, aromatic contacts, and overall shape complementarity within the STING cleft. Using this workflow, SCP‐1 (SAFDQLLLKNFRRLS) was identified as the lead peptide. The predicted complex indicated stable hydrogen bonds with STING residues Arg238, Gln273, and Tyr167, along with π‐π stacking mediated by Phe3 and Phe11, consistent with stabilization of the inactive, V‐shaped dimer conformation.

### 2.5 Surface Plasmon Resonance (SPR) Binding Analysis

The interaction between STING and SCP‐1 was characterized by SPR on a Biacore T200 instrument (GE Healthcare, Uppsala, Sweden) at 25°C [20]. Purified recombinant human STING ligand‐binding domain (LBD; residues 139–379; purity >95% as verified by SDS‐PAGE and size‐exclusion chromatography), corresponding to the inactive V‐shaped dimer conformation targeted in our AI‐guided peptide design, was used for immobilization [21, 22]. STING protein was diluted to 50 µg mL^−1^ in 10 mm sodium acetate buffer (pH 5.0) and covalently immobilized onto flow cell 2 of a Series S CM5 sensor chip using amine‐coupling chemistry, with a target level of 2500 RU [23]. Flow cell 1 was processed identically without protein immobilization and used as the reference channel. Remaining activated groups were quenched with 1 m ethanolamine‐HCl (pH 8.5). Binding experiments were performed in HBS‐EP+ buffer (10 mm HEPES, 150 mm NaCl, 3 mm EDTA, 0.5% [v/v] Surfactant P20, pH 7.4) at 30 µL min^−1^. SCP‐1 was prepared as a 7‐point, 2‐fold serial dilution in running buffer, with concentrations ranging from 0.3125 to 20 µm. Samples, together with two blank injections of running buffer, were injected over the sensor surface for 120 s, followed by a 120 s dissociation phase. After each cycle, the chip surface was regenerated with 10 mm glycine‐HCl (pH 2.5) for 30 s. These regeneration conditions did not result in any appreciable loss of STING activity over 10 consecutive cycles. Sensorgrams were recorded in real time and processed by double‐reference subtraction to correct for nonspecific binding and bulk effects [24, 25]. Kinetic and affinity parameters were obtained by global fitting using the Kinetics model in Biacore T200 Evaluation Software (version 1.0). All measurements were performed in three independent biological replicates [25].

### 2.6 Material Characterization

The chemical structures of the synthesized polymers and hydrogels were characterized by proton nuclear magnetic resonance (^1^H‐NMR, Bruker Avance III 600 MHz) and Fourier‐transform infrared spectroscopy (FTIR, Thermo Scientific). Surface elemental composition and successful peptide conjugation were further confirmed by X‐ray photoelectron spectroscopy (XPS, K‐Alpha, Thermo Scientific) [26]. The microstructure of lyophilized hydrogels was examined using scanning electron microscopy (SEM, FEI Apreo HiVac). Elemental mapping was performed by energy dispersive X‐ray spectroscopy (EDS). Thermogravimetric analysis (TGA) was conducted from 25 to 600°C under nitrogen atmosphere to evaluate thermal stability and bound/free water content [19]. Rheological measurements were performed on a rotational rheometer (DHR‐2, TA Instruments). Temperature‐dependent sol‐gel transition behavior was determined by monitoring the storage modulus (G′) and loss modulus (G″) during a temperature ramp from 4 to 45°C [27, 28]. Strain sweep tests (0.1%–1000%) were performed to determine the LVR and assess mechanical robustness and recovery behavior. Frequency sweeps were conducted from 0.1 to 100 rad s^−1^ to evaluate viscoelasticity.

### 2.7 In Vitro Enzyme‐Responsive Drug Release and Hydrogel Degradation

To evaluate MMP‐9‐responsive release, Gel‐SCP‐1 was incubated in PBS (pH 7.4) containing recombinant human MMP‐9 at 0, 2, 10, or 50 U mL^−1^ at 37°C. At predetermined time points, supernatants were collected and replenished with fresh release medium to maintain sink conditions. The amount of released SCP‐1 was quantified by high‐performance liquid chromatography (HPLC) and a Micro BCA Protein Assay Kit according to the manufacturer's instructions. For macroscopic degradation, dye‐stained hydrogels were incubated in PBS or MMP‐9 solution (50 U mL^−1^) at 37°C. Representative images were captured at indicated time points to record morphological changes [29].

### 2.8 In Vitro Serum Stability and Protease Resistance Assay

The stability of SCP‐1 was evaluated in fresh human serum and in a protease‐containing buffer mimicking the diabetic wound microenvironment. Briefly, SCP‐1 (50 µm) was incubated in fresh human serum or in PBS (pH 7.4) supplemented with recombinant human MMP‐9 (100 ng mL^−1^) at 37°C [30]. At 0, 2, 4, 8, and 24 h, aliquots were withdrawn and immediately mixed with an equal volume of ice‐cold acetonitrile containing 0.1% TFA to terminate the reaction and precipitate proteins. Following centrifugation, the supernatants were subjected to RP‐HPLC analysis on a C18 column with UV detection at 220 nm. The percentage of intact peptide remaining at each time point was calculated by peak area integration relative to the 0 h sample. The half‐life of SCP‐1 was estimated by fitting the data to a one‐phase exponential decay model using GraphPad Prism [31].

### 2.9 Cytocompatibility and Hemocompatibility

Human umbilical vein endothelial cells (HUVECs; Shanghai iCell Bioscience Inc., China; Cat. No. iCell‐h110) and murine macrophages (RAW264.7) were cultured in DMEM supplemented with 10% FBS and 1% penicillin‐streptomycin at 37°C in a humidified atmosphere containing 5% CO_2_. For cytotoxicity evaluation, cells were seeded in 96‐well plates and treated with hydrogel extracts or free SCP‐1 at indicated concentrations. Cell viability was assessed at 24 and 48 h using a Cell Counting Kit‐8 (CCK‐8) assay following the manufacturer's protocol [32]. Live/Dead staining was performed using Calcein‐AM and propidium iodide (PI), and fluorescence images were acquired [33]. Hemocompatibility was evaluated by a hemolysis assay. Fresh rabbit blood was diluted to 2% (v/v) and incubated with hydrogel samples at 37°C for 1 h. PBS and deionized water were used as negative and positive controls, respectively. After centrifugation, the absorbance of the supernatant was measured at 540 nm, and the hemolysis ratio was calculated as [34]:

$$\mathrm{Hemolysis}\;\;\left(\%\right)=\frac{A_{\mathrm{sample}}-A_{\mathrm{negative}}}{A_{\mathrm{positive}}-A_{\mathrm{negative}}}\;\times 100\%$$

### 2.10 In Vitro Immunogenicity Assessment

Human peripheral blood mononuclear cells (PBMCs; Shanghai iCell Bioscience Inc., China; Cat. No. HUM‐iCell‐i010) were cultured in RPMI 1640 medium supplemented with 10% FBS at a density of 1 × 10^6^ cells mL^−1^. Cells were treated with SCP‐1 at concentrations of 1, 10, and 50 µm for 48 h. Lipopolysaccharide (LPS, 100 ng mL^−1^) was included as a positive control, and untreated cells served as the negative control [35]. Following treatment, culture supernatants were collected, and the concentrations of interleukin‐6 (IL‐6) and tumor necrosis factor‐α (TNF‐α) were measured using ELISA kits according to the manufacturers′ protocols [36].

### 2.11 Immunomodulation and Mechanism Studies

RAW264.7 macrophages were stimulated with lipopolysaccharide (LPS, 1 µg mL^−1^) to induce an inflammatory response and were simultaneously treated with Blank Gel, free SCP‐1, or Gel‐SCP‐1. To verify whether the anti‐inflammatory effects were mediated through STING inhibition, H‐151 (MedChemExpress, HY‐112693), a well‐characterized covalent STING inhibitor, was included as a positive control. Cells in the H‐151 group were treated with 1 µm H‐151 together with LPS under the same timing and culture conditions as those used for the other treatment groups. This concentration was selected based on previous reports showing effective inhibition of STING phosphorylation without apparent cytotoxicity. Macrophage polarization, cytokine secretion, intracellular ROS levels, and western blot analysis were subsequently evaluated using identical protocols for all groups.

#### 2.11.1 Macrophage Polarization

M1 markers (iNOS, CD86) and M2 markers (CD206) were evaluated by immunofluorescence staining. Fluorescence intensity and positive cell percentage were quantified.

#### 2.11.2 Cytokine Analysis

Pro‐inflammatory and anti‐inflammatory cytokines (IL‐6, TNF‐α, IL‐1β, and IL‐10) in culture supernatants were quantified using ELISA kits according to the manufacturers′ instructions. The corresponding mRNA levels in cells were analyzed by quantitative real‐time PCR (qRT‐PCR). Relative gene expression was calculated using the 2^−ΔΔCt^ method.

#### 2.11.3 Intracellular ROS Measurement

ROS levels were assessed using the DCFH‐DA probe. After incubation with DCFH‐DA at 37°C in the dark, cells were washed with PBS and imaged by fluorescence microscopy.

#### 2.11.4 Western Blotting

Cells were lysed, and total protein was quantified. Equal amounts of protein were separated by SDS‐PAGE and transferred onto PVDF membranes. After blocking with 5% milk/BSA, membranes were incubated with primary antibodies against STING, phospho‐STING, TBK1, phospho‐TBK1, IRF3, phospho‐IRF3, NF‐κB p65, phospho‐p65, and β‐actin at 4°C overnight, followed by incubation with HRP‐conjugated secondary antibodies at room temperature. Bands were visualized using an ECL substrate and quantified by densitometry [37].

### 2.12 In Vivo Diabetic Wound Healing Model

Specific pathogen‐free (SPF) male db/db mice (6–8 weeks old, 35–45 g) were purchased from GemPharmatech Co., Ltd. (Nanjing, China). Animals were housed in a controlled facility under a 12 h light/12 h dark cycle, with standard environmental conditions maintained at 22 ± 2°C and 50 ± 5% relative humidity. Mice had free access to standard chow and water throughout the study. All animal protocols were reviewed and approved by the Institutional Animal Care and Use Committee (IACUC) of Shanghai Ninth People’s Hospital, Shanghai Jiao Tong University School of Medicine. Animal manipulations were performed in compliance with the approved protocol, ARRIVE 2.0 guidelines, and institutional and national laboratory animal care standards. Adequate measures including anesthesia, perioperative analgesia, routine monitoring, and predefined humane endpoints were applied to minimize animal pain and distress..

Db/db mice, which spontaneously develop type 2 diabetes due to a homozygous mutation in the leptin receptor gene, were used as the diabetic wound model. Blood glucose levels were monitored throughout the experimental period to confirm sustained hyperglycemia in all animals. Non‐fasting blood glucose was measured from tail vein blood using a portable glucometer (Accu‐Chek, Roche Diagnostics) at baseline (day 0, before wounding) and on days 3, 7, 10, and 14 after wound creation. Only mice with consistently elevated blood glucose levels of ≥16.7 mmol L^−1^ (300 mg dL^−1^) were included in the study. Blood glucose values were recorded at each time point and compared among treatment groups to ensure that any differences in wound healing were not attributable to variations in glycemic status. A total of 24 male db/db mice were included in the wound‐healing experiment and randomly allocated to four treatment groups using a computer‐generated randomization list: i) PBS (Control), ii) HeraDerm (commercial dressing), iii) free SCP‐1, and iv) Gel‐SCP‐1 (*n* = 6 mice per group). Before wound creation, mice were anesthetized with 2–3% isoflurane in oxygen, and the depth of anesthesia was confirmed by the absence of the pedal‐withdrawal reflex. For perioperative analgesia, buprenorphine (0.05 mg kg^−1^, subcutaneous injection) was administered approximately 30 min before surgery and every 12 h for 48 h postoperatively. After adequate anesthesia had been confirmed, the dorsal hair was removed, and a single full‐thickness excisional wound was created on the dorsum of each mouse using a sterile 8‐mm biopsy punch, extending through the panniculus carnosus. Treatments were applied topically to the wound bed at day 0 and subsequently at every 2 d. Wounds were covered with a sterile semi‐occlusive dressing to prevent dehydration and contamination, and animals were housed individually to minimize wound interference. Animals were monitored daily throughout the experiment for body weight, food and water intake, wound condition, activity level, mobility, grooming behavior, and other signs of pain or distress, including hunched posture, excessive grooming, self‐mutilation, and reduced mobility. Predefined humane endpoints included a loss of more than 20% of baseline body weight, persistent pain or distress unresponsive to appropriate intervention, severe wound infection, impaired ambulation, self‐mutilation, or a moribund condition. Animals meeting any of these criteria were to be euthanized immediately. No animals reached the predefined humane endpoints or required early euthanasia during the study. Digital photographs were taken on days 0, 3, 7, 10, and 14 with a scale bar included for calibration. Wound area was quantified using ImageJ software, and wound closure (%) was calculated as:

$$\;\mathrm{Wound}\;\mathrm{closure}\;\;\left(\%\right)=\frac{A_{0}-A_{t}}{A_{0}}\;\times 100\%$$

where *A*_0_ is the initial wound area and *A*_t_ is the wound area at the indicated time point. Wound‐area measurements, histological assessments, and quantitative image analyses were performed by investigators blinded to group allocation. Group identities were concealed using coded sample labels until the quantitative analyses had been completed.

### 2.13 Endothelial Tube Formation Assay

To assess the pro‐angiogenic effects of SCP‐1 and Gel‐SCP‐1 in vitro, a tube formation assay was performed using HUVECs. Growth factor‐reduced Matrigel (50 µL per well) was added to 96‐well plates and allowed to polymerize at 37°C for 30 min. HUVECs were then seeded at a density of 2 × 10^4^ cells per well in 100 µL of DMEM supplemented with 2% FBS. Cells were treated with hydrogel extracts (Blank Gel or Gel‐SCP‐1) or free SCP‐1 at an equivalent peptide concentration of 10 µm, while PBS was used as the vehicle control. After incubation for 6 h at 37°C in a humidified atmosphere containing 5% CO_2_, tube‐like structures were imaged using an inverted phase‐contrast microscope (Olympus IX73). The number of nodes, branching points, and total tube length in each field were quantified using ImageJ software with the Angiogenesis Analyzer plugin. All experiments were performed in triplicate, and at least five random fields were analyzed for each well.

### 2.14 Ex Vivo Aortic Ring Assay

The pro‐angiogenic activity of the treatments was further evaluated using an *ex vivo* rat aortic ring sprouting assay. Thoracic aortas were isolated from 8‐week‐old male Sprague‐Dawley rats under sterile conditions, and the surrounding periadventitial fat was carefully removed. The aortas were then cut into 1‐mm‐thick rings and embedded in 50 µL of growth factor‐reduced Matrigel in 48‐well plates. Each ring was overlaid with 200 µL of endothelial basal medium (EBM‐2) supplemented with 2% FBS and treated with Blank Gel extracts, free SCP‐1 (10 µm), Gel‐SCP‐1 extracts at an equivalent peptide concentration, or PBS as a control. The culture medium was refreshed every 48 h. After 7 d of incubation at 37°C in a humidified 5% CO_2_ atmosphere, microvessel sprouting was observed and photographed under a stereomicroscope (Leica M205 FA). The number and length of the sprouting microvessels were quantified using ImageJ software. Each experimental group included at least six aortic rings obtained from three independent animals.

### 2.15 Histological and Molecular Analysis

On day 14, mice were humanely euthanized by intraperitoneal injection of an overdose of pentobarbital sodium (150 mg kg^−1^), followed by cervical dislocation as a secondary method to confirm death. Wound tissues and major organs were subsequently collected for histological and molecular analyses. Samples were fixed in 4% paraformaldehyde, dehydrated, paraffin‐embedded, and sectioned.

#### 2.15.1 Histology

Hematoxylin and eosin (H&E) staining was performed following the manufacturer's instructions (G1120, Solarbio). The stained sections were used to evaluate re‐epithelialization, inflammatory cell infiltration, and granulation tissue thickness. Masson's trichrome staining was used to evaluate collagen deposition and maturation.

#### 2.15.2 Immunofluorescence

Tissue sections were subjected to antigen retrieval and blocked prior to incubation with primary antibodies against angiogenic markers (CD31 and VEGF), macrophage phenotype markers (iNOS and CD206), and STING at 4°C overnight, followed by fluorescent secondary antibodies and DAPI nuclear counterstaining.

#### 2.15.3 Gene Expression

Total RNA was extracted from wound tissues using TRIzol reagent, followed by reverse transcription to synthesize complementary DNA (cDNA) according to the manufacturer's protocol. Quantitative real‐time PCR (qRT‐PCR) was carried out on a CFX96 Real‐Time PCR System using Yeasen Hieff qPCR SYBR Green Master Mix (Cat. No. 11201) to quantify the mRNA expression of STING, Col1a1, Col3a1, Acta2 (α‐SMA), IL‐6, TNF‐α, IL‐1β, IL‐10, CD163, CD4, and CD8a. Relative expression levels were calculated using the 2^−ΔΔCt^ method, with β‐actin serving as the internal reference gene.

#### 2.15.4 Biosafety Evaluation

Major organs (e.g., heart, liver, spleen, lung, and kidney) were collected, fixed, paraffin‐embedded, and stained with H&E to assess potential systemic toxicity [37].

### 2.16 Oxidative Stress Biomarker Analysis

To assess the oxidative stress status of wound tissues in vivo, fresh wound samples were harvested on day 14, rinsed with ice‐cold PBS, and homogenized on ice in the corresponding assay buffers. After centrifugation at 12 000 × *g* for 15 min at 4°C, the supernatants were collected for biochemical analysis. Reduced glutathione (GSH) content was determined using a commercial GSH assay kit (Nanjing Jiancheng Bioengineering Institute, Nanjing, China) based on the DTNB colorimetric method. Malondialdehyde (MDA) levels, as an indicator of lipid peroxidation, were measured using a thiobarbituric acid reactive substances (TBARS) assay kit. Catalase (CAT) activity was quantified using a commercial assay kit based on the ammonium molybdate method. All measurements were performed in accordance with the manufacturers′ protocols, and the obtained values were normalized to total protein content measured by a BCA protein assay. Each group included *n* = 3 biologically independent wound samples.

### 2.17 Statistical Analysis

Data are presented as mean ± S.D. from at least three independent biological replicates unless otherwise indicated in the figure legends. The exact sample size (*n*) for each experiment is provided in the corresponding figure caption; n denotes biologically independent animals or experimental replicates rather than technical replicates. Normality was assessed using the Shapiro‐Wilk test. Comparisons between two groups were performed using an unpaired two‐tailed Student's t‐test, whereas comparisons among three or more groups were analyzed by one‐way ANOVA followed by Tukey's multiple‐comparisons test. Correlations were assessed using Pearson's correlation coefficient. All tests were two‐sided, and *p* < 0.05 was considered statistically significant. Significance is indicated as ns, not significant; *p < 0.05; **p < 0.01; and ***p < 0.001. Outliers were evaluated using the ROUT method (*Q* = 1%), and no data points were excluded from the analyses. Statistical analyses and graphing were performed using GraphPad Prism 9.0 (GraphPad Software).

## 3 Results and Discussion

### 3.1 Generative AI‐Driven Design and Molecular Mechanism of the STING‐Inhibitory Peptide SCP‐1

**FIGURE 1:**
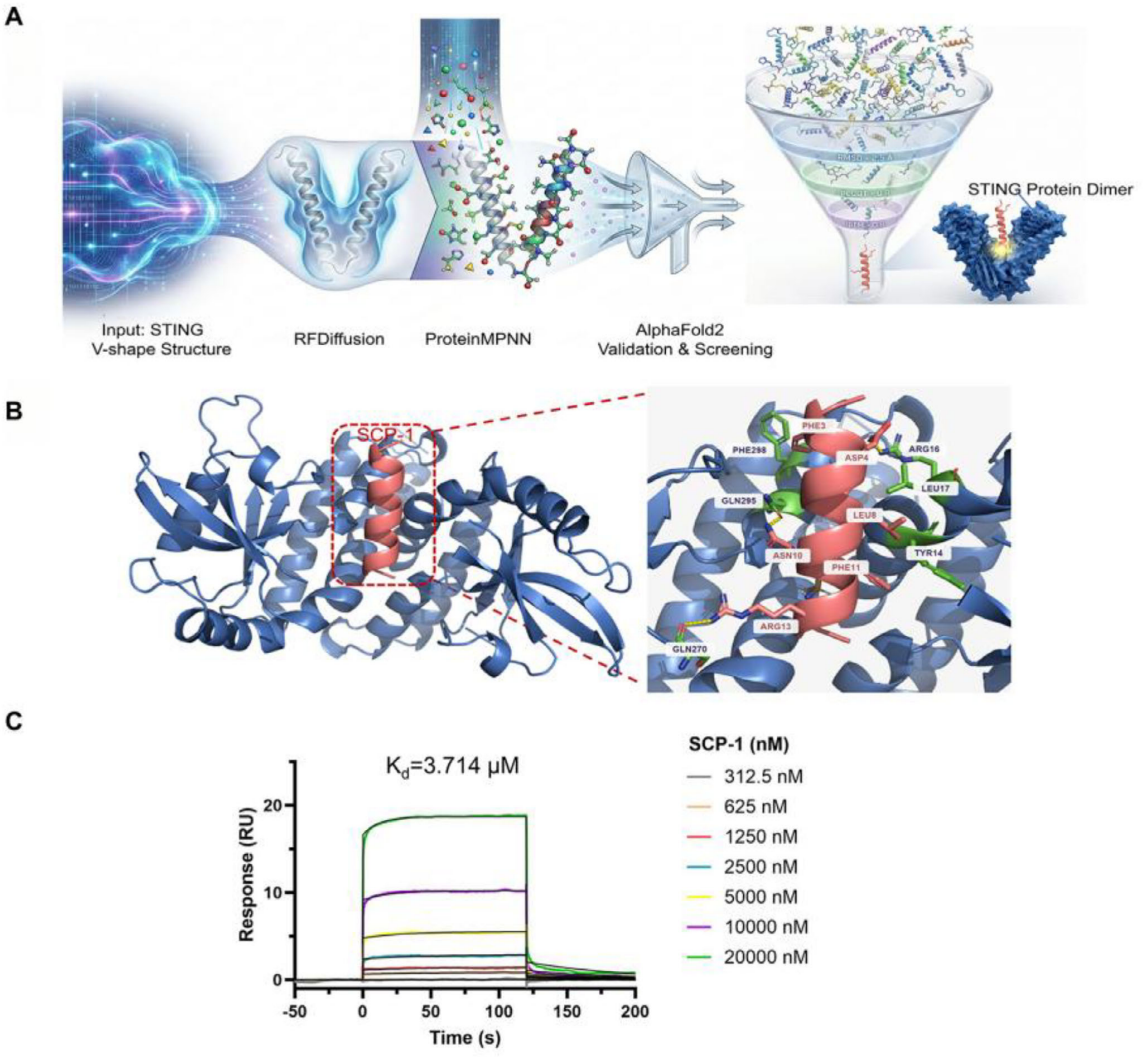
Generative AI‐enabled discovery and validation of the STING‐inhibitory peptide SCP‐1. (A) Schematic of the computational design and screening pipeline. Peptide backbones were generated de novo using RFDiffusion, sequence‐optimized by ProteinMPNN, and structurally evaluated with AlphaFold2‐multimer. Candidates were prioritized using hierarchical filtering criteria, including structural self‐consistency (backbone RMSD < 2.5 Å between the design model and the AlphaFold2 prediction), local structural confidence (mean pLDDT > 80), and interface confidence (ipTM > 0.8), leading to the identification of SCP‐1 as the top‐ranked candidate. (B) Predicted structure of the SCP‐1‐STING complex. SCP‐1 (salmon) adopts an α‐helical conformation and occupies the central cleft of the STING dimer (marine blue), consistent with stabilization of the inactive V‐shaped assembly. Insets highlight representative interfacial interactions, including hydrogen bonds (yellow dashed lines) and aromatic/hydrophobic contacts that together reinforce peptide binding at the dimer interface. (C) Surface plasmon resonance (SPR) analysis of SCP‐1 binding to human STING LBD. Sensorgrams show dose‐dependent binding of SCP‐1 to immobilize human STING LBD (inactive V‐shaped dimer) over a 7‐point twofold serial dilution series (0.3125‐=20 µm). Global fitting using a kinetic model yielded an equilibrium dissociation constant (*K*_d_) of 3.714 µm. Data are representative of *n* = 3 independent biological replicates.

A central innovation of this study lies in the use of generative artificial intelligence as the primary engine for therapeutic discovery, rather than as a secondary analytical tool. Because STING activation is governed by conformational rearrangement of its dimeric assembly, identifying an inhibitor that selectively stabilizes the inactive V‐shaped dimer is intrinsically challenging for conventional screening approaches. Traditional experimental library screening is poorly suited to this objective, not only because of the enormous peptide sequence space, but also because the desired function is state‐selective binding to a transient structural interface rather than simple target association. To overcome this limitation, we established a structure‐guided generative AI pipeline that directly designs peptides against the inactive STING dimer conformation (Figure 1A), thereby transforming a difficult empirical screening problem into a tractable state‐specific computational design task. The AI workflow integrates three complementary deep‐learning modules, each contributing a distinct and essential role in candidate discovery. RFDiffusion was first used to generate de novo peptide backbones geometrically compatible with the central cleft of the inactive STING dimer, enabling broad exploration of binding conformations beyond predefined ligand libraries. ProteinMPNN was then applied for inverse folding to assign amino acid sequences to these backbones, thereby improving sequence‐structure compatibility and increasing the likelihood that the designed interfaces would be physically realizable. Finally, AlphaFold2‐multimer was used for structural validation and prioritization of the designed peptide‐STING complexes. Candidates were filtered using stringent confidence criteria, including backbone self‐consistency, local structural confidence, and interface confidence. Through this hierarchical AI‐enabled triage, an initial design space of more than 10 000 computational candidates was rapidly compressed to fewer than 10 high‐confidence peptide leads, from which SCP‐1 (SAFDQLLLKNFRRLS) was selected as the top‐ranked design. This dramatic reduction in search space highlights the efficiency and selectivity conferred by AI, enabling lead identification within days rather than the prolonged iterative cycles typically required for experimental screening.

Importantly, the AI framework not only accelerated discovery, but also generated an explicit structural hypothesis for mechanisms. The predicted SCP‐1‐STING complex showed that SCP‐1 adopts an α‐helical conformation and inserts into the dimer cleft of inactive STING (Figure 1B). Structural inspection suggested that the peptide is stabilized by a defined interaction network involving hydrogen‐bonding contacts, together with hydrophobic and aromatic packing at the interface. This binding geometry is consistent with a conformation‐locking mechanism in which SCP‐1 acts as a molecular glue to reinforce the inactive STING dimer and impede the structural transition required for downstream activation. Thus, AI in this study did not merely produce a candidate sequence but also provided a mechanistically interpretable model linking peptide structure to biological function. The computational prediction was subsequently validated experimentally. SCP‐1 was synthesized by solid‐phase peptide synthesis, and its molecular identity and purity were confirmed by mass spectrometry and analytical RP‐HPLC (Figures S1 and S2). To directly test whether the AI‐designed peptide physically binds STING, we performed surface plasmon resonance analysis using recombinant human STING ligand‐binding domain. As shown in Figure 1C, SCP‐1 exhibited clear concentration‐dependent binding with an equilibrium dissociation constant (*K*_d_) of 3.714 µm, providing direct biophysical evidence that SCP‐1 specifically and reversibly engages STING. This result strongly supports the predictive value of the generative AI workflow, as the experimentally observed binding behavior is consistent with the AI‐predicted interaction mode and conformation‐stabilizing mechanism. Taken together, these findings establish SCP‐1 as a de novo AI‐designed STING‐binding peptide and highlight the broader significance of this work beyond identification of a single lead molecule.

### 3.2 Preparation and Physicochemical Characterization of the Dual‐Responsive Gel‐SCP‐1 Hydrogel

Although SCP‐1 showed direct STING binding and promising mechanistic activity, its translational use as a free peptide is likely constrained by limited stability in biologically relevant environments. In fresh human serum, SCP‐1 exhibited only moderate stability, with a half‐life of 4.2 ± 0.6 h, and the proportion of intact peptide progressively declined to 68%, 42%, and 15% after 2, 4, and 8 h, respectively, with less than 10% remaining at 24 h (Figure S3). Its susceptibility was even more pronounced in the presence of MMP‐9, a protease highly enriched in diabetic wounds, where the half‐life decreased to 1.8 ± 0.3 h and the peptide was nearly completely degraded within 8 h. These results indicate that, like many unmodified linear therapeutic peptides, free SCP‐1 is vulnerable to rapid proteolytic degradation under wound‐relevant conditions. This instability provided a strong rationale for developing a local delivery system capable of improving peptide retention and enabling microenvironment‐responsive release.

**FIGURE 2:**
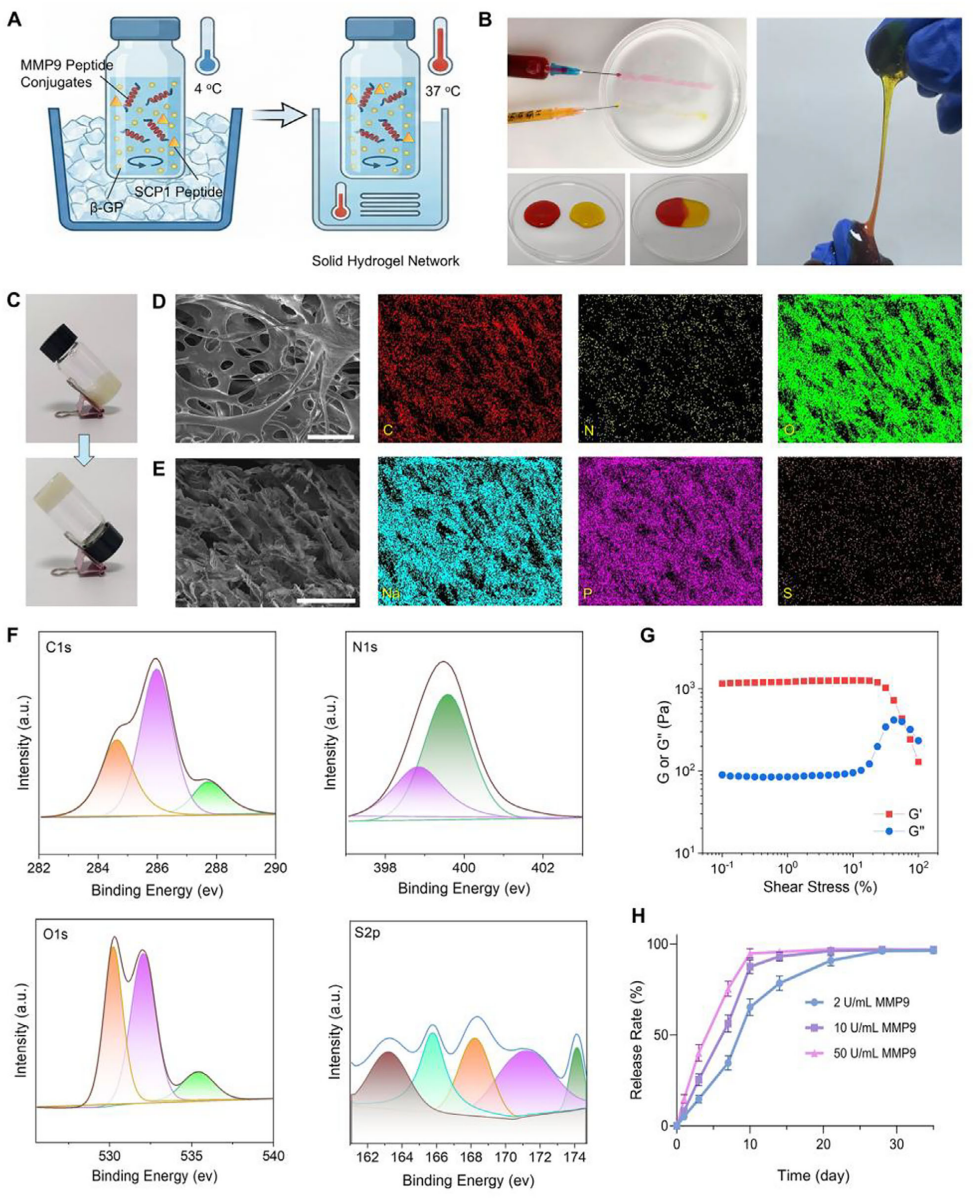
Preparation and physicochemical characterization of the dual‐responsive Gel‐SCP‐1 hydrogel. (A) Schematic illustration of the fabrication strategy in which an MMP‐9‐cleavable peptide is grafted onto chitosan and the sol‐gel transition is triggered by β‐glycerophosphate (β‐GP) upon warming (4 to 37°C). (B) Representative photographs showing injectability and self‐healing behavior: the precursor can be injected through a syringe, and two dyed hydrogel pieces fuse into an integrated gel that withstands stretching. (C) Vial inversion test confirming that temperature‐induced gelation at 37°C. (D‐E) SEM images of freeze‐dried hydrogels and corresponding EDS elemental mapping showing the spatial distribution of C, N, O, Na, P, and S. Scale bar: 100 µm. (F) High‐resolution XPS spectra of C1s, N1s, O1s, and S2p confirming the surface chemical composition and successful peptide conjugation. (G) Oscillatory amplitude sweep showing storage (*G*′) and loss (*G*″) moduli, indicating the linear viscoelastic region and gel robustness. (H) Enzyme‐responsive release of SCP‐1 from Gel‐SCP‐1 in PBS (pH 7.4) containing MMP‐9 (2, 10, or 50 U mL^−1^) at 37°C. Data are presented as mean ± S.D. (*n* = 3).

To address these limitations, we designed an injectable dual‐responsive hydrogel, termed Gel‐SCP‐1, that combines temperature‐triggered in situ gelation with MMP‐9‐responsive structural remodeling for localized peptide delivery in diabetic wounds. As illustrated in Figure 2A, chitosan (CS) was blended with an MMP‐9‐cleavable peptide‐grafted chitosan derivative (MMP9‐CS) and physically crosslinked with β‐glycerophosphate (β‐GP) to construct the hydrogel network. This design was intended to solve several interconnected challenges in diabetic wound treatment, including excessive protease activity, persistent inflammation, exudate accumulation, and poor local retention of therapeutics. At 4°C, the formulation remained as a flowable sol, facilitating homogeneous incorporation of SCP‐1 and convenient administration. Upon warming to 37°C, the precursor rapidly underwent sol‐gel transition to form a stable hydrogel network suitable for defect filling and conformal wound coverage. Macroscopic observations confirmed the favorable handling properties of the formulation. The precursor could be smoothly extruded through a syringe and readily deposited onto a substrate, indicating good injectability for topical administration (Figure 2B). In addition, two separately dyed gel pieces could merge into an integrated construct and remain intact under stretching, suggesting dynamic and reversible interactions within the network that support interfacial healing after mechanical disturbance. Consistent with these observations, vial inversion tests verified a clear temperature‐dependent sol‐gel transition: the formulation was flowable before heating but converted into a non‐flowing gel after incubation at 37°C (Figure 2C). Such behavior is particularly advantageous for diabetic wound management, where irregular wound geometries and body motion require both easy application and rapid in situ fixation. The hydrogel also exhibited shape adaptability and adhesion on dynamic skin surfaces (Figure S4A), further supporting its suitability for use in mechanically disturbed wound environments. The internal microstructure of Gel‐SCP‐1 was examined by scanning electron microscopy after lyophilization. The hydrogel displayed a highly interconnected porous architecture with lamellar features (Figure 2D,E), which is favorable for exudate absorption, nutrient and oxygen diffusion, and cellular infiltration during tissue repair. Elemental mapping further showed a homogeneous distribution of C, N, O, Na, P, and S throughout the matrix, indicating uniform incorporation of both β‐GP‐derived components and sulfur‐containing peptide motifs without obvious phase separation. This compositional uniformity is important because it suggests that the functional peptide‐derived structure is evenly integrated into the network, which should contribute to consistent mechanical properties and predictable release behavior.

The chemical composition of the hydrogel was further analyzed by X‐ray photoelectron spectroscopy. As shown in Figure 2F, the appearance of clear S2p signals supported successful incorporation of sulfur‐containing peptide/linker components into the matrix, while deconvolution of the N1s spectra revealed distinct nitrogen environments attributable to the chitosan backbone and peptide‐derived amide bonds. These findings provide further evidence that the MMP‐9‐cleavable peptide motif was successfully introduced into the hydrogel network, establishing the structural basis for enzyme‐responsive degradation and release. Successful grafting of the MMP‐9‐cleavable linker onto the CMCS backbone was additionally confirmed by ^1^H NMR spectroscopy, which showed new aromatic and aliphatic proton signals compared with unmodified CMCS (Figure S5). Thermogravimetric analysis further suggested substantial water‐associated mass loss in the low‐temperature region (Figure S6), consistent with a hydrated polymer network containing both free and bound water. This hydration state is likely beneficial not only for maintaining wound moisture and supporting molecular transport, but also for preserving an aqueous environment permissive for MMP‐9‐mediated network remodeling. The hydrogel also exhibited suitable mechanical properties for wound application. In oscillatory strain sweep measurements, the storage modulus (*G*′) remained higher than the loss modulus (*G*″) over the linear viscoelastic region (Figure 2G), indicating a predominantly elastic and mechanically stable network. Frequency‐dependent analysis similarly supported stable viscoelastic behavior across relevant timescales (Figure S7). These rheological features are consistent with a physically crosslinked chitosan/β‐GP system in which reversible noncovalent interactions provide sufficient structural robustness at rest while still allowing flow under applied shear, thereby explaining the observed injectability and self‐healing‐like fusion behavior. In addition, SCP‐1 exhibited negligible hemolytic activity at all tested concentrations, with hemolysis ratios remaining well below the 5% safety threshold (Figure S8), supporting its basic biocompatibility for biomedical use. A key functional feature of Gel‐SCP‐1 was its enzyme‐responsive release behavior. In vitro release studies showed that SCP‐1 release was markedly accelerated in the presence of MMP‐9 and increased in a clear enzyme concentration‐dependent manner at 2, 10, and 50 U mL^−1^ (Figure 2H). Because the MMP‐9‐cleavable peptide linker (GPLGVRGC) serves as a structural component of the hydrogel network, enzymatic cleavage is expected to disrupt network junctions, promote matrix loosening, and facilitate peptide diffusion. Consistent with this mechanism, the hydrogel remained largely intact in PBS but exhibited time‐dependent collapse and material loss in MMP‐9 solution (Figure S4C), supporting enzyme‐triggered degradation of the crosslinked matrix. Together, these results show that Gel‐SCP‐1 integrates two complementary responsive features: rapid thermogelation ensures local retention immediately after application, whereas elevated MMP‐9 in the diabetic wound microenvironment serves as an endogenous trigger for on‐demand matrix remodeling and peptide release. This design is therefore expected to improve the spatiotemporal delivery of SCP‐1 while reducing premature peptide loss caused by the poor stability of the free form.

### 3.3 In Vitro Biocompatibility and the Promotion of Endothelial Migration and Angiogenic Phenotypes

**FIGURE 3:**
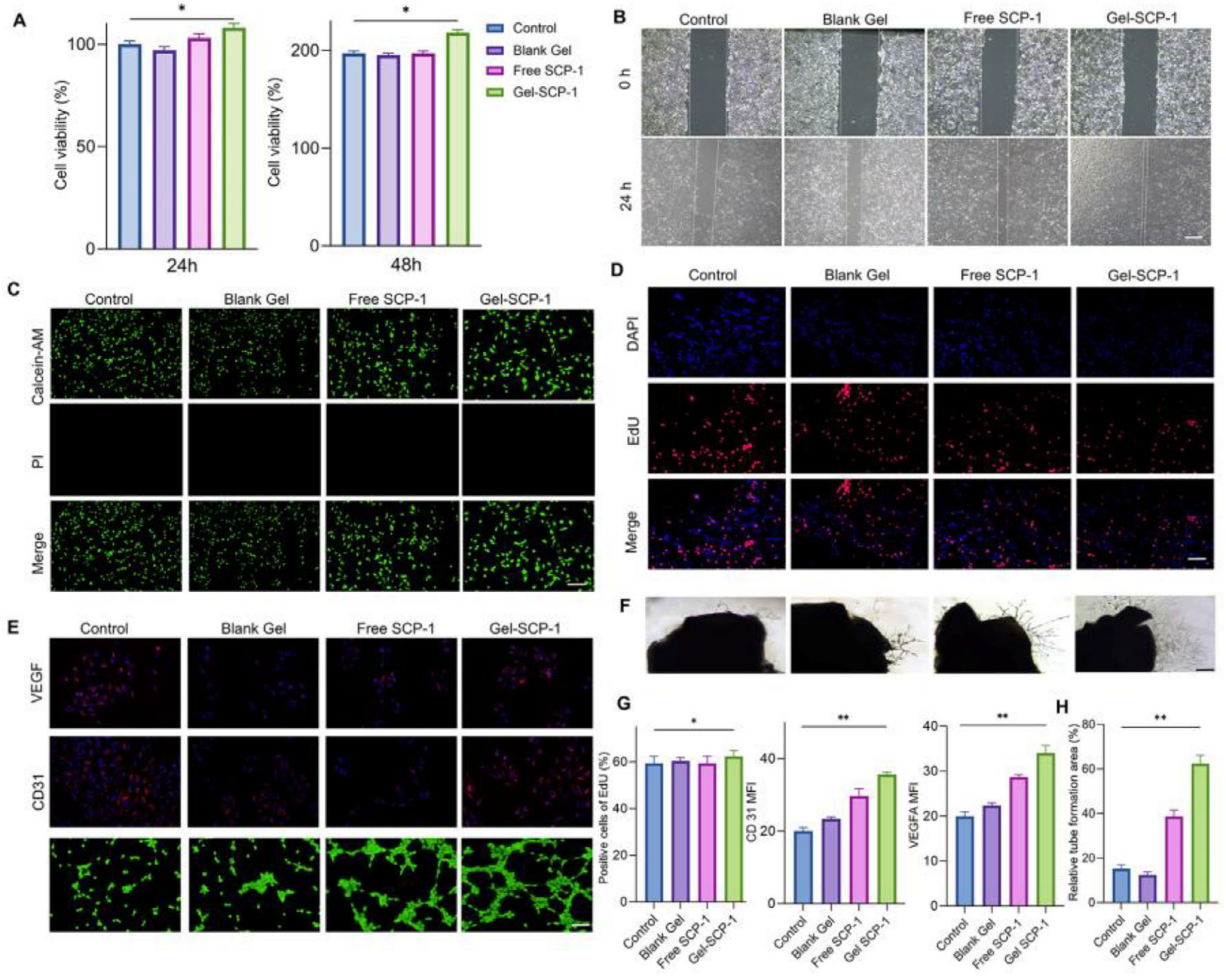
Gel‐SCP‐1 enhances endothelial compatibility and promotes angiogenic phenotypes. (A) CCK‐8 analysis of HUVEC viability after treatment with PBS (Control), Blank Gel, free SCP‐1, or Gel‐SCP‐1 for 24 and 48 h. (B) Representative scratch assay images showing HUVEC migration at 0 and 24 h after different treatments. Scale bars: 200 µm. (C) Live/dead staining of HUVECs following different treatments, with Calcein‐AM labeling live cells (green) and PI labeling dead cells (red). Scale bars: 100 µm. (D) Representative EdU staining images showing proliferating HUVECs, with EdU‐positive nuclei in red and DAPI‐stained nuclei in blue. Scale bars: 100 µm. (E) Representative images of VEGF‐A and CD31 immunofluorescence staining (red; nuclei counterstained with DAPI, blue) and Matrigel tube formation of HUVECs under different treatments. Scale bars: 100 µm. (F) Representative images of microvessel sprouting in the ex vivo rat aortic ring assay after different treatments. Scale bars: 500 µm. (G) Quantification of the percentage of EdU‐positive cells and the mean fluorescence intensity (MFI) of CD31 and VEGF‐A in HUVECs among different groups. (H) Quantification of the relative tube formation area in the Matrigel tube formation assay. All data are presented as mean ± S.D. (n = 3). Error bars represent S.D., and statistical significance was determined by one‐way ANOVA followed by Tukey's post‐hoc multiple comparison test. *p < 0.05, **p < 0.01.

Before evaluating its pro‐regenerative function, we first examined whether Gel‐SCP‐1 was cytocompatible with endothelial cells. HUVECs were treated with PBS (Control), Blank Gel, free SCP‐1, or Gel‐SCP‐1, and cell viability was assessed by CCK‐8. As shown in Figure 3A, none of the treatments reduced cell viability at either 24 or 48 h, indicating that both the hydrogel matrix and the peptide payload were well tolerated under the tested conditions. Notably, Gel‐SCP‐1 produced a modest but significant increase in cell viability compared with the control, suggesting that the hydrogel formulation not only avoids cytotoxicity but may also provide a microenvironment favorable for endothelial metabolic activity. Consistent with this, live/dead staining showed abundant Calcein‐AM‐positive live cells and almost no PI‐positive dead cells in all groups (Figure 3C), further confirming the excellent cytocompatibility of the system. We next investigated whether Gel‐SCP‐1 could promote endothelial behaviors essential for neovascularization. In the scratch assay, HUVECs treated with Gel‐SCP‐1 showed the most pronounced wound closure after 24 h, with a markedly narrower residual gap than those in the Control, Blank Gel, or free SCP‐1 groups (Figure 3B), indicating enhanced migratory capacity. EdU staining further showed more proliferating cells in the Gel‐SCP‐1 group than in the other groups (Figure 3D), and quantitative analysis confirmed a significant increase in the proportion of EdU‐positive cells (Figure 3G). These findings indicate that Gel‐SCP‐1 promotes both migration and proliferation of endothelial cells, two fundamental processes required for angiogenic repair.

To further assess angiogenic function, we performed Matrigel tube formation assays. Compared with the Control and Blank Gel groups, HUVECs treated with Gel‐SCP‐1 formed more extensive and better organized capillary‐like networks, whereas free SCP‐1 induced only a moderate improvement (Figure 3E). Quantification showed that the relative tube formation area was significantly increased in the Gel‐SCP‐1 group and was markedly higher than that in all other groups (Figure 3H). This result suggests that hydrogel‐mediated presentation of SCP‐1 provides a stronger pro‐angiogenic stimulus than the free peptide alone, likely because local retention and sustained release improve the effective bioavailability of the peptide. We then examined whether these functional changes were accompanied by an angiogenic endothelial phenotype. Immunofluorescence staining showed stronger signals for both CD31 and VEGF‐A in the Gel‐SCP‐1 group than in the Control, Blank Gel, and free SCP‐1 groups (Figure 3E). Quantitative analysis of mean fluorescence intensity confirmed that Gel‐SCP‐1 significantly increased CD31 and VEGF‐A expression, supporting enhanced endothelial activation and angiogenic signaling at the molecular level. To validate these pro‐angiogenic effects in a more physiologically relevant context, an *ex vivo* rat aortic ring sprouting assay was performed. After 7 days of culture, aortic rings treated with Gel‐SCP‐1 extracts exhibited robust microvessel outgrowth with denser and more extended sprouting structures than those observed in the other groups (Figure 3F). In contrast, Control and Blank Gel rings showed only limited peripheral sprouting, whereas free SCP‐1 induced an intermediate response. Taken together, these results demonstrate that Gel‐SCP‐1 possesses excellent endothelial biocompatibility and exerts a pronounced pro‐angiogenic effect across multiple levels, including endothelial viability, migration, proliferation, angiogenic marker expression, tube formation, and microvessel sprouting. Compared with free SCP‐1, the hydrogel formulation produced a more consistent and stronger angiogenic response, suggesting that the biomaterial carrier enhances the therapeutic performance of SCP‐1 by improving its local presentation and sustained availability.

### 3.4 Immunomodulatory Activity of Gel‐SCP‐1 Through Suppression of STING Signaling

Before examining its anti‐inflammatory mechanism, we first assessed whether SCP‐1 itself elicited overt innate immune activation. In a human PBMC stimulation assay, SCP‐1 did not induce significant release of IL‐6 or TNF‐α over the tested concentration range, with cytokine levels remaining comparable to those of the unstimulated control and far below those of the LPS‐treated positive control (Figure S9). These results suggest that the de novo designed peptide has low detectable in vitro immunogenicity under the tested conditions and support its suitability for local therapeutic application.

**FIGURE 4:**
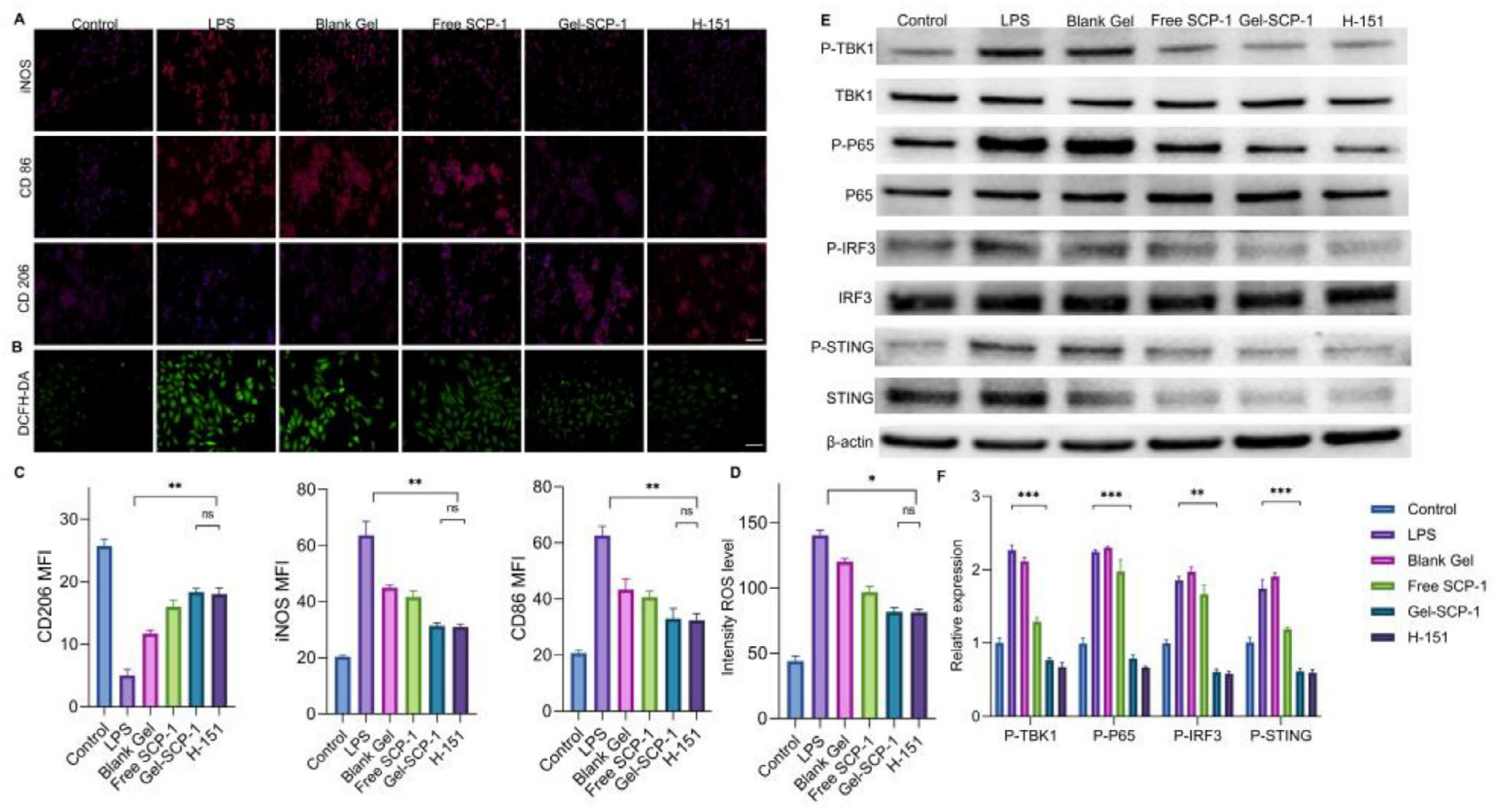
Gel‐SCP‐1 reprograms macrophage polarization and attenuates ROS‐associated cGAS‐STING signaling in vitro. RAW264.7 macrophages were stimulated with LPS (1 µg mL^−1^) and treated with Blank Gel, Free SCP‐1, Gel‐SCP‐1, or H‐151 (1 µm, STING‐specific positive control). (A) Representative immunofluorescence images of RAW264.7 macrophages stained for M1‐associated markers (iNOS and CD86) and M2‐associated marker (CD206). Target proteins are shown in red and nuclei are counterstained with DAPI (blue). Scale bar: 100 µm. (B) Intracellular ROS levels visualized by DCFH‐DA staining (green). Scale bar: 50 µm. (C) Quantitative analysis of MFI for CD206, CD86, and iNOS. (D) Quantitative analysis of ROS fluorescence intensity. (E) Representative Western blot analysis showing phosphorylated and total protein levels of STING, TBK1, IRF3, and NF‐κB p65 in RAW264.7 cells after the indicated treatments. β‐actin served as the loading control. (F) Densitometric quantification of relative protein expression/phosphorylation levels normalized to β‐actin (and to the Control group, if applicable). All data are presented as mean ± S.D. (*n* = 3). Error bars represent S.D., and statistical significance was determined by one‐way ANOVA followed by Tukey's post‐hoc multiple comparison test. *p < 0.05, **p < 0.01, ***p < 0.001, ns, not significant.

We next examined whether Gel‐SCP‐1 could regulate inflammatory macrophage responses under diabetic wound‐relevant conditions using LPS‐stimulated RAW264.7 cells. Chronic diabetic wounds are characterized by persistent M1‐like macrophage activation and failure to transition toward a reparative phenotype, and we therefore asked whether Gel‐SCP‐1 could reset this inflammatory imbalance. Immunofluorescence staining showed that LPS robustly induced expression of the M1‐associated markers iNOS and CD86, whereas treatment with Gel‐SCP‐1 markedly weakened both signals while increasing the M2‐associated marker CD206 (Figure 4A). Importantly, these qualitative changes were supported by quantitative analysis in Figure 4C, which showed significantly reduced mean fluorescence intensities of iNOS and CD86 together with an increase in CD206 in the Gel‐SCP‐1 group compared with the LPS and Blank Gel groups. Free SCP‐1 also partially reversed the inflammatory phenotype, but the effect was more pronounced when the peptide was delivered in the hydrogel. To determine whether this macrophage reprogramming was specifically related to STING inhibition rather than a nonspecific anti‐inflammatory effect, we included H‐151, a well‐established covalent STING antagonist, as a mechanistic reference control. Notably, the H‐151 group exhibited a highly similar pattern of marker regulation to the Gel‐SCP‐1 group, including reduced iNOS and CD86 and increased CD206, with no significant differences between the two groups in the corresponding quantitative analyses (Figure 4C). This pharmacological concordance strongly supports the conclusion that the immunomodulatory effect of Gel‐SCP‐1 is mechanistically linked to STING inhibition. Consistent with the phenotypic shift, cytokine analyses further showed that Gel‐SCP‐1 reduced the expression and secretion of the pro‐inflammatory mediators IL‐6, TNF‐α, and IL‐1β, while increasing the anti‐inflammatory cytokine IL‐10 (Figures S10 and S11). The cytokine profile of the H‐151 group closely resembled that of the Gel‐SCP‐1 group, further supporting a STING‐dependent mode of action.

Because oxidative stress is both a driver and a consequence of inflammatory macrophage activation, we next assessed intracellular ROS accumulation using DCFH‐DA staining. As shown in Figure 4B, LPS‐treated macrophages displayed strong fluorescence, consistent with marked ROS accumulation, whereas Gel‐SCP‐1 treatment substantially reduced the fluorescence signal. Quantitative analysis confirmed that intracellular ROS levels were significantly lower in the Gel‐SCP‐1 group than in the LPS and Blank Gel groups (Figure 4D). Again, H‐151 produced a comparable reduction in ROS, reinforcing the interpretation that the redox‐regulating effect of Gel‐SCP‐1 is linked to STING pathway inhibition. This observation is mechanistically relevant because excessive ROS can amplify inflammatory signaling, promote damage‐associated molecular signaling, and thereby sustain the chronic inflammatory state that impairs diabetic wound repair.

To further define the signaling mechanism underlying these effects, we analyzed the cGAS‐STING axis by western blotting. LPS stimulation increased the phosphorylation of STING, TBK1, IRF3, and NF‐κB p65, indicating activation of both the TBK1‐IRF3 arm and the NF‐κB inflammatory branch (Figure 4E). In contrast, Gel‐SCP‐1 markedly attenuated the phosphorylation of all four signaling nodes, as further summarized by densitometric analysis in Figure 4F. Importantly, the inhibitory pattern observed with Gel‐SCP‐1 was highly similar to that produced by H‐151, and no significant differences were observed between these two groups for the major phosphorylation readouts. These data provide strong mechanistic support that SCP‐1 suppresses inflammatory signaling primarily through inhibition of the STING pathway rather than through a broad off‐target immunosuppressive effect. In addition, while total TBK1, IRF3, and p65 levels remained relatively stable across groups, total STING abundance appeared reduced in the Gel‐SCP‐1 group compared with the LPS group, suggesting that Gel‐SCP‐1 may not only blunt acute phosphorylation events but also decrease upstream STING availability, thereby limiting overall pathway responsiveness. Taken together, these results indicate that Gel‐SCP‐1 exerts a multi‐level immunomodulatory effect, beginning with low intrinsic immunogenicity of the peptide itself and extending to macrophage repolarization, ROS suppression, and coordinated inhibition of the STING‐TBK1‐IRF3/NF‐κB signaling cascade. Importantly, the hydrogel formulation consistently outperformed free SCP‐1, suggesting that local retention and sustained peptide presentation enhance the biological efficacy of the AI‐designed STING inhibitor.

### 3.5 Gel‐SCP‐1 Accelerates Diabetic Wound Healing and Improves Tissue Regeneration In Vivo

**FIGURE 5:**
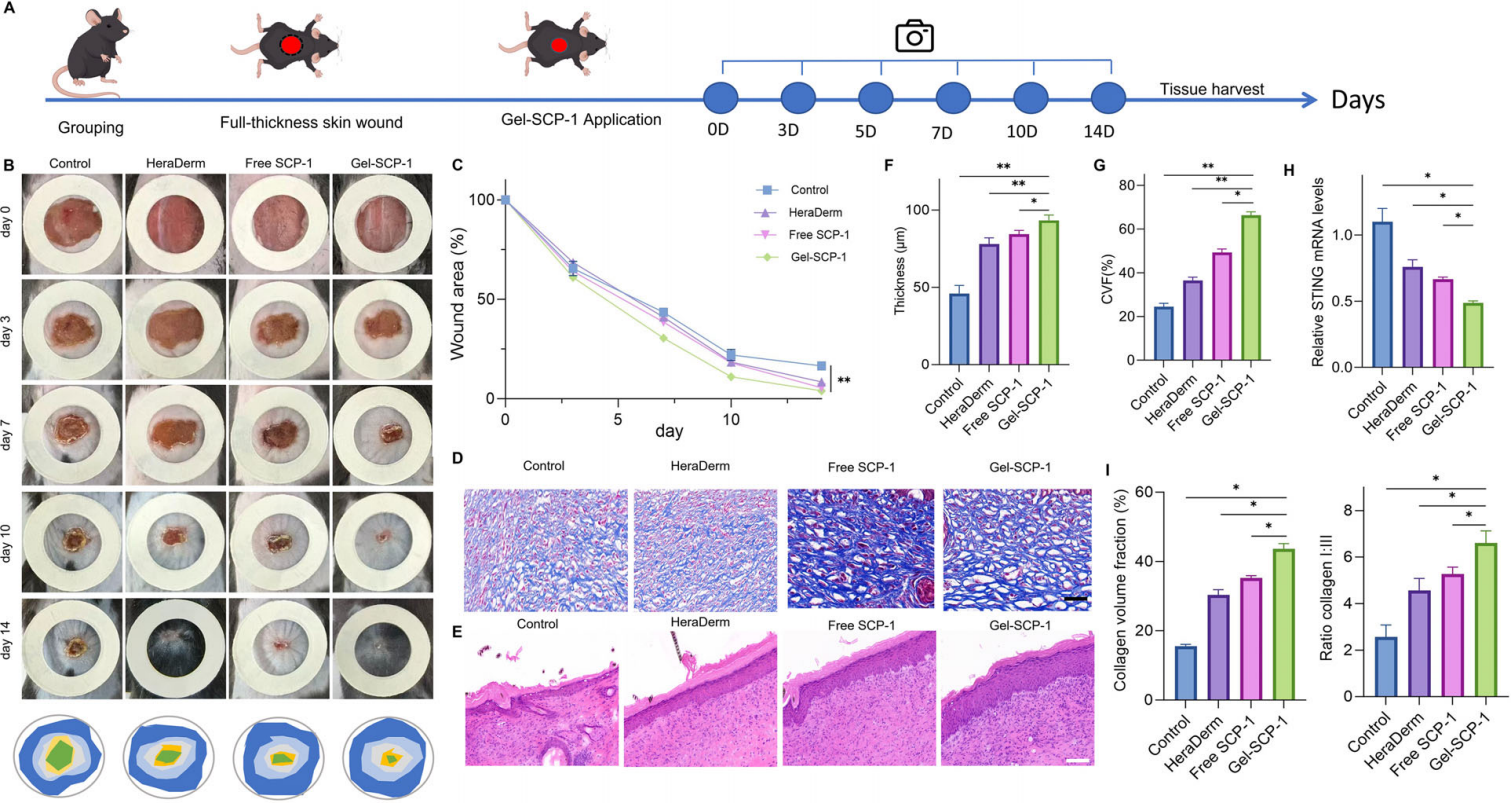
Gel‐SCP‐1 accelerates diabetic wound healing and improves tissue regeneration in vivo. (A) Schematic illustration of the experimental design and treatment timeline in the diabetic mouse full‐thickness excisional wound model. (B) Representative gross images of wounds treated with PBS (Control), HeraDerm, free SCP‐1, and Gel‐SCP‐1 on days 0, 3, 7, 10, and 14, with corresponding wound area tracings. (C) Quantitative analysis of normalized wound area over time. (D) Representative Masson's trichrome staining of wound sections on day 14. Scale bars: 100 µm. (E) Representative H&E staining of wound sections on day 14. Scale bars: 100 µm. (F) Quantification of epidermal thickness. (G) Quantification of collagen deposition in wound tissues. (H) Relative STING mRNA expression in wound tissues on day 14, measured by qRT‐PCR. (I) Additional quantitative analysis of collagen remodeling in wound tissues. (J) Quantification of the collagen I/III ratio on day 14. Data are presented as mean ± S.D. (*n* = 6). Statistical significance was assessed by one‐way ANOVA followed by Tukey's post‐hoc multiple comparison test. *p < 0.05, **p < 0.01.

We next assessed the therapeutic efficacy of Gel‐SCP‐1 in a full‐thickness excisional wound model in diabetic db/db mice (Figure 5A). Mice were treated with PBS (Control), the commercial dressing HeraDerm, free SCP‐1, or Gel‐SCP‐1, and wound repair was monitored for 14 d. Importantly, all animals maintained a stable diabetic state throughout the experiment: blood glucose levels remained consistently elevated, ranging from approximately 25 to 32 mmol L^−1^, with no significant differences among groups at any time point. This excludes differential glycemic control as a confounding factor and indicates that the improved healing observed in the Gel‐SCP‐1 group was attributable to treatment rather than altered metabolic status. Serial photographic monitoring revealed a clear treatment‐dependent difference in wound closure (Figure 5B). Although both HeraDerm and free SCP‐1 improved macroscopic healing compared with the Control group, Gel‐SCP‐1 consistently produced the fastest reduction in wound size over time. By day 14, Gel‐SCP‐1‐treated wounds were nearly closed and exhibited a smoother and more uniform surface, whereas visibly larger residual defects remained in the Control group. The wound tracing maps further supported this trend, showing the smallest residual wound area in the Gel‐SCP‐1 group. Quantification of normalized wound area (Figure 5C) confirmed that Gel‐SCP‐1 achieved the greatest degree of closure among all treatment groups.

Histological analysis demonstrated that this accelerated closure was accompanied by improved tissue regeneration. H&E staining showed that Gel‐SCP‐1‐treated wounds developed a thicker and more continuous neo‐epidermis than those in the Control, HeraDerm, and free SCP‐1 groups (Figure 5E), and the corresponding quantitative analysis showed a significant increase in epidermal thickness (Figure 5F). In parallel, Masson's trichrome staining revealed denser and more organized collagen deposition in Gel‐SCP‐1‐treated wounds (Figure 5D), whereas collagen fibers in the Control group appeared relatively sparse and disordered. Quantitative analysis confirmed that Gel‐SCP‐1 significantly increased collagen volume fraction (CVF) (Figure 5G). In addition, Gel‐SCP‐1 increased the collagen I/III ratio (Figure 5J), indicating a shift toward a more mature and mechanically reinforced extracellular matrix rather than an immature collagen III‐dominant repair tissue. A second collagen‐related quantitative panel (Figure 5I) showed the same overall trend, further supporting superior matrix deposition and remodeling in the Gel‐SCP‐1 group. Together, these results indicate that Gel‐SCP‐1 improved not only the speed of wound closure but also the structural quality of the regenerated tissue.

To connect these phenotypic improvements with the proposed mechanism of action, we next examined STING expression and broader transcriptional changes in day‐14 wound tissues. As shown in Figure 5H, Gel‐SCP‐1 produced the lowest STING mRNA level among all groups, consistent with the in vitro finding that SCP‐1 suppresses STING signaling in activated macrophages. To further characterize wound repair at the molecular level, we profiled a broader gene panel by qRT‐PCR (Figure S12). With respect to extracellular matrix remodeling, Gel‐SCP‐1 significantly upregulated Col1a1, whereas Col3a1 remained relatively similar across groups, resulting in an elevated Col1a1/Col3a1 ratio. Acta2 (α‐SMA) was also increased, consistent with enhanced myofibroblast differentiation, wound contraction, and stromal remodeling. The supporting inflammatory gene data further strengthened this interpretation. Gel‐SCP‐1 markedly reduced the mRNA levels of the pro‐inflammatory cytokines IL‐6, TNF‐α, and IL‐1β, while increasing IL‐10, indicating a shift from a persistent inflammatory state toward a more pro‐resolving milieu. In addition, the M2‐associated macrophage marker CD163 was significantly upregulated in the Gel‐SCP‐1 group, providing transcriptional evidence for reparative macrophage polarization. This observation is further supported by Figure S13, which complemented the CD206‐related macrophage findings described earlier. By contrast, CD4 and CD8a showed no significant differences among groups, suggesting that the therapeutic benefit of Gel‐SCP‐1 in this model is associated mainly with innate immune reprogramming rather than major changes in adaptive immune cell infiltration. The in vivo oxidative stress data also fit closely with this mechanism. As shown in Figure S14, the Control group displayed the lowest GSH levels and CAT activity together with the highest MDA content, consistent with the oxidative burden characteristic of diabetic wounds. HeraDerm and free SCP‐1 partially improved these parameters, but Gel‐SCP‐1 produced the strongest effect, with GSH levels ≈1.8‐fold higher than Control, CAT activity ≈1.6‐fold higher, and MDA reduced to approximately 45% of the Control level. These results indicate that Gel‐SCP‐1 restored antioxidant defense capacity and reduced lipid peroxidative damage in wound tissues. Importantly, these in vivo redox improvements are highly consistent with the reduced intracellular ROS observed in macrophages (Figure 4) and therefore reinforce the interpretation that suppression of STING‐associated inflammatory signaling helps break the feed‐forward loop between oxidative stress and chronic inflammation in diabetic wounds.

### 3.6 In Vivo Immunomodulation, Pro‐regenerative Remodeling, and Biosafety of Gel‐SCP‐1

**FIGURE 6:**
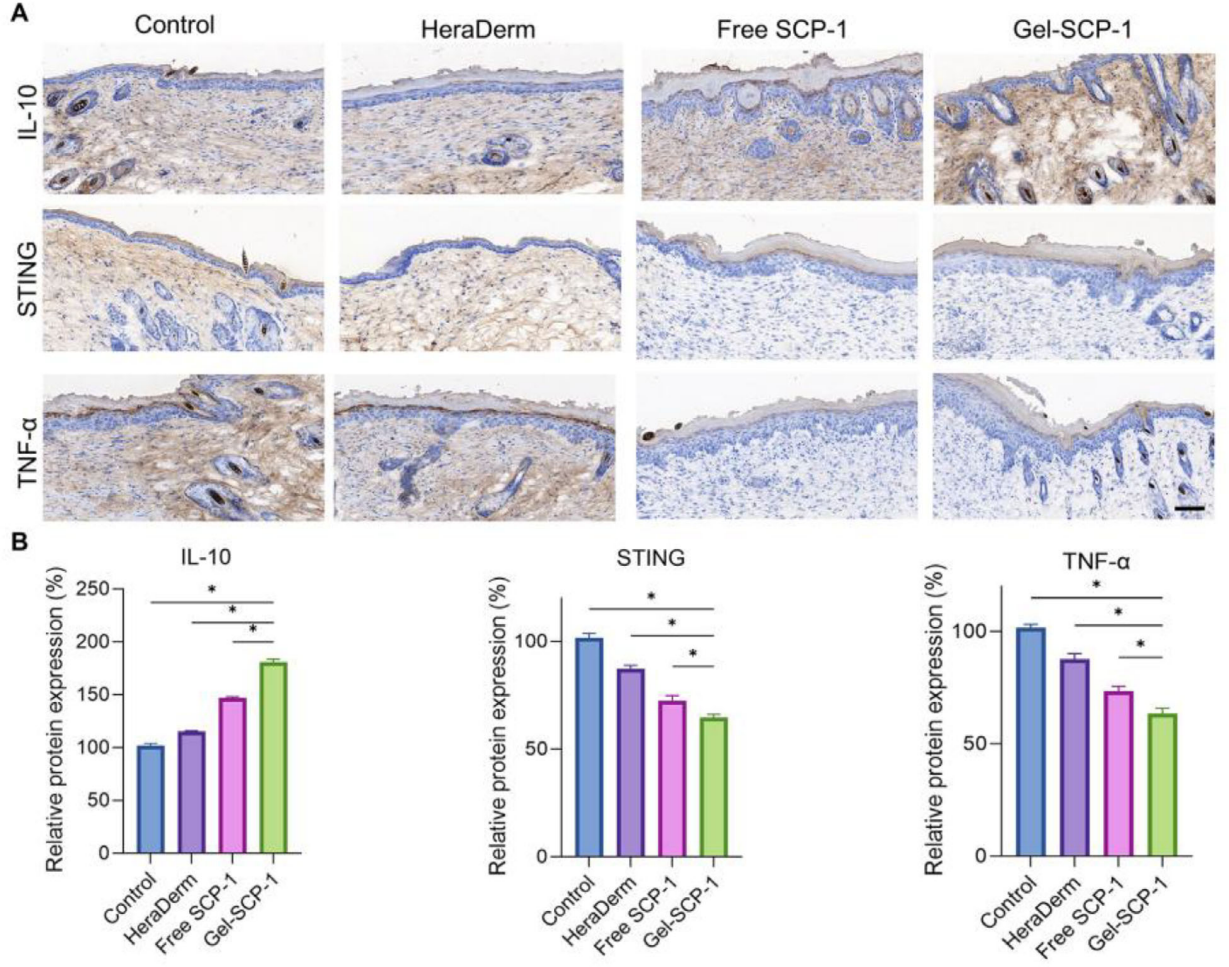
Immunohistochemical evaluation of inflammatory modulation in diabetic wound tissues on day 14. (A) Representative immunohistochemical staining of wound sections for the anti‐inflammatory cytokine IL‐10, the innate immune adaptor STING, and the pro‐inflammatory cytokine TNF‐α in the Control, HeraDerm, Free SCP‐1, and Gel‐SCP‐1 groups. Scale bar: 100 µm. (B) Semi‐quantitative densitometric analysis of relative protein expression (normalized to the Control). Gel‐SCP‐1 markedly increased IL‐10 while decreasing STING and TNF‐α compared with Control, HeraDerm, and Free SCP‐1, suggesting a shift toward an inflammation‐resolving wound microenvironment. Data are presented as mean ± S.D. (*n* = 6). Statistical significance was assessed by one‐way ANOVA followed by Tukey's post‐hoc multiple comparison test. *p < 0.05.

To further define how Gel‐SCP‐1 regulates the diabetic wound microenvironment in vivo, we performed immunohistochemical and immunofluorescence analyses on wound tissues collected on day 14. As shown in Figure 6A, Control wounds exhibited strong STING‐positive staining within the granulation tissue, whereas Gel‐SCP‐1 markedly reduced STING immunoreactivity. Semi‐quantitative analysis showed that STING staining in the Gel‐SCP‐1 group decreased to approximately 60% of the Control level, and was significantly lower than that in the Control, HeraDerm, and free SCP‐1 groups. By comparison, free SCP‐1 produced only a modest reduction in STING signal. These findings indicate that hydrogel‐mediated local retention and sustained presentation of SCP‐1 are important for maintaining effective peptide activity in the protease‐rich wound environment and thereby achieving robust inhibition of STING‐associated innate immune signaling in vivo. This suppression of STING was accompanied by broad remodeling of the inflammatory milieu. Gel‐SCP‐1 produced the weakest TNF‐α staining and the strongest IL‐10 staining among all treatment groups (Figure 6B). Semi‐quantitative analysis further showed that IL‐10 levels in the Gel‐SCP‐1 group increased to approximately 180% of the Control level, whereas TNF‐α was significantly reduced relative to the other groups. Together, these data indicate that Gel‐SCP‐1 shifts diabetic wounds away from a persistent pro‐inflammatory state toward a more immune‐resolving and regeneration‐permissive microenvironment. Consistent with this interpretation, immunofluorescence staining further revealed reduced iNOS expression together with elevated CD206 and VEGF signals in the Gel‐SCP‐1 group, indicating that suppression of inflammatory activation was accompanied by reparative macrophage polarization and enhanced pro‐angiogenic signaling within the granulation tissue.

The supporting histological data further reinforced this pro‐regenerative interpretation. Immunohistochemical staining of day‐14 wound sections for tissue remodeling and macrophage phenotype markers (Figure S15) showed markedly stronger collagen I (Col I) immunoreactivity in the Gel‐SCP‐1 group, whereas collagen III (Col III) staining remained relatively comparable among groups. This pattern is consistent with the increased Col I/Col III ratio and supports enhanced extracellular matrix maturation at the protein level. Densitometric analysis confirmed that Col I optical density was significantly higher in the Gel‐SCP‐1 group than in the Control and HeraDerm groups, while Col III did not differ significantly among groups. In parallel, CD163‐positive staining was substantially increased in Gel‐SCP‐1‐treated wounds, providing additional in situ protein‐level evidence for enhanced M2‐like macrophage polarization that complements the CD206 immunofluorescence findings. α‐SMA immunoreactivity was also elevated in the Gel‐SCP‐1 group, indicating increased myofibroblast differentiation and active stromal contraction, both of which are beneficial for wound closure and matrix remodeling.

We finally assessed systemic biosafety to determine whether topical Gel‐SCP‐1 treatment induced off‐target toxicity. Histological examination of major organs, including the heart, liver, spleen, lung, and kidney, showed no obvious pathological abnormalities or inflammatory lesions in the Gel‐SCP‐1 group compared with the Control group (Figure S16). Consistent with this, complete blood count analysis and serum biochemical testing, including alanine aminotransferase, aspartate aminotransferase, blood urea nitrogen, and creatinine, revealed no significant differences among groups, and all values remained within the normal physiological range for diabetic mice (Table S2). These findings support favorable in vivo biocompatibility and indicate that topical Gel‐SCP‐1 treatment did not induce detectable systemic hematological, hepatic, or renal toxicity over the course of the study. Overall, these results demonstrate that Gel‐SCP‐1 remodels the diabetic wound microenvironment at multiple levels. It suppresses STING‐associated inflammatory signaling, reduces pro‐inflammatory cytokine expression, promotes reparative macrophage polarization, enhances angiogenic signaling, and supports matrix maturation, while maintaining good systemic biosafety.

## 4 Conclusion

In summary, this work establishes an integrated AI‐to‐biomaterial strategy for precision immunoregenerative therapy in diabetic wounds. A structure‐guided generative AI pipeline identified SCP‐1 as a conformation‐locking peptide that inhibits STING by stabilizing its inactive dimeric state, addressing a target class that is difficult to access through conventional screening or small‐molecule design. Incorporation of SCP‐1 into a dual‐responsive hydrogel enabled localized retention and MMP‐9‐responsive release within the pathological wound microenvironment. As a result, Gel‐SCP‐1 suppressed STING‐associated inflammatory and oxidative stress signaling, promoted reparative macrophage polarization, enhanced angiogenic activity, and improved tissue regeneration. In diabetic mice, these effects translated into accelerated wound closure, improved re‐epithelialization and collagen remodeling, and a more pro‐resolving wound niche, with no detectable systemic toxicity. Beyond diabetic wound repair, this study provides a broadly applicable framework for coupling generative AI‐enabled discovery of conformation‐selective ligands with disease‐responsive biomaterial delivery, thereby opening new opportunities for targeting conformationally regulated innate immune pathways in chronic inflammatory diseases.

## Author Contributions

L.D.Z., X.L., D.L., and X.D.W. conceived the idea, designed the study, and supervised the project. X.C. developed the computational pipeline and performed the AI‐driven peptide design and screening (RFDiffusion and ProteinMPNN). X.Y.L. and H.F. carried out the material synthesis, characterization, and performed the in vitro and in vivo experiments. Z.W. assisted with material analysis and data visualization. R.H.Z. provided technical support and participated in data analysis. X.Y.L. and H.F. wrote the original draft. L.D.Z., X.L., D.L., and X.D.W. reviewed, edited, and finalized the manuscript. All authors have read and agreed to the published version of the manuscript.

## Funding

This work was supported by the Characteristic Disease Biobank Project of the Shanghai Ninth People's Hospital Affiliated to Shanghai Jiao Tong University School of Medicine (YBK202511); Hainan Provincial Natural Science Foundation of China (No. 326MS0427).

## Ethics Statement

All animal procedures were approved by the Institutional Animal Care and Use Committee of Shanghai Ninth People's Hospital, Shanghai Jiao Tong University School of Medicine, and conducted following ARRIVE 2.0 guidelines and applicable institutional and national regulations. Animals were monitored daily using predefined humane endpoints, and no early euthanasia was required. On day 14, mice were euthanized with an overdose of pentobarbital sodium and subsequent cervical dislocation.

Human image publication: The photograph in FigureS4B shows a co‐author’s hand, solely to demonstrate the hydrogel’s conformal adhesion upon finger movement. No clinical intervention, human sample collection or identifiable personal information is involved. Written informed consent for image publication was acquired prior to shooting, and no recognizable personal features are presented.

Human‐derived biological materials including commercial human serum (Sigma‐Aldrich, St. Louis, MO, USA), PBMCs and HUVECs (Shanghai iCell Bioscience Inc., Shanghai, China) were purchased commercially without donor‐identifiable information. As documented by vendors, samples were obtained with proper ethical approval and informed consent. No human participants were recruited by the authors, and no identifiable personal information was available.

## Conflicts of Interest

The authors declare no conflicts of interest.

## Supporting information




**Supporting File 1**: advs76849‐sup‐0001‐SuppMat.docx.

**Supporting File 2**: advs76849‐sup‐0002‐Data.zip.

## Data Availability

All supporting data for these study findings described in this paper are included in the main text and supplementary information, with the source data provided alongside this paper.
