# Supplementary material for: AI Designed Conformation Locking Peptides Target STING to Restore Diabetic Wound Healing
Source: Adv Sci (Weinh). 2026 Aug 7;13(59):e76849. doi: 10.1002/advs.76849 (PMC13451144; doi:10.1002/advs.76849)
Supplement: Supplementary file 1 [file ADVS-13-e76849-s001.docx]

**AI Designed Conformation Locking Peptides Target STING to Restore Diabetic Wound Healing**

Xinyu Li^1+^, Haojie Fu^2+^, Zhe Wang^3^, Xuanzhou Chen^4^, Xudong Wang^2*^; Louis D. Zhang^5,6*^, Xiang Li^7*^; Datao Li^1,8*^

^1^ Department of Plastic and Reconstructive Surgery, Shanghai Ninth People’s Hospital, Shanghai Jiao Tong University School of Medicine, Shanghai, China.

^2^ Department of Oral and Craniomaxillofacial Surgery, Shanghai Ninth People’s Hospital, Shanghai Jiao Tong University School of Medicine, Shanghai, China.

^3^ Institute of Bioengineering, College of Chemical and Biological Engineering, Zhejiang University, Hangzhou 310037, China.

^4^ School of Electrical and Computer Engineering, Georgia Institute of Technology, Atlanta, GA 30332, USA.

^5^ The Wallace H. Coulter Department of Biomedical Engineering, Georgia Institute of Technology and Emory University, Atlanta, Georgia 30332, USA.

^6^ Power Dream America, Inc., Peachtree Corners, GA 30071, USA.

^7^ School of Pharmacy, Second Military Medical University, 325 Guohe Road, Shanghai, 200433, China.

^8^ Department of Plastic and Reconstructive Surgery, Hainan Western Central Hospital, Danzhou 571700, Hainan, China

**Figure S1.** Purity analysis of SCP-1 by high-performance liquid chromatography (HPLC). Representative analytical reverse-phase HPLC chromatogram of the synthesized SCP-1 peptide. Integration of the major peak indicates 98.7% purity, confirming high peptide quality suitable for subsequent biological evaluation.

**Figure S2.** Mass spectrometry characterization of the synthesized SCP-1 peptide. The spectrum shows the molecular ion peak of the purified peptide. The observed mass (1822.10 Da) is consistent with the calculated theoretical mass (1823.13 Da), confirming successful synthesis and the identity of SCP-1.

**Figure S3.** Stability of SCP-1 in human serum and against MMP-9. Bar graphs showing the percentage of intact SCP-1 remaining after incubation in (A) fresh human serum or (B) PBS containing recombinant human MMP-9 (100 ng/mL) at 37 °C for the indicated time periods. Data are presented as mean ± SD (n = 3 independent experiments). Half-lives (t₁/₂) were calculated by one-phase exponential decay fitting and are indicated in each panel.

**Figure S4.** Macroscopic properties and enzyme-responsive degradation of Gel-SCP-1. (A) Representative photographs demonstrating the remoldability of Gel-SCP-1, which was reshaped from a bulk gel into a star-shaped geometry using a mold. (B) Photographs showing the conformal tissue adhesion of Gel-SCP-1 on a finger during dynamic movements, including bending and fist formation, without visible detachment. (C) Representative degradation images of Gel-SCP-1 incubated in PBS (pH 7.4) or MMP-9 solution (50 U/mL) at 37 °C (at the indicated time points). The hydrogel remains relatively stable in PBS but exhibits accelerated disintegration/structural collapse in the presence of MMP-9, confirming enzyme-responsive degradation. The hand shown in panel B belongs to one of the authors, from whom written informed consent for publication of the image was obtained.

**Figure S5.** ^1^H NMR spectra verification of MMP-9-cleavable peptide linker grafting onto CMCS.

**Figure S6.** Thermal stability analysis by thermogravimetric analysis. TGA curve of lyophilized hydrogel recorded from 25 to 600 °C. The initial weight loss is attributed to the removal of adsorbed/bound water, followed by stepwise polymer-network decomposition at elevated temperatures, reflecting the thermal stability of the material.

**Figure S7.** Rheological frequency sweep of Gel-SCP-1. Storage modulus (G’) and loss modulus (G’’) as a function of angular frequency (0.1-100 rad/s). G’ remains higher than G’’ throughout the tested range, indicating predominantly elastic, solid-like behavior and a stable crosslinked network with good frequency-independent viscoelasticity.

**Figure S8.** Hemocompatibility assessment of Gel-SCP-1. Hemolysis ratios of red blood cells after incubation with blank gel, free SCP-1, and Gel-SCP-1, calculated from the absorbance of hemoglobin in the supernatant. The positive and negative controls represent complete and negligible hemolysis, respectively. Gel-SCP-1 shows a hemolysis rate <5%, indicating good blood compatibility (Inset: representative images). Data are presented as mean ± SD (n = 3). Statistical significance was assessed by one-way ANOVA followed by Tukey's post-hoc multiple comparison test.

**Figure S9. *In vitro* immunogenicity assessment of SCP-1.** (A) IL-6 release from human PBMCs after 48 h stimulation with SCP-1 at indicated concentrations. (B) TNF-α release under the same conditions. Data are presented as mean ± SD (n = 3 independent experiments using PBMCs from different donors). Error bars represent SD. Statistical significance was determined by one-way ANOVA followed by Tukey's post-hoc multiple comparison test. **p* < 0.05 vs. LPS control; ns, not significant (p ≥ 0.05) vs. negative control.

**Figure S10.** Effects of Gel-SCP-1 on inflammatory cytokine gene expression. Quantitative real-time PCR (qRT-PCR) analysis of IL-1β, IL-6, STING, and TNF-α mRNA levels in LPS-stimulated RAW264.7 macrophages treated with Blank Gel, Free SCP-1, Gel-SCP-1, or H-151 (1 μM, STING-specific positive control). Data are presented as mean ± SD (n = 3 biologically independent experiments). Error bars represent SD. Statistical significance was determined by one-way ANOVA followed by Tukey's post-hoc multiple comparison test. **p* < 0.05; ns, not significant (*p* ≥ 0.05).

**Figure S11.** Effects of Gel-SCP-1 on inflammatory cytokine secretion. ELISA quantification of IL-1β, IL-6, IL-10, and TNF-α protein levels in the culture supernatants of RAW264.7 macrophages after the indicated treatments. H-151 (1 μM) was included as a STING-specific positive control. Gel-SCP-1 significantly suppresses pro-inflammatory cytokines while upregulating anti-inflammatory IL-10 compared to the LPS group. No statistically significant differences were observed between the H-151 and Gel-SCP-1 groups for any cytokine measured. Data are presented as mean ± SD (n = 3 biologically independent experiments). Error bars represent SD. Statistical significance was determined by one-way ANOVA followed by Tukey's post-hoc multiple comparison test. **p* < 0.05; ns, not significant (*p* ≥ 0.05).

**Figure S12.** qRT-PCR analysis of wound-related gene expression in day-14 diabetic wound tissues. Relative mRNA expression levels of extracellular matrix remodeling markers (Col1a1, Col3a1, and α-SMA), pro-inflammatory cytokines (IL-6, TNF-α, and IL-1β), anti-inflammatory cytokine (IL-10), M2 macrophage marker (CD163), and adaptive immune markers (CD4 and CD8a) in wound tissues from Control, HeraDerm, Free SCP-1, and Gel-SCP-1 groups on day 14. Data are presented as mean ± SD (n = 6 biologically independent animals per group). Error bars represent SD. Statistical significance was assessed by one-way ANOVA followed by Tukey's post-hoc multiple comparison test. **p* < 0.05, ***p* < 0.01; ns, not significant (*p* ≥ 0.05).

**Figure S13.** Immunofluorescence analysis of macrophage polarization and angiogenic marker expression in wound tissues. Representative immunofluorescence images of wound sections harvested on day 14. Staining for macrophage markers shows a significant decrease in pro-inflammatory iNOS and an increase in pro-reparative CD206 in the Gel-SCP-1 group compared to controls. Staining for Vascular Endothelial Growth Factor indicates enhanced angiogenic signal intensity in the Gel-SCP-1 treated wounds. Nuclei were counterstained with DAPI (blue). These results confirm that hydrogel creates a pro-regenerative microenvironment by coupling immune modulation with neovascularization. Scale bar: 50 µm.

**Figure S14. Oxidative stress biomarker assessment in day-14 diabetic wound tissues.** Bar graphs of catalase (CAT) activity, malondialdehyde (MDA) content, and reduced glutathione (GSH) levels in Control, HeraDerm, Free SCP-1, and Gel-SCP-1 treated diabetic mice. Gel-SCP-1 significantly elevated CAT and GSH levels and reduced MDA content. Data are presented as mean ± SD (n = 6 biologically independent animals per group). Error bars represent SD. Statistical significance was assessed by one-way ANOVA followed by Tukey's post-hoc multiple comparison test. ***p* < 0.01; ns, not significant.

**Figure S15. Immunohistochemical validation of tissue remodeling and macrophage phenotype markers in diabetic wound tissues on day 14.** (A) Representative immunohistochemical staining of wound sections for collagen I (Col I), collagen III (Col III), CD163 (M2-associated macrophage marker), and α-SMA (myofibroblast marker) in the Control, HeraDerm, Free SCP-1, and Gel-SCP-1 groups. Scale bar: 100 μm. (B) Semi-quantitative densitometric analysis of relative mean optical density for CD163, α-SMA, Col I, and Col III (normalized to the Control group). Data are presented as mean ± SD (n = 6 biologically independent animals per group). Error bars represent SD. Statistical significance was assessed by one-way ANOVA followed by Tukey's post-hoc multiple comparison test. **p* < 0.05, ***p* < 0.01; ns, not significant (*p* ≥ 0.05).

**Figure S16.** *In vivo* biosafety evaluation of the hydrogel dressing. Representative H&E-stained images of major organs (*i.e.*, heart, liver, spleen, lung, and kidney) collected from diabetic mice after 14 days of treatment (Control, HeraDerm, Free SCP-1, and Gel-SCP-1). No evident histopathological abnormalities, including necrosis, congestion, or inflammatory cell infiltration, were observed in the Gel-SCP-1 group compared with the Control group, indicating favorable systemic biocompatibility. Scale bar: 100 µm.

| **Table S1. Computational screening dataset** | | | | | | | | |
| --- | --- | --- | --- | --- | --- | --- | --- | --- |
| ID | SEQ | TARGET | DOMAIN | RMSD | SCORE | plDDT | PTM | PAE |
| BUCKET | SAFDQLLLKNFRRLS | STING inactive 6MXE | A1-1000/B1-1000:15-15 | 2.013 | 1.013 | 0.826 | 0.826 | 0.222 |
| 20240129-0421 | SIKERMERLGFRLTD | STING inactive 6MXE | A1-1000/B1-1000:15-15 | 2.185 | 1.125 | 0.654 | 0.727 | 11.115 |
| 20240129-0423 | SIRERMERLGFRLTD | STING inactive 6MXE | A1-1000/B1-1000:15-15 | 2.157 | 1.158 | 0.638 | 0.72 | 11.33 |
| 20240127-0004 | SSQDELLLRSYEISS | STING inactive 6MXE | A1-1000/B1-1000:15-15 | 3.704 | 1.314 | 0.577 | 0.665 | 12.896 |
| 20240127-0003 | SSRDELLLRSYLISS | STING inactive 6MXE | A1-1000/B1-1000:15-15 | 3.522 | 1.233 | 0.576 | 0.668 | 12.977 |
| 20240127-0002 | SPQDELLRRSYEISS | STING inactive 6MXE | A1-1000/B1-1000:15-15 | 2.689 | 1.239 | 0.568 | 0.64 | 12.958 |
| 20240129-0525 | SLLREILRSLAS | STING inactive 6MXE | A1-1000/B1-1000:12-12 | 59.999 | 1.900 | 0.527 | 0.125 | 28.465 |
| 20240129-0563 | SAVDELLRSFLK | STING inactive 6MXE | A1-1000/B1-1000:12-12 | 53.457 | 1.297 | 0.441 | 0.247 | 25.503 |
| 20240127-0023 | SEASEARRARYSKLS | STING inactive 6MXE | A1-1000/B1-1000:15-15 | 26.233 | 1.391 | 0.416 | 0.427 | 21.131 |
| 20240127-0040 | DRERRRRVENARNLF | STING inactive 6MXE | A1-1000/B1-1000:15-15 | 37.591 | 1.574 | 0.409 | 0.159 | 27.446 |
| 20240129-0569 | MRLHKLFEERRE | STING inactive 6MXE | A1-1000/B1-1000:12-12 | 29.884 | 1.528 | 0.393 | 0.432 | 19.231 |
| 20240127-0018 | SEASEARRERYRRLS | STING inactive 6MXE | A1-1000/B1-1000:15-15 | 26.3 | 1.325 | 0.381 | 0.357 | 21.97 |
| 20240127-0588 | MSLADLVSAILK | STING inactive 6MXE | A1-1000/B1-1000:12-12 | 53.574 | 1.116 | 0.369 | 0.141 | 27.757 |
| 20240127-0581 | MSLADLVSAILK | STING inactive 6MXE | A1-1000/B1-1000:12-12 | 53.574 | 1.180 | 0.369 | 0.141 | 27.757 |
| 20240129-0523 | RREQMLRTAKLI | STING inactive 6MXE | A1-1000/B1-1000:12-12 | 37.841 | 1.513 | 0.359 | 0.208 | 26.207 |
| 20240129-0527 | SAFREFLRSLLS | STING inactive 6MXE | A1-1000/B1-1000:12-12 | 46.506 | 1.780 | 0.353 | 0.182 | 26.848 |
| 20240129-0553 | AVSQDLENWVFE | STING inactive 6MXE | A1-1000/B1-1000:12-12 | 66.503 | 1.467 | 0.347 | 0.121 | 28.476 |
| 20240129-0389 | TESAQERLEEILNGL | STING inactive 6MXE | A1-1000/B1-1000:15-15 | 19.01 | 1.524 | 0.347 | 0.299 | 22.125 |
| 20240127-0047 | SRRRQRMVENARNLF | STING inactive 6MXE | A1-1000/B1-1000:15-15 | 33.502 | 1.588 | 0.344 | 0.14 | 27.152 |
| 20240129-0564 | SEVDELLRKLLQ | STING inactive 6MXE | A1-1000/B1-1000:12-12 | 47.418 | 1.357 | 0.343 | 0.187 | 26.064 |
| 20240129-0562 | SEVDELLRKFLE | STING inactive 6MXE | A1-1000/B1-1000:12-12 | 47.621 | 1.320 | 0.335 | 0.166 | 26.546 |
| 20240129-0521 | MRKKMLETAKII | STING inactive 6MXE | A1-1000/B1-1000:12-12 | 37.085 | 1.388 | 0.335 | 0.195 | 26.277 |
| 20240127-0582 | MSLADLVSAMLK | STING inactive 6MXE | A1-1000/B1-1000:12-12 | 50.052 | 1.185 | 0.334 | 0.169 | 27.031 |
| 20240127-0578 | MSLADLVSAMLK | STING inactive 6MXE | A1-1000/B1-1000:12-12 | 50.052 | 1.175 | 0.334 | 0.169 | 27.031 |
| 20240127-0577 | MSLADLVSAMLK | STING inactive 6MXE | A1-1000/B1-1000:12-12 | 50.052 | 1.151 | 0.334 | 0.169 | 27.031 |
| 20240127-0031 | SEASEARRERYRKLS | STING inactive 6MXE | A1-1000/B1-1000:15-15 | 26.378 | 1.349 | 0.329 | 0.23 | 23.874 |
| 20240127-0028 | SEASEARRERYRKLS | STING inactive 6MXE | A1-1000/B1-1000:15-15 | 26.378 | 1.352 | 0.329 | 0.23 | 23.874 |
| 20240129-0526 | DELEELLKSFRS | STING inactive 6MXE | A1-1000/B1-1000:12-12 | 13.148 | 1.943 | 0.328 | 0.25 | 22.627 |
| 20240129-0429 | SRDQNILERMLKLLR | STING inactive 6MXE | A1-1000/B1-1000:15-15 | 35.68 | 1.529 | 0.324 | 0.246 | 25.826 |
| 20240127-0042 | SARAERLRQAARDLF | STING inactive 6MXE | A1-1000/B1-1000:15-15 | 30.622 | 1.620 | 0.324 | 0.158 | 26.542 |
| 20240129-0506 | SRLDRILQKLADMMR | STING inactive 6MXE | A1-1000/B1-1000:15-15 | 37.229 | 1.361 | 0.322 | 0.162 | 25.467 |
| 20240129-0505 | SRLDRILSKLAEMMR | STING inactive 6MXE | A1-1000/B1-1000:15-15 | 37.419 | 1.372 | 0.321 | 0.177 | 25.173 |
| 20240127-0720 | SHMAQRLAKVF | STING inactive 6MXE | A1-1000/B1-1000:11-11 | 33.019 | 1.814 | 0.320 | 0.195 | 26.278 |
| 20240127-0055 | DSVLLKSAQALGIPV | STING inactive 6MXE | A1-1000/B1-1000:15-15 | 13.54 | 1.228 | 0.320 | 0.278 | 22.351 |
| 20240127-0041 | SSRRERLVNNARNLF | STING inactive 6MXE | A1-1000/B1-1000:15-15 | 31.022 | 1.583 | 0.320 | 0.144 | 27.193 |
| 20240129-0595 | DSQREALDQILG | STING inactive 6MXE | A1-1000/B1-1000:12-12 | 15.433 | 1.375 | 0.318 | 0.283 | 21.891 |
| 20240129-0509 | SVTFEELLEAVRKAA | STING inactive 6MXE | A1-1000/B1-1000:15-15 | 15.094 | 1.410 | 0.317 | 0.245 | 23.45 |
| 20240127-0063 | SSTVLKAARALGIPV | STING inactive 6MXE | A1-1000/B1-1000:15-15 | 13.642 | 1.258 | 0.317 | 0.265 | 22.897 |
| 20240127-0020 | SEAAEARRERYRKLS | STING inactive 6MXE | A1-1000/B1-1000:15-15 | 26.46 | 1.284 | 0.317 | 0.184 | 24.536 |
| 20240127-0587 | MSLADLVSLMLK | STING inactive 6MXE | A1-1000/B1-1000:12-12 | 47.717 | 1.194 | 0.316 | 0.186 | 26.522 |
| 20240129-0507 | SELDRILSKLAEMMR | STING inactive 6MXE | A1-1000/B1-1000:15-15 | 36.614 | 1.377 | 0.315 | 0.139 | 25.756 |
| 20240129-0484 | PDPARAIAEMLDSLR | STING inactive 6MXE | A1-1000/B1-1000:15-15 | 12.789 | 1.379 | 0.314 | 0.269 | 22.731 |
| 20240127-0718 | SDALERALRVF | STING inactive 6MXE | A1-1000/B1-1000:11-11 | 33.673 | 1.666 | 0.314 | 0.166 | 26.951 |
| 20240127-0710 | SDALERALRVF | STING inactive 6MXE | A1-1000/B1-1000:11-11 | 33.673 | 1.728 | 0.314 | 0.166 | 26.951 |
| 20240129-0603 | SAERLARLKRVW | STING inactive 6MXE | A1-1000/B1-1000:12-12 | 40.231 | 1.443 | 0.313 | 0.23 | 25.728 |
| 20240129-0513 | DLFDKSPKNIAV | STING inactive 6MXE | A1-1000/B1-1000:12-12 | 46.536 | 1.379 | 0.313 | 0.1 | 28.909 |
| 20240129-0481 | SDPTKAIAEMLESLR | STING inactive 6MXE | A1-1000/B1-1000:15-15 | 14.185 | 1.375 | 0.313 | 0.25 | 23.461 |
| 20240127-0038 | SARKKRLVENTRNLF | STING inactive 6MXE | A1-1000/B1-1000:15-15 | 31.92 | 1.584 | 0.313 | 0.155 | 26.599 |
| 20240129-0615 | SKALERAKRMGL | STING inactive 6MXE | A1-1000/B1-1000:12-12 | 35.858 | 1.229 | 0.312 | 0.149 | 26.965 |
| 20240129-0482 | PDPTKAIAEMLESLR | STING inactive 6MXE | A1-1000/B1-1000:15-15 | 14.436 | 1.369 | 0.311 | 0.248 | 23.525 |
| 20240129-0596 | SSQEESLNLILG | STING inactive 6MXE | A1-1000/B1-1000:12-12 | 15.704 | 1.354 | 0.310 | 0.252 | 22.583 |
| 20240127-0708 | SDALEKFLQVS | STING inactive 6MXE | A1-1000/B1-1000:11-11 | 37.554 | 1.706 | 0.310 | 0.161 | 27.238 |
| 20240127-0585 | MDLADLVSALLK | STING inactive 6MXE | A1-1000/B1-1000:12-12 | 14.88 | 1.193 | 0.310 | 0.285 | 22.341 |
| 20240127-0091 | SSSEEHLRGLRQQAR | STING inactive 6MXE | A1-1000/B1-1000:15-15 | 42.044 | 1.507 | 0.310 | 0.15 | 27.298 |
| 20240127-0058 | DSVVLRAARALGIPV | STING inactive 6MXE | A1-1000/B1-1000:15-15 | 13.952 | 1.225 | 0.310 | 0.254 | 23.245 |
| 20240129-0386 | SSLDKLLLKTYEMFK | STING inactive 6MXE | A1-1000/B1-1000:15-15 | 30.4 | 1.341 | 0.309 | 0.165 | 25.978 |
| 20240127-0049 | DSVLLKSARALGIPV | STING inactive 6MXE | A1-1000/B1-1000:15-15 | 14.842 | 1.249 | 0.308 | 0.233 | 23.36 |
| 20240129-0528 | SKFREFLKSLAA | STING inactive 6MXE | A1-1000/B1-1000:12-12 | 44.181 | 1.876 | 0.306 | 0.184 | 26.373 |
| 20240129-0483 | SDPAKAIAEMMESLR | STING inactive 6MXE | A1-1000/B1-1000:15-15 | 13.555 | 1.399 | 0.306 | 0.25 | 23.538 |
| 20240129-0400 | ASELAQRREAMQESF | STING inactive 6MXE | A1-1000/B1-1000:15-15 | 28.967 | 1.720 | 0.306 | 0.105 | 27.069 |
| 20240129-0508 | DRLRRILQKLADMMR | STING inactive 6MXE | A1-1000/B1-1000:15-15 | 39.907 | 1.320 | 0.305 | 0.155 | 26.215 |
| 20240127-0143 | GSLSVFEKMMERIRS | STING inactive 6MXE | A1-1000/B1-1000:15-15 | 37.568 | 1.495 | 0.305 | 0.132 | 26.513 |
| 20240129-0387 | SARDKLLLRTYEMFL | STING inactive 6MXE | A1-1000/B1-1000:15-15 | 4.929 | 1.381 | 0.304 | 0.247 | 22.355 |
| 20240127-0537 | MMSAQEKLAAIM | STING inactive 6MXE | A1-1000/B1-1000:12-12 | 15.979 | 1.294 | 0.304 | 0.205 | 23.789 |
| 20240129-0616 | SKELERARRMGL | STING inactive 6MXE | A1-1000/B1-1000:12-12 | 31.89 | 1.194 | 0.303 | 0.14 | 26.771 |
| 20240127-0140 | GSLSVFEKLQEMIRS | STING inactive 6MXE | A1-1000/B1-1000:15-15 | 40.4 | 1.531 | 0.303 | 0.155 | 26.398 |
| 20240129-0604 | SSERLARLRAVW | STING inactive 6MXE | A1-1000/B1-1000:12-12 | 40.637 | 1.493 | 0.302 | 0.216 | 25.934 |
| 20240127-0717 | SDTLEKFLRVS | STING inactive 6MXE | A1-1000/B1-1000:11-11 | 38.48 | 1.678 | 0.301 | 0.187 | 26.838 |
| 20240129-0623 | SKASQRLFEIWR | STING inactive 6MXE | A1-1000/B1-1000:12-12 | 38.101 | 1.589 | 0.300 | 0.155 | 26.823 |
| 20240129-0622 | SKASQRLFEIWR | STING inactive 6MXE | A1-1000/B1-1000:12-12 | 38.101 | 1.616 | 0.300 | 0.155 | 26.823 |
| 20240129-0385 | SSRDKILLKVYELFK | STING inactive 6MXE | A1-1000/B1-1000:15-15 | 30.501 | 1.349 | 0.300 | 0.156 | 26.454 |
| 20240127-0541 | MESAQDKLAAIM | STING inactive 6MXE | A1-1000/B1-1000:12-12 | 15.781 | 1.247 | 0.300 | 0.224 | 23.314 |
| 20240129-0441 | VDIERLARTLGTAKL | STING inactive 6MXE | A1-1000/B1-1000:15-15 | 15.349 | 1.160 | 0.299 | 0.263 | 22.538 |
| 20240129-0424 | SIAERMRRLGFRLTD | STING inactive 6MXE | A1-1000/B1-1000:15-15 | 27.212 | 1.169 | 0.299 | 0.139 | 25.865 |
| 20240127-0034 | SARRERMVNATKNLF | STING inactive 6MXE | A1-1000/B1-1000:15-15 | 30.934 | 1.612 | 0.299 | 0.158 | 26.588 |
| 20240129-0510 | SVTFEERMEAVRKAA | STING inactive 6MXE | A1-1000/B1-1000:15-15 | 33.746 | 1.432 | 0.298 | 0.123 | 26.727 |
| 20240129-0404 | MSASERHLKLIRDVW | STING inactive 6MXE | A1-1000/B1-1000:15-15 | 33.035 | 1.287 | 0.298 | 0.184 | 25.579 |
| 20240129-0614 | SRALERAKRMGL | STING inactive 6MXE | A1-1000/B1-1000:12-12 | 34.803 | 1.238 | 0.296 | 0.143 | 27.042 |
| 20240129-0467 | SAADARERYLESLLR | STING inactive 6MXE | A1-1000/B1-1000:15-15 | 16.641 | 1.544 | 0.296 | 0.211 | 24.024 |
| 20240127-0057 | SSVVLRSARALGIPV | STING inactive 6MXE | A1-1000/B1-1000:15-15 | 13.24 | 1.210 | 0.296 | 0.249 | 23.05 |
| 20240129-0720 | GPLLLRFIERL | STING inactive 6MXE | A1-1000/B1-1000:11-11 | 18.678 | 1.258 | 0.295 | 0.234 | 23.582 |
| 20240129-0401 | MSASERHAQLIRDVW | STING inactive 6MXE | A1-1000/B1-1000:15-15 | 33.066 | 1.351 | 0.295 | 0.175 | 25.733 |
| 20240127-0138 | GSLSVAERMMERIRR | STING inactive 6MXE | A1-1000/B1-1000:15-15 | 33.41 | 1.536 | 0.295 | 0.126 | 26.218 |
| 20240127-0052 | SSVLLRAARALGIPL | STING inactive 6MXE | A1-1000/B1-1000:15-15 | 13.949 | 1.210 | 0.295 | 0.236 | 23.836 |
| 20240127-0060 | DSVVRRAAQALGIPV | STING inactive 6MXE | A1-1000/B1-1000:15-15 | 33.048 | 1.260 | 0.294 | 0.159 | 25.788 |
| 20240129-0601 | SPERLARLKRVW | STING inactive 6MXE | A1-1000/B1-1000:12-12 | 39.678 | 1.417 | 0.293 | 0.215 | 25.788 |
| 20240129-0402 | MSAEERHLKLIREVI | STING inactive 6MXE | A1-1000/B1-1000:15-15 | 18.819 | 1.369 | 0.293 | 0.183 | 24.648 |
| 20240127-0713 | SNTLERALRVF | STING inactive 6MXE | A1-1000/B1-1000:11-11 | 33.462 | 1.713 | 0.293 | 0.168 | 26.801 |
| 20240129-0487 | SPEQLRRTLRLMHPV | STING inactive 6MXE | A1-1000/B1-1000:15-15 | 12.525 | 1.550 | 0.292 | 0.237 | 23.389 |
| 20240129-0408 | SPAEKAVARMRRLFS | STING inactive 6MXE | A1-1000/B1-1000:15-15 | 32.588 | 1.405 | 0.292 | 0.163 | 25.688 |
| 20240127-0064 | SSVMLRAARALGIPL | STING inactive 6MXE | A1-1000/B1-1000:15-15 | 14.156 | 1.228 | 0.292 | 0.225 | 24.029 |
| 20240129-0433 | SMRRAEDVRRLFEGM | STING inactive 6MXE | A1-1000/B1-1000:15-15 | 19.941 | 1.475 | 0.291 | 0.234 | 23.507 |
| 20240127-0707 | SNALERALKVF | STING inactive 6MXE | A1-1000/B1-1000:11-11 | 33.066 | 1.707 | 0.291 | 0.174 | 26.531 |
| 20240127-0022 | SEASEARRERYLKLS | STING inactive 6MXE | A1-1000/B1-1000:15-15 | 24.866 | 1.334 | 0.291 | 0.156 | 24.692 |
| 20240129-0593 | SSQQESLQQILG | STING inactive 6MXE | A1-1000/B1-1000:12-12 | 15.884 | 1.358 | 0.290 | 0.223 | 23.307 |
| 20240129-0585 | LSRVIEMWKSRL | STING inactive 6MXE | A1-1000/B1-1000:12-12 | 38.386 | 1.386 | 0.290 | 0.181 | 26.153 |
| 20240129-0559 | DALTQQLLASLA | STING inactive 6MXE | A1-1000/B1-1000:12-12 | 48.453 | 1.683 | 0.290 | 0.201 | 26.195 |
| 20240127-0130 | GSLSVFQRMMERMRA | STING inactive 6MXE | A1-1000/B1-1000:15-15 | 37.133 | 1.498 | 0.290 | 0.129 | 26.806 |
| 20240129-0561 | SSVLELLRSFLE | STING inactive 6MXE | A1-1000/B1-1000:12-12 | 44.891 | 1.388 | 0.289 | 0.169 | 26.268 |
| 20240129-0388 | SSRDKLLLKVYDMFK | STING inactive 6MXE | A1-1000/B1-1000:15-15 | 30.278 | 1.308 | 0.289 | 0.169 | 25.839 |
| 20240127-0591 | MSPADLVSAMLK | STING inactive 6MXE | A1-1000/B1-1000:12-12 | 45.245 | 1.221 | 0.289 | 0.212 | 25.833 |
| 20240127-0583 | MSPADLVSAMLK | STING inactive 6MXE | A1-1000/B1-1000:12-12 | 45.245 | 1.182 | 0.289 | 0.212 | 25.833 |
| 20240127-0134 | GSLSVFQKMMERIRS | STING inactive 6MXE | A1-1000/B1-1000:15-15 | 36.846 | 1.502 | 0.289 | 0.122 | 26.646 |
| 20240127-0037 | SRRRQRLVENQRNLS | STING inactive 6MXE | A1-1000/B1-1000:15-15 | 34.147 | 1.593 | 0.289 | 0.178 | 25.973 |
| 20240127-0026 | SEASKARAERYSKLS | STING inactive 6MXE | A1-1000/B1-1000:15-15 | 25.902 | 1.391 | 0.289 | 0.169 | 24.671 |
| 20240129-0749 | GPEIILRRMEE | STING inactive 6MXE | A1-1000/B1-1000:11-11 | 12.819 | 1.499 | 0.288 | 0.226 | 23.183 |
| 20240129-0676 | GDAALLANMLS | STING inactive 6MXE | A1-1000/B1-1000:11-11 | 13.779 | 1.628 | 0.288 | 0.257 | 22.414 |
| 20240129-0656 | TAWMERLGVRV | STING inactive 6MXE | A1-1000/B1-1000:11-11 | 34.227 | 1.094 | 0.288 | 0.202 | 26.264 |
| 20240127-0029 | SEASQARLERYRRLS | STING inactive 6MXE | A1-1000/B1-1000:15-15 | 24.649 | 1.383 | 0.288 | 0.171 | 24.38 |
| 20240129-0624 | SKAFQKLFEIWR | STING inactive 6MXE | A1-1000/B1-1000:12-12 | 37.686 | 1.642 | 0.287 | 0.19 | 26.106 |
| 20240129-0609 | LSIWESVEEMLK | STING inactive 6MXE | A1-1000/B1-1000:12-12 | 37.363 | 1.738 | 0.287 | 0.152 | 25.652 |
| 20240127-0059 | DKVVLLSARALGIKV | STING inactive 6MXE | A1-1000/B1-1000:15-15 | 35.913 | 1.278 | 0.287 | 0.176 | 25.79 |
| 20240127-0139 | MSRKVAERMMEMIRS | STING inactive 6MXE | A1-1000/B1-1000:15-15 | 34.207 | 1.496 | 0.286 | 0.146 | 26.095 |
| 20240127-0083 | SSAERHLEGMRKQSE | STING inactive 6MXE | A1-1000/B1-1000:15-15 | 40.023 | 1.540 | 0.286 | 0.174 | 26.643 |
| 20240129-0522 | MREKMLKTAKII | STING inactive 6MXE | A1-1000/B1-1000:12-12 | 31.338 | 1.428 | 0.285 | 0.154 | 26.756 |
| 20240129-0448 | SLALKAAIEINKLTS | STING inactive 6MXE | A1-1000/B1-1000:15-15 | 18.367 | 1.387 | 0.285 | 0.226 | 23.841 |
| 20240129-0422 | SIEERMRRLGFRLTQ | STING inactive 6MXE | A1-1000/B1-1000:15-15 | 18.287 | 1.147 | 0.285 | 0.16 | 25.541 |
| 20240127-0131 | GSLSVAQRWWDMLRS | STING inactive 6MXE | A1-1000/B1-1000:15-15 | 35.676 | 1.551 | 0.285 | 0.153 | 26.01 |
| 20240129-0757 | SVWERHMRATT | STING inactive 6MXE | A1-1000/B1-1000:11-11 | 35.548 | 1.317 | 0.284 | 0.2 | 26.006 |
| 20240129-0620 | KWLEAMKAKMKL | STING inactive 6MXE | A1-1000/B1-1000:12-12 | 19.603 | 1.500 | 0.284 | 0.139 | 25.615 |
| 20240129-0558 | SAATEELLRRLA | STING inactive 6MXE | A1-1000/B1-1000:12-12 | 47.079 | 1.512 | 0.284 | 0.152 | 26.677 |
| 20240129-0504 | SAPSIAEQADLYGSL | STING inactive 6MXE | A1-1000/B1-1000:15-15 | 16.297 | 1.181 | 0.284 | 0.21 | 23.471 |
| 20240129-0456 | SEEEEQRIARARRIT | STING inactive 6MXE | A1-1000/B1-1000:15-15 | 28.853 | 1.556 | 0.284 | 0.112 | 26.889 |
| 20240127-0039 | SREREKRVNAMRNLS | STING inactive 6MXE | A1-1000/B1-1000:15-15 | 28.257 | 1.650 | 0.284 | 0.109 | 27.492 |
| 20240127-0025 | SEASEARREKYRKLS | STING inactive 6MXE | A1-1000/B1-1000:15-15 | 28.247 | 1.351 | 0.284 | 0.123 | 26.067 |
| 20240129-0752 | GPQLILDRMES | STING inactive 6MXE | A1-1000/B1-1000:11-11 | 12.857 | 1.502 | 0.283 | 0.237 | 23.018 |
| 20240129-0566 | SFLEQLREARPE | STING inactive 6MXE | A1-1000/B1-1000:12-12 | 47.667 | 1.454 | 0.283 | 0.163 | 27.409 |
| 20240129-0470 | SEQLMGEFMKRAMRG | STING inactive 6MXE | A1-1000/B1-1000:15-15 | 15.715 | 1.293 | 0.283 | 0.198 | 24.584 |
| 20240127-0137 | GSRSVFEKMMERIRS | STING inactive 6MXE | A1-1000/B1-1000:15-15 | 34.514 | 1.530 | 0.283 | 0.128 | 26.48 |
| 20240129-0618 | KWLESMRSRLKL | STING inactive 6MXE | A1-1000/B1-1000:12-12 | 25.405 | 1.524 | 0.282 | 0.158 | 26.066 |
| 20240129-0492 | SMAEILLRAMKVIAG | STING inactive 6MXE | A1-1000/B1-1000:15-15 | 37.537 | 1.253 | 0.282 | 0.175 | 26.104 |
| 20240129-0468 | SNRDALERYRKTLLA | STING inactive 6MXE | A1-1000/B1-1000:15-15 | 29.211 | 1.409 | 0.282 | 0.168 | 25.88 |
| 20240129-0466 | SARDALERYRKTLLA | STING inactive 6MXE | A1-1000/B1-1000:15-15 | 29.394 | 1.425 | 0.282 | 0.166 | 25.6 |
| 20240127-0706 | SDALERFLKVS | STING inactive 6MXE | A1-1000/B1-1000:11-11 | 33.043 | 1.641 | 0.282 | 0.164 | 26.763 |
| 20240127-0074 | MSTPKILARLESLRE | STING inactive 6MXE | A1-1000/B1-1000:15-15 | 38.825 | 1.462 | 0.282 | 0.14 | 27.506 |
| 20240129-0608 | SVLLRAMEKMFG | STING inactive 6MXE | A1-1000/B1-1000:12-12 | 36.601 | 1.191 | 0.281 | 0.159 | 26.247 |
| 20240127-0045 | SRRAERLRRAQRELS | STING inactive 6MXE | A1-1000/B1-1000:15-15 | 32.66 | 1.684 | 0.281 | 0.095 | 27.002 |
| 20240129-0750 | GPQILLDRMES | STING inactive 6MXE | A1-1000/B1-1000:11-11 | 14.467 | 1.456 | 0.280 | 0.215 | 23.799 |
| 20240129-0694 | PVLELSRSLMQ | STING inactive 6MXE | A1-1000/B1-1000:11-11 | 29.749 | 1.595 | 0.280 | 0.231 | 25.496 |
| 20240129-0594 | DSQKEAQDLILG | STING inactive 6MXE | A1-1000/B1-1000:12-12 | 14.994 | 1.440 | 0.280 | 0.223 | 23.033 |
| 20240127-0142 | MSRSVAEKLMERLRS | STING inactive 6MXE | A1-1000/B1-1000:15-15 | 33.58 | 1.538 | 0.280 | 0.131 | 26.306 |
| 20240129-0730 | STVLRETLINL | STING inactive 6MXE | A1-1000/B1-1000:11-11 | 24.353 | 1.349 | 0.279 | 0.232 | 22.867 |
| 20240129-0719 | GPLLLREIERW | STING inactive 6MXE | A1-1000/B1-1000:11-11 | 34.398 | 1.270 | 0.279 | 0.183 | 26.322 |
| 20240129-0431 | SRDKLILERMSRLMS | STING inactive 6MXE | A1-1000/B1-1000:15-15 | 13.385 | 1.543 | 0.279 | 0.204 | 24.578 |
| 20240127-0056 | SSVVLRAARALGIPV | STING inactive 6MXE | A1-1000/B1-1000:15-15 | 32.033 | 1.235 | 0.279 | 0.134 | 26.067 |
| 20240129-0677 | MELALEAALSL | STING inactive 6MXE | A1-1000/B1-1000:11-11 | 13.65 | 1.647 | 0.278 | 0.247 | 23.129 |
| 20240129-0588 | LSRVIEMWRSRL | STING inactive 6MXE | A1-1000/B1-1000:12-12 | 37.508 | 1.275 | 0.278 | 0.174 | 26.307 |
| 20240127-0480 | SSNPLMQMFLNR | STING inactive 6MXE | A1-1000/B1-1000:12-12 | 16.505 | 1.300 | 0.278 | 0.242 | 23.577 |
| 20240127-0466 | SSNPLMQMFLNR | STING inactive 6MXE | A1-1000/B1-1000:12-12 | 16.505 | 1.240 | 0.278 | 0.242 | 23.577 |
| 20240127-0132 | GSLSVFQRMWDRIKS | STING inactive 6MXE | A1-1000/B1-1000:15-15 | 38.045 | 1.481 | 0.278 | 0.154 | 26.29 |
| 20240127-0053 | SSVILKGAQALGIKV | STING inactive 6MXE | A1-1000/B1-1000:15-15 | 32.403 | 1.282 | 0.278 | 0.164 | 25.944 |
| 20240127-0048 | SSRRERMVNNTRNLF | STING inactive 6MXE | A1-1000/B1-1000:15-15 | 34.388 | 1.568 | 0.278 | 0.128 | 27.072 |
| 20240127-0019 | SEAAEARRKRYAKLS | STING inactive 6MXE | A1-1000/B1-1000:15-15 | 27.536 | 1.366 | 0.278 | 0.126 | 25.784 |
| 20240129-0637 | GAVDMARFLLAS | STING inactive 6MXE | A1-1000/B1-1000:12-12 | 39.697 | 1.198 | 0.277 | 0.177 | 26.571 |
| 20240127-0715 | SNALEKLLRVF | STING inactive 6MXE | A1-1000/B1-1000:11-11 | 32.912 | 1.762 | 0.277 | 0.178 | 26.512 |
| 20240127-0129 | MSLSVAQRMMEMIRA | STING inactive 6MXE | A1-1000/B1-1000:15-15 | 36.865 | 1.526 | 0.277 | 0.151 | 26.088 |
| 20240127-0032 | SEASRAREERYRKLS | STING inactive 6MXE | A1-1000/B1-1000:15-15 | 30.68 | 1.302 | 0.277 | 0.111 | 26.234 |
| 20240127-0017 | SEASRAREERYRKLS | STING inactive 6MXE | A1-1000/B1-1000:15-15 | 30.68 | 1.318 | 0.277 | 0.111 | 26.234 |
| 20240129-0415 | MSQQSLKMYEKIFKS | STING inactive 6MXE | A1-1000/B1-1000:15-15 | 27.629 | 1.321 | 0.276 | 0.138 | 26.231 |
| 20240127-0027 | SEASKAREERYRKLS | STING inactive 6MXE | A1-1000/B1-1000:15-15 | 33.865 | 1.278 | 0.276 | 0.103 | 26.616 |
| 20240127-0133 | MSRKVADKMMEKIRS | STING inactive 6MXE | A1-1000/B1-1000:15-15 | 31.101 | 1.541 | 0.275 | 0.132 | 26.067 |
| 20240127-0085 | SSALEHLKGMLKQSR | STING inactive 6MXE | A1-1000/B1-1000:15-15 | 38.57 | 1.582 | 0.275 | 0.17 | 26.095 |
| 20240127-0036 | DRRRRRMVENARNLF | STING inactive 6MXE | A1-1000/B1-1000:15-15 | 20.686 | 1.563 | 0.275 | 0.129 | 26.209 |
| 20240127-0030 | SEASRARAERFRRLS | STING inactive 6MXE | A1-1000/B1-1000:15-15 | 24.616 | 1.355 | 0.275 | 0.159 | 24.867 |
| 20240129-0565 | SFLRALREARPE | STING inactive 6MXE | A1-1000/B1-1000:12-12 | 46.264 | 1.461 | 0.274 | 0.165 | 27.281 |
| 20240129-0454 | SAAEAARIEEARRRT | STING inactive 6MXE | A1-1000/B1-1000:15-15 | 29.94 | 1.620 | 0.274 | 0.13 | 27.461 |
| 20240129-0453 | SAAEEERIRRARRIT | STING inactive 6MXE | A1-1000/B1-1000:15-15 | 30.234 | 1.534 | 0.274 | 0.119 | 26.766 |
| 20240127-0592 | MSPADLVSALLK | STING inactive 6MXE | A1-1000/B1-1000:12-12 | 43.685 | 1.171 | 0.274 | 0.189 | 26.047 |
| 20240127-0580 | MSPADLVSALLK | STING inactive 6MXE | A1-1000/B1-1000:12-12 | 43.685 | 1.144 | 0.274 | 0.189 | 26.047 |
| 20240129-0675 | GPASLLANELS | STING inactive 6MXE | A1-1000/B1-1000:11-11 | 13.213 | 1.650 | 0.273 | 0.23 | 23.172 |
| 20240129-0434 | SMRRAEAVRSLFEGM | STING inactive 6MXE | A1-1000/B1-1000:15-15 | 21.075 | 1.513 | 0.273 | 0.193 | 24.8 |
| 20240127-0503 | SLLEELSRLARL | STING inactive 6MXE | A1-1000/B1-1000:12-12 | 47.958 | 1.406 | 0.273 | 0.165 | 25.714 |
| 20240129-0455 | SEAVRERIERARRIT | STING inactive 6MXE | A1-1000/B1-1000:15-15 | 27.836 | 1.529 | 0.272 | 0.124 | 26.273 |
| 20240129-0440 | SRLEELLRKTPKYLA | STING inactive 6MXE | A1-1000/B1-1000:15-15 | 39.332 | 1.613 | 0.272 | 0.179 | 26.122 |
| 20240129-0753 | RRLLLRAYEVV | STING inactive 6MXE | A1-1000/B1-1000:11-11 | 43.989 | 1.337 | 0.271 | 0.163 | 27.395 |
| 20240129-0496 | MSSMTEFERGKRMLK | STING inactive 6MXE | A1-1000/B1-1000:15-15 | 21.413 | 1.600 | 0.271 | 0.142 | 26.132 |
| 20240129-0399 | SSFLSKKREEMQELP | STING inactive 6MXE | A1-1000/B1-1000:15-15 | 25.033 | 1.709 | 0.271 | 0.121 | 26.921 |
| 20240127-0498 | SLLEQLSKLARL | STING inactive 6MXE | A1-1000/B1-1000:12-12 | 47.59 | 1.457 | 0.271 | 0.157 | 26.055 |
| 20240127-0078 | MKTPHILKRLEELRS | STING inactive 6MXE | A1-1000/B1-1000:15-15 | 33.717 | 1.315 | 0.271 | 0.127 | 27.122 |
| 20240129-0626 | SVLRKFGEFMLG | STING inactive 6MXE | A1-1000/B1-1000:12-12 | 35.74 | 1.261 | 0.270 | 0.169 | 25.944 |
| 20240129-0430 | SSDANRLARMQRLFA | STING inactive 6MXE | A1-1000/B1-1000:15-15 | 29.95 | 1.535 | 0.270 | 0.153 | 26.94 |
| 20240127-0144 | GSLSVFQKMMDRIRS | STING inactive 6MXE | A1-1000/B1-1000:15-15 | 36.131 | 1.514 | 0.270 | 0.125 | 26.631 |
| 20240127-0141 | GSLSVAQRWMDRIRS | STING inactive 6MXE | A1-1000/B1-1000:15-15 | 31.779 | 1.480 | 0.270 | 0.147 | 25.939 |
| 20240127-0035 | SRRAERMRQAQLDLS | STING inactive 6MXE | A1-1000/B1-1000:15-15 | 35.075 | 1.632 | 0.270 | 0.108 | 27.004 |
| 20240129-0742 | MELIEMWRSVL | STING inactive 6MXE | A1-1000/B1-1000:11-11 | 32.803 | 1.323 | 0.269 | 0.183 | 26.243 |
| 20240129-0621 | SKASQRWFEILK | STING inactive 6MXE | A1-1000/B1-1000:12-12 | 33.259 | 1.584 | 0.269 | 0.157 | 26.12 |
| 20240127-0075 | MKTPKILEALERLRE | STING inactive 6MXE | A1-1000/B1-1000:15-15 | 31.244 | 1.377 | 0.269 | 0.144 | 26.37 |
| 20240127-0068 | MKTPKILAALERLRE | STING inactive 6MXE | A1-1000/B1-1000:15-15 | 35.825 | 1.440 | 0.269 | 0.137 | 27.034 |
| 20240129-0619 | KWLEAMRSRMKL | STING inactive 6MXE | A1-1000/B1-1000:12-12 | 22.821 | 1.475 | 0.268 | 0.156 | 25.87 |
| 20240127-0590 | MSPADLVSLLLK | STING inactive 6MXE | A1-1000/B1-1000:12-12 | 42.988 | 1.166 | 0.268 | 0.191 | 25.889 |
| 20240127-0507 | SYLERLSQLARL | STING inactive 6MXE | A1-1000/B1-1000:12-12 | 46.959 | 1.419 | 0.268 | 0.156 | 26.15 |
| 20240129-0759 | SVWERHMRVVT | STING inactive 6MXE | A1-1000/B1-1000:11-11 | 36.636 | 1.289 | 0.267 | 0.2 | 25.953 |
| 20240127-0033 | SARAERLREAQRSLS | STING inactive 6MXE | A1-1000/B1-1000:15-15 | 32.669 | 1.656 | 0.267 | 0.146 | 26.315 |
| 20240129-0728 | SLLMKVIEMNR | STING inactive 6MXE | A1-1000/B1-1000:11-11 | 16.611 | 1.350 | 0.266 | 0.187 | 24.637 |
| 20240129-0718 | GPLLLRWIERL | STING inactive 6MXE | A1-1000/B1-1000:11-11 | 30.904 | 1.251 | 0.266 | 0.159 | 26.708 |
| 20240129-0617 | RWMEAMRARMKL | STING inactive 6MXE | A1-1000/B1-1000:12-12 | 17.118 | 1.502 | 0.266 | 0.135 | 25.973 |
| 20240129-0471 | SERLMGEFMRRAMRG | STING inactive 6MXE | A1-1000/B1-1000:15-15 | 15.054 | 1.218 | 0.266 | 0.202 | 24.48 |
| 20240129-0392 | SASAQARLRKILDGL | STING inactive 6MXE | A1-1000/B1-1000:15-15 | 20.968 | 1.526 | 0.266 | 0.128 | 25.937 |
| 20240129-0390 | SASTQERLRRIMSGL | STING inactive 6MXE | A1-1000/B1-1000:15-15 | 31.393 | 1.543 | 0.266 | 0.155 | 26.238 |
| 20240127-0135 | MSLSVAQRMMEAIRS | STING inactive 6MXE | A1-1000/B1-1000:15-15 | 35.705 | 1.560 | 0.266 | 0.144 | 26.214 |
| 20240127-0101 | GPSKLLQAYDTLTAR | STING inactive 6MXE | A1-1000/B1-1000:15-15 | 26.963 | 1.284 | 0.266 | 0.162 | 25.815 |
| 20240129-0560 | DALVEELLRRLA | STING inactive 6MXE | A1-1000/B1-1000:12-12 | 45.699 | 1.539 | 0.265 | 0.147 | 26.174 |
| 20240129-0428 | SRVQARLERMMRGVL | STING inactive 6MXE | A1-1000/B1-1000:15-15 | 18.826 | 1.349 | 0.265 | 0.138 | 25.863 |
| 20240129-0427 | SRVQQRLLDMMRGVL | STING inactive 6MXE | A1-1000/B1-1000:15-15 | 17.936 | 1.333 | 0.265 | 0.159 | 25.677 |
| 20240129-0425 | SRVQARLERMMRGVL | STING inactive 6MXE | A1-1000/B1-1000:15-15 | 18.826 | 1.304 | 0.265 | 0.138 | 25.863 |
| 20240129-0405 | SPAERAVARFRRLAS | STING inactive 6MXE | A1-1000/B1-1000:15-15 | 26.928 | 1.308 | 0.265 | 0.138 | 26.043 |
| 20240127-0467 | SSNPLMQMFENR | STING inactive 6MXE | A1-1000/B1-1000:12-12 | 18.034 | 1.206 | 0.265 | 0.218 | 24.024 |
| 20240127-0136 | MSRSVAERMMEKIRS | STING inactive 6MXE | A1-1000/B1-1000:15-15 | 29.598 | 1.530 | 0.265 | 0.12 | 26.347 |
| 20240127-0065 | MKTPKILRMLERLRE | STING inactive 6MXE | A1-1000/B1-1000:15-15 | 27.972 | 1.460 | 0.265 | 0.156 | 25.881 |
| 20240127-0061 | SSVVLKSARALGIKV | STING inactive 6MXE | A1-1000/B1-1000:15-15 | 31.01 | 1.241 | 0.265 | 0.138 | 26.198 |
| 20240127-0043 | SSAAERRRNAMLNLS | STING inactive 6MXE | A1-1000/B1-1000:15-15 | 36.457 | 1.646 | 0.265 | 0.115 | 27.232 |
| 20240129-0758 | SVWDRHMRQIT | STING inactive 6MXE | A1-1000/B1-1000:11-11 | 37.35 | 1.420 | 0.264 | 0.175 | 26.768 |
| 20240129-0602 | SPERLERLKRVI | STING inactive 6MXE | A1-1000/B1-1000:12-12 | 25.465 | 1.448 | 0.264 | 0.14 | 26.029 |
| 20240129-0574 | SSAFMERLRATR | STING inactive 6MXE | A1-1000/B1-1000:12-12 | 22.54 | 1.252 | 0.264 | 0.26 | 23.801 |
| 20240129-0486 | SAEQRRRARRMMRPV | STING inactive 6MXE | A1-1000/B1-1000:15-15 | 33.71 | 1.607 | 0.264 | 0.199 | 25.383 |
| 20240129-0426 | SRVQERLRRMMRGVL | STING inactive 6MXE | A1-1000/B1-1000:15-15 | 25.535 | 1.382 | 0.264 | 0.13 | 26.084 |
| 20240129-0393 | SEVNEKVLRKAAELN | STING inactive 6MXE | A1-1000/B1-1000:15-15 | 36.192 | 1.326 | 0.264 | 0.154 | 26.025 |
| 20240127-0634 | SAKVSTYETLS | STING inactive 6MXE | A1-1000/B1-1000:11-11 | 42.089 | 1.356 | 0.264 | 0.099 | 28.715 |
| 20240127-0511 | SLLEELSKMVRL | STING inactive 6MXE | A1-1000/B1-1000:12-12 | 48.018 | 1.488 | 0.264 | 0.158 | 25.911 |
| 20240127-0102 | KPSKLLESYERLTAR | STING inactive 6MXE | A1-1000/B1-1000:15-15 | 9.979 | 1.288 | 0.264 | 0.18 | 24.543 |
| 20240127-0092 | SSMERHLKGLLKQSK | STING inactive 6MXE | A1-1000/B1-1000:15-15 | 36.77 | 1.585 | 0.264 | 0.146 | 26.438 |
| 20240129-0465 | SAQDALDRYRKTLLA | STING inactive 6MXE | A1-1000/B1-1000:15-15 | 28.504 | 1.451 | 0.263 | 0.142 | 25.891 |
| 20240129-0432 | SRDANILARMRKLLS | STING inactive 6MXE | A1-1000/B1-1000:15-15 | 27.46 | 1.498 | 0.263 | 0.155 | 26.179 |
| 20240129-0391 | SASAQARLERILRGL | STING inactive 6MXE | A1-1000/B1-1000:15-15 | 24.071 | 1.502 | 0.263 | 0.154 | 25.326 |
| 20240127-0080 | MKTPKILEALERLRA | STING inactive 6MXE | A1-1000/B1-1000:15-15 | 32.979 | 1.407 | 0.263 | 0.142 | 26.463 |
| 20240127-0069 | MKTPKILEALERLRA | STING inactive 6MXE | A1-1000/B1-1000:15-15 | 32.979 | 1.424 | 0.263 | 0.142 | 26.463 |
| 20240129-0610 | LSIWSSVEEMRK | STING inactive 6MXE | A1-1000/B1-1000:12-12 | 39.98 | 1.740 | 0.262 | 0.142 | 26.511 |
| 20240129-0607 | SVLLRAMEVMFG | STING inactive 6MXE | A1-1000/B1-1000:12-12 | 35.36 | 1.188 | 0.262 | 0.16 | 26.058 |
| 20240129-0512 | MVTNVERMEAVRKAA | STING inactive 6MXE | A1-1000/B1-1000:15-15 | 20.709 | 1.428 | 0.262 | 0.118 | 26.584 |
| 20240129-0438 | SSLWEALKKIPSYLR | STING inactive 6MXE | A1-1000/B1-1000:15-15 | 36.669 | 1.605 | 0.262 | 0.136 | 26.076 |
| 20240127-0021 | SSASEARRERYRKLS | STING inactive 6MXE | A1-1000/B1-1000:15-15 | 28.448 | 1.363 | 0.262 | 0.136 | 25.556 |
| 20240129-0744 | MKLIEMWKSVL | STING inactive 6MXE | A1-1000/B1-1000:11-11 | 32.856 | 1.275 | 0.261 | 0.206 | 25.708 |
| 20240129-0490 | SMRDILLRAMRVISG | STING inactive 6MXE | A1-1000/B1-1000:15-15 | 34.949 | 1.307 | 0.261 | 0.153 | 25.911 |
| 20240127-0584 | MDPASLVSAMLK | STING inactive 6MXE | A1-1000/B1-1000:12-12 | 43.351 | 1.156 | 0.261 | 0.189 | 25.948 |
| 20240127-0579 | MDPASLVSAMLK | STING inactive 6MXE | A1-1000/B1-1000:12-12 | 43.351 | 1.210 | 0.261 | 0.189 | 25.948 |
| 20240127-0044 | SRRAERLRENMRNLS | STING inactive 6MXE | A1-1000/B1-1000:15-15 | 24.129 | 1.626 | 0.261 | 0.122 | 26.22 |
| 20240129-0606 | SVSLEAIRKMFG | STING inactive 6MXE | A1-1000/B1-1000:12-12 | 33.516 | 1.230 | 0.260 | 0.146 | 26.177 |
| 20240129-0511 | RVTFEERLRAVRAAS | STING inactive 6MXE | A1-1000/B1-1000:15-15 | 31.037 | 1.447 | 0.260 | 0.119 | 26.585 |
| 20240129-0444 | VDLERLARQLGTAKL | STING inactive 6MXE | A1-1000/B1-1000:15-15 | 14.963 | 1.098 | 0.260 | 0.178 | 25.31 |
| 20240127-0483 | MAVVEMNRLLLS | STING inactive 6MXE | A1-1000/B1-1000:12-12 | 16.269 | 1.395 | 0.260 | 0.224 | 23.455 |
| 20240129-0613 | SAALKRAKRMGL | STING inactive 6MXE | A1-1000/B1-1000:12-12 | 23 | 1.266 | 0.259 | 0.132 | 26.138 |
| 20240129-0591 | GPLRSVAEMLRA | STING inactive 6MXE | A1-1000/B1-1000:12-12 | 37.165 | 1.002 | 0.259 | 0.191 | 26.135 |
| 20240129-0502 | SAPSILDRADLLGSL | STING inactive 6MXE | A1-1000/B1-1000:15-15 | 15.718 | 1.255 | 0.259 | 0.232 | 23.114 |
| 20240129-0437 | SDLEELLRKLPQYVA | STING inactive 6MXE | A1-1000/B1-1000:15-15 | 28.555 | 1.606 | 0.259 | 0.126 | 26.702 |
| 20240129-0413 | MSKQSLLMYEKIFKS | STING inactive 6MXE | A1-1000/B1-1000:15-15 | 28.461 | 1.325 | 0.259 | 0.14 | 26.295 |
| 20240127-0712 | SSSRQRLLEVF | STING inactive 6MXE | A1-1000/B1-1000:11-11 | 30.513 | 1.734 | 0.259 | 0.144 | 26.519 |
| 20240127-0079 | MKVPKILEALESLRE | STING inactive 6MXE | A1-1000/B1-1000:15-15 | 33.342 | 1.438 | 0.259 | 0.145 | 26.661 |
| 20240129-0743 | MKLIEMWRSVL | STING inactive 6MXE | A1-1000/B1-1000:11-11 | 33.465 | 1.379 | 0.258 | 0.209 | 25.714 |
| 20240129-0489 | SMAELLLRSMKVISG | STING inactive 6MXE | A1-1000/B1-1000:15-15 | 20.709 | 1.203 | 0.258 | 0.153 | 25.809 |
| 20240129-0450 | DSLRRLLLRSYETVA | STING inactive 6MXE | A1-1000/B1-1000:15-15 | 37.921 | 1.320 | 0.258 | 0.174 | 26.463 |
| 20240129-0407 | SAAERAVARQRRLFS | STING inactive 6MXE | A1-1000/B1-1000:15-15 | 22.542 | 1.347 | 0.258 | 0.116 | 26.017 |
| 20240127-0639 | MARVSTYETLA | STING inactive 6MXE | A1-1000/B1-1000:11-11 | 40.574 | 1.350 | 0.258 | 0.096 | 28.702 |
| 20240127-0073 | MKTPKILKRLEELRE | STING inactive 6MXE | A1-1000/B1-1000:15-15 | 38.758 | 1.355 | 0.258 | 0.136 | 27.494 |
| 20240129-0592 | GPLRSVAEMLKA | STING inactive 6MXE | A1-1000/B1-1000:12-12 | 37.145 | 1.019 | 0.257 | 0.189 | 26.153 |
| 20240127-0512 | SYLERLSRLARL | STING inactive 6MXE | A1-1000/B1-1000:12-12 | 46.4 | 1.429 | 0.257 | 0.156 | 26.1 |
| 20240127-0095 | SSAERHLEGLRQQSR | STING inactive 6MXE | A1-1000/B1-1000:15-15 | 28.181 | 1.516 | 0.257 | 0.122 | 26.304 |
| 20240127-0094 | SSAERHLEGLRKQSN | STING inactive 6MXE | A1-1000/B1-1000:15-15 | 37.955 | 1.502 | 0.257 | 0.143 | 26.593 |
| 20240129-0679 | MESLLEAARQL | STING inactive 6MXE | A1-1000/B1-1000:11-11 | 36.179 | 1.564 | 0.256 | 0.145 | 26.34 |
| 20240129-0653 | TAWWERLGVKV | STING inactive 6MXE | A1-1000/B1-1000:11-11 | 29.799 | 1.147 | 0.256 | 0.142 | 26.452 |
| 20240129-0445 | SKAFKAAVEINKRTS | STING inactive 6MXE | A1-1000/B1-1000:15-15 | 27.205 | 1.288 | 0.256 | 0.122 | 26.127 |
| 20240127-0050 | SSVILRSARALGIPV | STING inactive 6MXE | A1-1000/B1-1000:15-15 | 17.204 | 1.245 | 0.256 | 0.177 | 25.367 |
| 20240129-0760 | SVWDRHMRQVS | STING inactive 6MXE | A1-1000/B1-1000:11-11 | 35.974 | 1.269 | 0.255 | 0.158 | 26.986 |
| 20240129-0655 | TAWFERLGVRV | STING inactive 6MXE | A1-1000/B1-1000:11-11 | 33.99 | 1.200 | 0.255 | 0.166 | 26.433 |
| 20240129-0688 | LARSVEVLSAL | STING inactive 6MXE | A1-1000/B1-1000:11-11 | 16.811 | 1.315 | 0.254 | 0.245 | 23.051 |
| 20240129-0605 | NVLLRAMEVMFG | STING inactive 6MXE | A1-1000/B1-1000:12-12 | 34.274 | 1.231 | 0.254 | 0.157 | 25.993 |
| 20240129-0590 | SMLRSVAEMLRS | STING inactive 6MXE | A1-1000/B1-1000:12-12 | 34.386 | 1.168 | 0.254 | 0.173 | 26.144 |
| 20240129-0586 | MSKVIEMWKSRL | STING inactive 6MXE | A1-1000/B1-1000:12-12 | 34.548 | 1.299 | 0.254 | 0.142 | 26.527 |
| 20240129-0572 | MKLHELFDEIRE | STING inactive 6MXE | A1-1000/B1-1000:12-12 | 32.861 | 1.476 | 0.254 | 0.139 | 26.409 |
| 20240129-0416 | MSQQSLRMYERIFKS | STING inactive 6MXE | A1-1000/B1-1000:15-15 | 25.411 | 1.336 | 0.254 | 0.136 | 26.257 |
| 20240129-0414 | MSQQSLRMYERIFKS | STING inactive 6MXE | A1-1000/B1-1000:15-15 | 25.411 | 1.339 | 0.254 | 0.136 | 26.257 |
| 20240127-0628 | SARISTYETLA | STING inactive 6MXE | A1-1000/B1-1000:11-11 | 40.697 | 1.290 | 0.254 | 0.101 | 28.606 |
| 20240127-0627 | VAKISTYETLS | STING inactive 6MXE | A1-1000/B1-1000:11-11 | 40.823 | 1.329 | 0.254 | 0.099 | 28.644 |
| 20240127-0098 | KPSLLLQAYDTLTAR | STING inactive 6MXE | A1-1000/B1-1000:15-15 | 27.002 | 1.305 | 0.254 | 0.144 | 26.138 |
| 20240127-0071 | MKTPKILEMLESLRE | STING inactive 6MXE | A1-1000/B1-1000:15-15 | 32.948 | 1.404 | 0.254 | 0.141 | 26.785 |
| 20240127-0062 | SSVVLRGARALGIPL | STING inactive 6MXE | A1-1000/B1-1000:15-15 | 16.477 | 1.207 | 0.254 | 0.196 | 24.578 |
| 20240127-0046 | SAERLRRVENMRNLS | STING inactive 6MXE | A1-1000/B1-1000:15-15 | 23.749 | 1.681 | 0.254 | 0.104 | 27.411 |
| 20240129-0584 | PTKSYLVHRIFR | STING inactive 6MXE | A1-1000/B1-1000:12-12 | 28.757 | 1.571 | 0.253 | 0.355 | 21.371 |
| 20240129-0556 | AVAQDLENIIFK | STING inactive 6MXE | A1-1000/B1-1000:12-12 | 50.058 | 1.503 | 0.252 | 0.144 | 26.689 |
| 20240129-0545 | GPIAGRVSSSLE | STING inactive 6MXE | A1-1000/B1-1000:12-12 | 40.239 | 1.675 | 0.252 | 0.124 | 28.344 |
| 20240129-0474 | SASVSRYAHPGELLP | STING inactive 6MXE | A1-1000/B1-1000:15-15 | 42.087 | 1.191 | 0.252 | 0.147 | 27.43 |
| 20240129-0439 | SRFWELLKKTPSYVA | STING inactive 6MXE | A1-1000/B1-1000:15-15 | 36.635 | 1.544 | 0.252 | 0.144 | 26.331 |
| 20240129-0418 | SYASPLGMVDRIMRG | STING inactive 6MXE | A1-1000/B1-1000:15-15 | 30.001 | 1.202 | 0.252 | 0.196 | 26.162 |
| 20240127-0539 | MVSAQEKLRSIM | STING inactive 6MXE | A1-1000/B1-1000:12-12 | 25.535 | 1.225 | 0.252 | 0.133 | 26.258 |
| 20240127-0077 | MKTPHILKALESLRS | STING inactive 6MXE | A1-1000/B1-1000:15-15 | 31.246 | 1.367 | 0.252 | 0.145 | 26.373 |
| 20240129-0754 | RELLLRSYEVV | STING inactive 6MXE | A1-1000/B1-1000:11-11 | 42.596 | 1.303 | 0.251 | 0.133 | 27.885 |
| 20240129-0447 | SKALRAAIEINKRTS | STING inactive 6MXE | A1-1000/B1-1000:15-15 | 27.489 | 1.307 | 0.251 | 0.132 | 26.016 |
| 20240127-0499 | SYLERLARPVRL | STING inactive 6MXE | A1-1000/B1-1000:12-12 | 46.124 | 1.388 | 0.251 | 0.203 | 25.768 |
| 20240127-0111 | GPSLLLRAYETLTRR | STING inactive 6MXE | A1-1000/B1-1000:15-15 | 30.488 | 1.228 | 0.251 | 0.153 | 26.694 |
| 20240129-0654 | TAWSERLGVRV | STING inactive 6MXE | A1-1000/B1-1000:11-11 | 33.364 | 1.139 | 0.250 | 0.169 | 26.537 |
| 20240129-0589 | GPLRSVAEMLRS | STING inactive 6MXE | A1-1000/B1-1000:12-12 | 37.747 | 1.021 | 0.250 | 0.187 | 26.381 |
| 20240127-0550 | MIAYPEKIANPR | STING inactive 6MXE | A1-1000/B1-1000:12-12 | 43.514 | 1.204 | 0.250 | 0.109 | 28.546 |
| 20240127-0076 | MKTPKILERLEALRE | STING inactive 6MXE | A1-1000/B1-1000:15-15 | 35.397 | 1.329 | 0.250 | 0.127 | 27.419 |
| 20240127-0072 | MKTPKIKERLDSLRS | STING inactive 6MXE | A1-1000/B1-1000:15-15 | 24.635 | 1.430 | 0.250 | 0.153 | 25.198 |
| 20240127-0024 | SEAAAARAARYAKLS | STING inactive 6MXE | A1-1000/B1-1000:15-15 | 24.956 | 1.394 | 0.250 | 0.13 | 25.728 |
| 20240127-0640 | SARLSTYETLA | STING inactive 6MXE | A1-1000/B1-1000:11-11 | 39.55 | 1.297 | 0.249 | 0.099 | 28.69 |
| 20240127-0633 | SARLSTYETLA | STING inactive 6MXE | A1-1000/B1-1000:11-11 | 39.55 | 1.315 | 0.249 | 0.099 | 28.69 |
| 20240127-0632 | SARLSTYETLA | STING inactive 6MXE | A1-1000/B1-1000:11-11 | 39.55 | 1.267 | 0.249 | 0.099 | 28.69 |
| 20240127-0631 | SARLSTYETLA | STING inactive 6MXE | A1-1000/B1-1000:11-11 | 39.55 | 1.315 | 0.249 | 0.099 | 28.69 |
| 20240127-0586 | MDPASLVSLLLK | STING inactive 6MXE | A1-1000/B1-1000:12-12 | 41.719 | 1.191 | 0.249 | 0.176 | 25.831 |
| 20240129-0627 | SNLLKFGEFMLG | STING inactive 6MXE | A1-1000/B1-1000:12-12 | 16.552 | 1.290 | 0.248 | 0.199 | 24.679 |
| 20240129-0554 | SVSQDLENVVFE | STING inactive 6MXE | A1-1000/B1-1000:12-12 | 59.679 | 1.473 | 0.248 | 0.119 | 28.648 |
| 20240129-0463 | MRENLLRSYEKLSSR | STING inactive 6MXE | A1-1000/B1-1000:15-15 | 26.053 | 1.245 | 0.248 | 0.146 | 26.155 |
| 20240127-0589 | MSPASLVSALLK | STING inactive 6MXE | A1-1000/B1-1000:12-12 | 41.21 | 1.214 | 0.248 | 0.172 | 26.032 |
| 20240127-0544 | MTSAQEKLRAIM | STING inactive 6MXE | A1-1000/B1-1000:12-12 | 26.474 | 1.253 | 0.248 | 0.131 | 26.314 |
| 20240127-0535 | MTSAQEKLRAIM | STING inactive 6MXE | A1-1000/B1-1000:12-12 | 26.474 | 1.229 | 0.248 | 0.131 | 26.314 |
| 20240127-0532 | MTSAQEKLRAIM | STING inactive 6MXE | A1-1000/B1-1000:12-12 | 26.474 | 1.227 | 0.248 | 0.131 | 26.314 |
| 20240127-0530 | MTSAQEKLRAIM | STING inactive 6MXE | A1-1000/B1-1000:12-12 | 26.474 | 1.240 | 0.248 | 0.131 | 26.314 |
| 20240127-0482 | MSIVEMNRLLLS | STING inactive 6MXE | A1-1000/B1-1000:12-12 | 20.259 | 1.402 | 0.248 | 0.159 | 25.622 |
| 20240127-0104 | RPSKLLESYETLTAR | STING inactive 6MXE | A1-1000/B1-1000:15-15 | 17.263 | 1.283 | 0.248 | 0.174 | 25.099 |
| 20240129-0644 | MLERLKRIWSG | STING inactive 6MXE | A1-1000/B1-1000:11-11 | 37.229 | 1.280 | 0.247 | 0.195 | 25.959 |
| 20240129-0555 | AVPQTLKNIDFK | STING inactive 6MXE | A1-1000/B1-1000:12-12 | 61.001 | 1.478 | 0.247 | 0.137 | 27.713 |
| 20240129-0406 | SPAERAVARQRRLFS | STING inactive 6MXE | A1-1000/B1-1000:15-15 | 23.55 | 1.368 | 0.247 | 0.107 | 26.511 |
| 20240127-0670 | SEAFRKLFFRP | STING inactive 6MXE | A1-1000/B1-1000:11-11 | 31.465 | 1.432 | 0.247 | 0.137 | 26.625 |
| 20240127-0543 | MISAQEKLRAIM | STING inactive 6MXE | A1-1000/B1-1000:12-12 | 23.418 | 1.192 | 0.247 | 0.123 | 26.26 |
| 20240127-0540 | MTSAQEKLRSIM | STING inactive 6MXE | A1-1000/B1-1000:12-12 | 25.184 | 1.258 | 0.247 | 0.13 | 26.232 |
| 20240127-0536 | MISAQEKLRAIM | STING inactive 6MXE | A1-1000/B1-1000:12-12 | 23.418 | 1.215 | 0.247 | 0.123 | 26.26 |
| 20240127-0534 | MISAQEKLRAIM | STING inactive 6MXE | A1-1000/B1-1000:12-12 | 23.418 | 1.196 | 0.247 | 0.123 | 26.26 |
| 20240127-0533 | MTSAQEKLAAIM | STING inactive 6MXE | A1-1000/B1-1000:12-12 | 18.439 | 1.225 | 0.247 | 0.122 | 26.548 |
| 20240127-0529 | MISAQEKLRAIM | STING inactive 6MXE | A1-1000/B1-1000:12-12 | 23.418 | 1.205 | 0.247 | 0.123 | 26.26 |
| 20240127-0497 | SYLEVLAKPARL | STING inactive 6MXE | A1-1000/B1-1000:12-12 | 46.215 | 1.466 | 0.247 | 0.2 | 25.859 |
| 20240127-0493 | MSVVEMNRFLLR | STING inactive 6MXE | A1-1000/B1-1000:12-12 | 33.935 | 1.504 | 0.247 | 0.139 | 26.251 |
| 20240127-0491 | MSVVEMNRSLLS | STING inactive 6MXE | A1-1000/B1-1000:12-12 | 17.518 | 1.397 | 0.247 | 0.164 | 24.942 |
| 20240127-0490 | MSVVEMNRSLLS | STING inactive 6MXE | A1-1000/B1-1000:12-12 | 17.518 | 1.417 | 0.247 | 0.164 | 24.942 |
| 20240127-0486 | MSVVEMNRSLLS | STING inactive 6MXE | A1-1000/B1-1000:12-12 | 17.518 | 1.397 | 0.247 | 0.164 | 24.942 |
| 20240127-0070 | MKTPKIKAALDALRS | STING inactive 6MXE | A1-1000/B1-1000:15-15 | 31.707 | 1.368 | 0.247 | 0.148 | 26.65 |
| 20240129-0741 | MRLIEMWRSTL | STING inactive 6MXE | A1-1000/B1-1000:11-11 | 32.027 | 1.375 | 0.246 | 0.187 | 26.103 |
| 20240129-0485 | SAAALARTRRLMEPV | STING inactive 6MXE | A1-1000/B1-1000:15-15 | 30.37 | 1.511 | 0.246 | 0.13 | 26.143 |
| 20240127-0119 | DSFRRIALRSMEVFE | STING inactive 6MXE | A1-1000/B1-1000:15-15 | 19.049 | 1.412 | 0.246 | 0.196 | 24.53 |
| 20240127-0087 | SSAERHREGLRKQSR | STING inactive 6MXE | A1-1000/B1-1000:15-15 | 26.356 | 1.511 | 0.246 | 0.128 | 26.009 |
| 20240129-0674 | SDEMLLRTMRE | STING inactive 6MXE | A1-1000/B1-1000:11-11 | 39.171 | 1.698 | 0.245 | 0.162 | 26.304 |
| 20240129-0638 | GAVDMAKFALRS | STING inactive 6MXE | A1-1000/B1-1000:12-12 | 36.682 | 1.208 | 0.245 | 0.171 | 25.917 |
| 20240129-0443 | VDIERLARQLGTAKL | STING inactive 6MXE | A1-1000/B1-1000:15-15 | 19.435 | 1.117 | 0.245 | 0.142 | 25.972 |
| 20240127-0551 | MIAYPERISNVR | STING inactive 6MXE | A1-1000/B1-1000:12-12 | 42.395 | 1.193 | 0.245 | 0.109 | 28.666 |
| 20240129-0680 | MELLLEAARRL | STING inactive 6MXE | A1-1000/B1-1000:11-11 | 30.948 | 1.465 | 0.244 | 0.121 | 26.556 |
| 20240129-0649 | SLFARSYERIH | STING inactive 6MXE | A1-1000/B1-1000:11-11 | 29.877 | 1.398 | 0.244 | 0.172 | 26.901 |
| 20240129-0472 | SERLMGEFMRRAHKG | STING inactive 6MXE | A1-1000/B1-1000:15-15 | 18.226 | 1.277 | 0.244 | 0.17 | 25.448 |
| 20240129-0419 | RYASPLGMVDRIYRG | STING inactive 6MXE | A1-1000/B1-1000:15-15 | 29.692 | 1.276 | 0.244 | 0.184 | 26.295 |
| 20240127-0527 | SLPEIREIFHRE | STING inactive 6MXE | A1-1000/B1-1000:12-12 | 42.114 | 1.284 | 0.244 | 0.142 | 27.287 |
| 20240129-0751 | SAQIIRDRMEA | STING inactive 6MXE | A1-1000/B1-1000:11-11 | 23.56 | 1.481 | 0.243 | 0.165 | 24.576 |
| 20240129-0737 | PTIVERKLLEA | STING inactive 6MXE | A1-1000/B1-1000:11-11 | 32.773 | 1.623 | 0.243 | 0.128 | 27.263 |
| 20240129-0557 | DAAVRARLRALA | STING inactive 6MXE | A1-1000/B1-1000:12-12 | 41.209 | 1.478 | 0.243 | 0.142 | 25.551 |
| 20240127-0638 | VARISTYETLA | STING inactive 6MXE | A1-1000/B1-1000:11-11 | 39.186 | 1.336 | 0.243 | 0.099 | 28.585 |
| 20240127-0542 | MTSAQDKLAKIM | STING inactive 6MXE | A1-1000/B1-1000:12-12 | 24.536 | 1.334 | 0.243 | 0.137 | 26.031 |
| 20240127-0112 | KPSKLLESYDTLVAR | STING inactive 6MXE | A1-1000/B1-1000:15-15 | 16.053 | 1.334 | 0.243 | 0.205 | 24.72 |
| 20240129-0755 | RSLLLRSYETV | STING inactive 6MXE | A1-1000/B1-1000:11-11 | 41.694 | 1.347 | 0.242 | 0.134 | 27.874 |
| 20240129-0600 | SDLMLRSMAVVS | STING inactive 6MXE | A1-1000/B1-1000:12-12 | 15.645 | 1.420 | 0.242 | 0.215 | 23.611 |
| 20240129-0552 | GPVLSIRKIIDK | STING inactive 6MXE | A1-1000/B1-1000:12-12 | 21.772 | 1.237 | 0.242 | 0.228 | 24.218 |
| 20240129-0520 | SRYERARRIATG | STING inactive 6MXE | A1-1000/B1-1000:12-12 | 28.096 | 1.301 | 0.242 | 0.145 | 25.938 |
| 20240129-0519 | SRYERARRIATG | STING inactive 6MXE | A1-1000/B1-1000:12-12 | 28.096 | 1.261 | 0.242 | 0.145 | 25.938 |
| 20240129-0517 | SRYERARRIATG | STING inactive 6MXE | A1-1000/B1-1000:12-12 | 28.096 | 1.220 | 0.242 | 0.145 | 25.938 |
| 20240129-0491 | SMSDILLRSMKVISG | STING inactive 6MXE | A1-1000/B1-1000:15-15 | 35.345 | 1.233 | 0.242 | 0.151 | 26.531 |
| 20240129-0420 | SYASPLGMVDRIMAG | STING inactive 6MXE | A1-1000/B1-1000:15-15 | 29.469 | 1.211 | 0.242 | 0.196 | 26.135 |
| 20240129-0403 | MPASERHRRLIREVW | STING inactive 6MXE | A1-1000/B1-1000:15-15 | 30.674 | 1.339 | 0.242 | 0.138 | 26.655 |
| 20240127-0538 | MISSAEKLRSIL | STING inactive 6MXE | A1-1000/B1-1000:12-12 | 23.273 | 1.330 | 0.242 | 0.142 | 25.669 |
| 20240127-0109 | GPPLLLRAYETLTAR | STING inactive 6MXE | A1-1000/B1-1000:15-15 | 31.315 | 1.278 | 0.242 | 0.142 | 27.091 |
| 20240129-0740 | PTIEERRLLEA | STING inactive 6MXE | A1-1000/B1-1000:11-11 | 17.539 | 1.669 | 0.241 | 0.143 | 26.007 |
| 20240129-0697 | RDLLLDMSRRV | STING inactive 6MXE | A1-1000/B1-1000:11-11 | 21.778 | 1.741 | 0.241 | 0.194 | 25.071 |
| 20240129-0673 | GPSMLLENMSS | STING inactive 6MXE | A1-1000/B1-1000:11-11 | 17.365 | 1.723 | 0.241 | 0.187 | 25.197 |
| 20240129-0587 | MSRVIEMWRSRL | STING inactive 6MXE | A1-1000/B1-1000:12-12 | 34.687 | 1.264 | 0.241 | 0.146 | 26.56 |
| 20240129-0501 | SAPSILDRADLYGSL | STING inactive 6MXE | A1-1000/B1-1000:15-15 | 16.465 | 1.205 | 0.241 | 0.205 | 23.651 |
| 20240127-0714 | GDAQARLQRIF | STING inactive 6MXE | A1-1000/B1-1000:11-11 | 25.321 | 1.774 | 0.241 | 0.142 | 26.367 |
| 20240127-0531 | MVSAQDKLRAIM | STING inactive 6MXE | A1-1000/B1-1000:12-12 | 17.117 | 1.233 | 0.241 | 0.142 | 26.157 |
| 20240127-0524 | ALPEIREIFHRE | STING inactive 6MXE | A1-1000/B1-1000:12-12 | 45.133 | 1.274 | 0.241 | 0.135 | 27.741 |
| 20240127-0522 | ALPEIREIFHRE | STING inactive 6MXE | A1-1000/B1-1000:12-12 | 45.133 | 1.291 | 0.241 | 0.135 | 27.741 |
| 20240127-0484 | MSVVEMNKLLLS | STING inactive 6MXE | A1-1000/B1-1000:12-12 | 17.873 | 1.450 | 0.241 | 0.165 | 25.371 |
| 20240127-0084 | SSAEEHLKGLRKQSA | STING inactive 6MXE | A1-1000/B1-1000:15-15 | 29.947 | 1.534 | 0.241 | 0.111 | 26.33 |
| 20240129-0571 | MLLHKRFEELRE | STING inactive 6MXE | A1-1000/B1-1000:12-12 | 37.925 | 1.438 | 0.240 | 0.134 | 27.494 |
| 20240127-0495 | MSVVEMNKSLLS | STING inactive 6MXE | A1-1000/B1-1000:12-12 | 18.591 | 1.367 | 0.240 | 0.147 | 25.569 |
| 20240127-0492 | MSVVEMNKSLLS | STING inactive 6MXE | A1-1000/B1-1000:12-12 | 18.591 | 1.360 | 0.240 | 0.147 | 25.569 |
| 20240127-0487 | MSVVEMNKSLLS | STING inactive 6MXE | A1-1000/B1-1000:12-12 | 18.591 | 1.362 | 0.240 | 0.147 | 25.569 |
| 20240129-0678 | SSSLLEAARTL | STING inactive 6MXE | A1-1000/B1-1000:11-11 | 36.857 | 1.520 | 0.239 | 0.168 | 26.359 |
| 20240127-0560 | MIAYPEKIANVR | STING inactive 6MXE | A1-1000/B1-1000:12-12 | 43.355 | 1.158 | 0.239 | 0.112 | 28.482 |
| 20240127-0475 | MSNPLMQMFANR | STING inactive 6MXE | A1-1000/B1-1000:12-12 | 18.527 | 1.337 | 0.239 | 0.198 | 24.566 |
| 20240127-0081 | SSAERHLQGLRQQSR | STING inactive 6MXE | A1-1000/B1-1000:15-15 | 32.172 | 1.479 | 0.239 | 0.128 | 25.878 |
| 20240129-0398 | FSAFSAAREAMQEQP | STING inactive 6MXE | A1-1000/B1-1000:15-15 | 15.437 | 1.720 | 0.238 | 0.119 | 26.912 |
| 20240127-0559 | MIAYPERIANPR | STING inactive 6MXE | A1-1000/B1-1000:12-12 | 41.508 | 1.140 | 0.238 | 0.108 | 28.554 |
| 20240127-0556 | MIAYPERIANPR | STING inactive 6MXE | A1-1000/B1-1000:12-12 | 41.508 | 1.184 | 0.238 | 0.108 | 28.554 |
| 20240127-0545 | MIAYPERIANPR | STING inactive 6MXE | A1-1000/B1-1000:12-12 | 41.508 | 1.189 | 0.238 | 0.108 | 28.554 |
| 20240127-0116 | SSFDRIAARSMSVFD | STING inactive 6MXE | A1-1000/B1-1000:15-15 | 14.727 | 1.441 | 0.238 | 0.179 | 24.831 |
| 20240127-0066 | MKTPKILERLNRLRE | STING inactive 6MXE | A1-1000/B1-1000:15-15 | 25.432 | 1.395 | 0.238 | 0.157 | 25.48 |
| 20240129-0691 | SSLLARAVSVF | STING inactive 6MXE | A1-1000/B1-1000:11-11 | 39.907 | 1.427 | 0.237 | 0.189 | 26.52 |
| 20240129-0690 | SSLLARAVSVF | STING inactive 6MXE | A1-1000/B1-1000:11-11 | 39.907 | 1.430 | 0.237 | 0.189 | 26.52 |
| 20240129-0464 | MRANLLRAYEILSSR | STING inactive 6MXE | A1-1000/B1-1000:15-15 | 8.876 | 1.249 | 0.237 | 0.179 | 24.097 |
| 20240129-0409 | SSLLSTLGGRVADIN | STING inactive 6MXE | A1-1000/B1-1000:15-15 | 33.223 | 1.231 | 0.237 | 0.119 | 27.98 |
| 20240127-0477 | SSNPLIQMFERR | STING inactive 6MXE | A1-1000/B1-1000:12-12 | 32.196 | 1.245 | 0.237 | 0.132 | 26.942 |
| 20240127-0476 | SSNPLIQMFERR | STING inactive 6MXE | A1-1000/B1-1000:12-12 | 32.196 | 1.261 | 0.237 | 0.132 | 26.942 |
| 20240127-0468 | SSNPLIQMFERR | STING inactive 6MXE | A1-1000/B1-1000:12-12 | 32.196 | 1.255 | 0.237 | 0.132 | 26.942 |
| 20240127-0067 | MKTPHILSALDSLRE | STING inactive 6MXE | A1-1000/B1-1000:15-15 | 25.961 | 1.496 | 0.237 | 0.162 | 25.825 |
| 20240129-0642 | MLERLRRIWSG | STING inactive 6MXE | A1-1000/B1-1000:11-11 | 35.833 | 1.274 | 0.236 | 0.184 | 26.152 |
| 20240129-0641 | MLERLRRIWSG | STING inactive 6MXE | A1-1000/B1-1000:11-11 | 35.833 | 1.277 | 0.236 | 0.184 | 26.152 |
| 20240129-0625 | SVLRRFGQFLLG | STING inactive 6MXE | A1-1000/B1-1000:12-12 | 36.271 | 1.290 | 0.236 | 0.171 | 26.328 |
| 20240127-0630 | PARLSTYETLS | STING inactive 6MXE | A1-1000/B1-1000:11-11 | 40.3 | 1.277 | 0.236 | 0.102 | 28.594 |
| 20240127-0571 | SLKTSRPKLPWQ | STING inactive 6MXE | A1-1000/B1-1000:12-12 | 34.747 | 1.509 | 0.236 | 0.127 | 27.993 |
| 20240127-0103 | SPSNLLKSYETLTRR | STING inactive 6MXE | A1-1000/B1-1000:15-15 | 22.819 | 1.264 | 0.236 | 0.137 | 25.749 |
| 20240127-0089 | SSAERHLEGMRQTSR | STING inactive 6MXE | A1-1000/B1-1000:15-15 | 33.214 | 1.504 | 0.236 | 0.122 | 26.721 |
| 20240129-0449 | DRRRRLLLRAYETVS | STING inactive 6MXE | A1-1000/B1-1000:15-15 | 38.672 | 1.285 | 0.235 | 0.127 | 27.24 |
| 20240127-0657 | SDAFRKLFFRP | STING inactive 6MXE | A1-1000/B1-1000:11-11 | 30.046 | 1.420 | 0.235 | 0.138 | 26.548 |
| 20240127-0506 | SLLEELAKPVRL | STING inactive 6MXE | A1-1000/B1-1000:12-12 | 45.956 | 1.385 | 0.235 | 0.135 | 26.312 |
| 20240127-0110 | RPSKLLESYNTLTAR | STING inactive 6MXE | A1-1000/B1-1000:15-15 | 8.759 | 1.276 | 0.235 | 0.173 | 24.315 |
| 20240129-0725 | SVIMRVIEMNR | STING inactive 6MXE | A1-1000/B1-1000:11-11 | 21.867 | 1.343 | 0.234 | 0.214 | 24.903 |
| 20240129-0397 | PAALSAARAAMQEKF | STING inactive 6MXE | A1-1000/B1-1000:15-15 | 18.476 | 1.724 | 0.234 | 0.115 | 26.719 |
| 20240127-0547 | MIVYPEKISFVR | STING inactive 6MXE | A1-1000/B1-1000:12-12 | 42.898 | 1.272 | 0.234 | 0.106 | 28.455 |
| 20240127-0514 | ALPEIRRIFHRS | STING inactive 6MXE | A1-1000/B1-1000:12-12 | 43.289 | 1.358 | 0.234 | 0.166 | 26.835 |
| 20240127-0096 | SSAEEHLRGMRKQSR | STING inactive 6MXE | A1-1000/B1-1000:15-15 | 29.481 | 1.582 | 0.234 | 0.115 | 26.305 |
| 20240129-0745 | RVSFDELAWAR | STING inactive 6MXE | A1-1000/B1-1000:11-11 | 37.53 | 1.343 | 0.233 | 0.216 | 26.415 |
| 20240129-0738 | LTIVQRKLLES | STING inactive 6MXE | A1-1000/B1-1000:11-11 | 26.945 | 1.647 | 0.233 | 0.104 | 27.045 |
| 20240129-0568 | SFLLALREASPE | STING inactive 6MXE | A1-1000/B1-1000:12-12 | 43.33 | 1.455 | 0.233 | 0.145 | 27.121 |
| 20240129-0539 | PKAPSVIDVLSL | STING inactive 6MXE | A1-1000/B1-1000:12-12 | 17.728 | 1.198 | 0.233 | 0.184 | 24.613 |
| 20240127-0504 | DLLARLAQRVRL | STING inactive 6MXE | A1-1000/B1-1000:12-12 | 46.656 | 1.476 | 0.233 | 0.152 | 26.406 |
| 20240129-0663 | PAPLIAARLAA | STING inactive 6MXE | A1-1000/B1-1000:11-11 | 14.668 | 1.515 | 0.232 | 0.199 | 24.622 |
| 20240127-0658 | SDAFYSLMFRP | STING inactive 6MXE | A1-1000/B1-1000:11-11 | 14.487 | 1.442 | 0.232 | 0.218 | 23.703 |
| 20240127-0485 | MSVVEMHRSLLS | STING inactive 6MXE | A1-1000/B1-1000:12-12 | 37.758 | 1.478 | 0.232 | 0.126 | 27.157 |
| 20240127-0576 | SLVTLKPKQLWT | STING inactive 6MXE | A1-1000/B1-1000:12-12 | 37.139 | 1.431 | 0.231 | 0.173 | 26.639 |
| 20240127-0573 | SMKTLKPKQLWQ | STING inactive 6MXE | A1-1000/B1-1000:12-12 | 30.588 | 1.448 | 0.231 | 0.161 | 26.188 |
| 20240127-0086 | SSSERHREGLLKQSR | STING inactive 6MXE | A1-1000/B1-1000:15-15 | 23.772 | 1.557 | 0.231 | 0.135 | 25.635 |
| 20240127-0082 | SSAEEHLKGMRKTSA | STING inactive 6MXE | A1-1000/B1-1000:15-15 | 32.394 | 1.562 | 0.231 | 0.12 | 26.443 |
| 20240129-0727 | SVIRRVIEMNR | STING inactive 6MXE | A1-1000/B1-1000:11-11 | 30.062 | 1.382 | 0.230 | 0.143 | 26.42 |
| 20240127-0473 | SSNPLMQMHLNR | STING inactive 6MXE | A1-1000/B1-1000:12-12 | 16.546 | 1.250 | 0.230 | 0.179 | 25.052 |
| 20240127-0471 | SSNPLMQMHLNR | STING inactive 6MXE | A1-1000/B1-1000:12-12 | 16.546 | 1.397 | 0.230 | 0.179 | 25.052 |
| 20240129-0473 | SAPVSRYAHPAELLP | STING inactive 6MXE | A1-1000/B1-1000:15-15 | 20.854 | 1.235 | 0.229 | 0.152 | 25.38 |
| 20240129-0396 | GMVNEEVLLKAARLS | STING inactive 6MXE | A1-1000/B1-1000:15-15 | 38.969 | 1.367 | 0.229 | 0.142 | 26.809 |
| 20240127-0549 | MIVYPEKIANVR | STING inactive 6MXE | A1-1000/B1-1000:12-12 | 43.071 | 1.202 | 0.229 | 0.111 | 28.472 |
| 20240127-0548 | MIVYPEKIANVR | STING inactive 6MXE | A1-1000/B1-1000:12-12 | 43.071 | 1.224 | 0.229 | 0.111 | 28.472 |
| 20240127-0107 | KPSLLLRSYETLTAR | STING inactive 6MXE | A1-1000/B1-1000:15-15 | 26.799 | 1.278 | 0.229 | 0.148 | 26.696 |
| 20240127-0106 | KPSLLLRSYETLTAR | STING inactive 6MXE | A1-1000/B1-1000:15-15 | 26.799 | 1.264 | 0.229 | 0.148 | 26.696 |
| 20240127-0672 | SESFRRLFFRP | STING inactive 6MXE | A1-1000/B1-1000:11-11 | 21.873 | 1.448 | 0.228 | 0.271 | 23.65 |
| 20240127-0105 | SPSLLLKSYETLTSR | STING inactive 6MXE | A1-1000/B1-1000:15-15 | 26.301 | 1.321 | 0.228 | 0.123 | 26.629 |
| 20240129-0573 | SGAWMERLRATK | STING inactive 6MXE | A1-1000/B1-1000:12-12 | 26.604 | 1.295 | 0.227 | 0.147 | 26.24 |
| 20240129-0469 | SEKLMGEFMRRSHRG | STING inactive 6MXE | A1-1000/B1-1000:15-15 | 25.255 | 1.261 | 0.227 | 0.137 | 25.964 |
| 20240129-0460 | SLNEIGGRSIYLILE | STING inactive 6MXE | A1-1000/B1-1000:15-15 | 23.929 | 1.406 | 0.227 | 0.174 | 25.049 |
| 20240127-0469 | SSSPLMRMFERR | STING inactive 6MXE | A1-1000/B1-1000:12-12 | 28.973 | 1.261 | 0.227 | 0.121 | 26.749 |
| 20240127-0123 | SSFDRIALRSMEVFR | STING inactive 6MXE | A1-1000/B1-1000:15-15 | 22.301 | 1.438 | 0.227 | 0.147 | 26.065 |
| 20240129-0598 | SDLMLKSMEVVS | STING inactive 6MXE | A1-1000/B1-1000:12-12 | 20.009 | 1.365 | 0.226 | 0.192 | 24.825 |
| 20240129-0551 | GPVLSVRKIISR | STING inactive 6MXE | A1-1000/B1-1000:12-12 | 20.16 | 1.261 | 0.226 | 0.226 | 24.03 |
| 20240129-0394 | GSINERVLLKAAKLN | STING inactive 6MXE | A1-1000/B1-1000:15-15 | 34.134 | 1.398 | 0.226 | 0.145 | 25.904 |
| 20240127-0660 | SEAFRKLMFRS | STING inactive 6MXE | A1-1000/B1-1000:11-11 | 27.748 | 1.431 | 0.226 | 0.154 | 26.22 |
| 20240127-0472 | SSNPLMQMFLRR | STING inactive 6MXE | A1-1000/B1-1000:12-12 | 32.231 | 1.317 | 0.226 | 0.137 | 26.619 |
| 20240127-0093 | DSSKRHLEGMKKQSA | STING inactive 6MXE | A1-1000/B1-1000:15-15 | 34.257 | 1.662 | 0.226 | 0.115 | 27.103 |
| 20240129-0726 | SNIMKVIEMNR | STING inactive 6MXE | A1-1000/B1-1000:11-11 | 20.878 | 1.291 | 0.225 | 0.14 | 26.25 |
| 20240129-0667 | GLFQEMADERG | STING inactive 6MXE | A1-1000/B1-1000:11-11 | 23.221 | 1.518 | 0.225 | 0.118 | 26.62 |
| 20240129-0457 | MLNEIGGRSIFLILE | STING inactive 6MXE | A1-1000/B1-1000:15-15 | 23.555 | 1.441 | 0.225 | 0.207 | 24.51 |
| 20240129-0452 | SSLRRLLLRSYETVS | STING inactive 6MXE | A1-1000/B1-1000:15-15 | 33.567 | 1.334 | 0.225 | 0.143 | 26.36 |
| 20240129-0451 | SRRRELLLRSYQTVS | STING inactive 6MXE | A1-1000/B1-1000:15-15 | 30.346 | 1.333 | 0.225 | 0.117 | 26.19 |
| 20240127-0719 | SNSLQRFLQIS | STING inactive 6MXE | A1-1000/B1-1000:11-11 | 24.218 | 1.702 | 0.225 | 0.133 | 26.72 |
| 20240127-0505 | SLLEELARPARL | STING inactive 6MXE | A1-1000/B1-1000:12-12 | 28.555 | 1.420 | 0.225 | 0.161 | 25.107 |
| 20240129-0549 | GPVLSVRRIIDR | STING inactive 6MXE | A1-1000/B1-1000:12-12 | 22.352 | 1.109 | 0.224 | 0.201 | 24.804 |
| 20240129-0537 | PKAPNVMDVLSL | STING inactive 6MXE | A1-1000/B1-1000:12-12 | 17.899 | 1.279 | 0.224 | 0.177 | 24.982 |
| 20240127-0625 | SAKLSQYETLA | STING inactive 6MXE | A1-1000/B1-1000:11-11 | 33.966 | 1.298 | 0.224 | 0.148 | 27.494 |
| 20240127-0557 | MIVYPEKIANPR | STING inactive 6MXE | A1-1000/B1-1000:12-12 | 41.205 | 1.206 | 0.224 | 0.109 | 28.226 |
| 20240127-0502 | SLLERLAQPVRL | STING inactive 6MXE | A1-1000/B1-1000:12-12 | 45.314 | 1.399 | 0.224 | 0.138 | 26.478 |
| 20240129-0599 | SDLMLESMRVSS | STING inactive 6MXE | A1-1000/B1-1000:12-12 | 16.41 | 1.450 | 0.223 | 0.184 | 24.38 |
| 20240127-0702 | GNPRLIRRGLL | STING inactive 6MXE | A1-1000/B1-1000:11-11 | 21.864 | 1.362 | 0.223 | 0.203 | 24.498 |
| 20240127-0479 | SSNPLMQMLLRR | STING inactive 6MXE | A1-1000/B1-1000:12-12 | 20.178 | 1.365 | 0.223 | 0.145 | 26.208 |
| 20240127-0478 | SSNPLMQMYERR | STING inactive 6MXE | A1-1000/B1-1000:12-12 | 20.927 | 1.241 | 0.223 | 0.12 | 26.693 |
| 20240127-0090 | SSSERHLEGMRQQSR | STING inactive 6MXE | A1-1000/B1-1000:15-15 | 26.453 | 1.490 | 0.223 | 0.107 | 27.09 |
| 20240129-0768 | LVDASAIARAL | STING inactive 6MXE | A1-1000/B1-1000:11-11 | 17.747 | 1.059 | 0.222 | 0.139 | 25.996 |
| 20240129-0514 | SLLDLSPKNIAV | STING inactive 6MXE | A1-1000/B1-1000:12-12 | 14.336 | 1.414 | 0.222 | 0.171 | 25.081 |
| 20240129-0476 | SASVSRYAHPSELLP | STING inactive 6MXE | A1-1000/B1-1000:15-15 | 34.912 | 1.241 | 0.222 | 0.122 | 26.868 |
| 20240127-0661 | SEAFRRLMFRP | STING inactive 6MXE | A1-1000/B1-1000:11-11 | 28.444 | 1.451 | 0.222 | 0.135 | 26.395 |
| 20240127-0659 | SSDFYSLFFRP | STING inactive 6MXE | A1-1000/B1-1000:11-11 | 7.56 | 1.446 | 0.222 | 0.2 | 23.88 |
| 20240127-0508 | SLLEQLAQPARL | STING inactive 6MXE | A1-1000/B1-1000:12-12 | 27.556 | 1.431 | 0.222 | 0.152 | 25.712 |
| 20240127-0500 | SLLEQLAQPARL | STING inactive 6MXE | A1-1000/B1-1000:12-12 | 27.556 | 1.481 | 0.222 | 0.152 | 25.712 |
| 20240127-0122 | SSFRKIALRSMDVFR | STING inactive 6MXE | A1-1000/B1-1000:15-15 | 22.472 | 1.422 | 0.222 | 0.147 | 26.264 |
| 20240127-0054 | SSVVLRGARALGIPV | STING inactive 6MXE | A1-1000/B1-1000:15-15 | 32.143 | 1.253 | 0.222 | 0.136 | 26.653 |
| 20240129-0395 | SMINEKVLLKAARLN | STING inactive 6MXE | A1-1000/B1-1000:15-15 | 34.373 | 1.324 | 0.221 | 0.143 | 25.974 |
| 20240129-0699 | RELALRAARRL | STING inactive 6MXE | A1-1000/B1-1000:11-11 | 34.072 | 1.746 | 0.220 | 0.137 | 26.55 |
| 20240129-0503 | TAPSIADRQDLYGSL | STING inactive 6MXE | A1-1000/B1-1000:15-15 | 18.848 | 1.274 | 0.220 | 0.142 | 25.818 |
| 20240129-0417 | MYANPLGMVDKIVAG | STING inactive 6MXE | A1-1000/B1-1000:15-15 | 34.68 | 1.270 | 0.220 | 0.146 | 27.175 |
| 20240129-0488 | SAKQLARTRRLMHPV | STING inactive 6MXE | A1-1000/B1-1000:15-15 | 27.465 | 1.544 | 0.219 | 0.126 | 26.119 |
| 20240127-0696 | GDPRLIRQGLL | STING inactive 6MXE | A1-1000/B1-1000:11-11 | 17.141 | 1.239 | 0.219 | 0.152 | 25.52 |
| 20240127-0608 | PRGSVLGLVYP | STING inactive 6MXE | A1-1000/B1-1000:11-11 | 36.906 | 1.250 | 0.219 | 0.181 | 26.639 |
| 20240127-0099 | GPSLLLRSYETLTAR | STING inactive 6MXE | A1-1000/B1-1000:15-15 | 21.953 | 1.238 | 0.219 | 0.121 | 26.503 |
| 20240129-0747 | RVSFDLAAFAR | STING inactive 6MXE | A1-1000/B1-1000:11-11 | 15.913 | 1.307 | 0.218 | 0.179 | 24.554 |
| 20240129-0698 | RDLLLRAAETN | STING inactive 6MXE | A1-1000/B1-1000:11-11 | 16.269 | 1.843 | 0.218 | 0.155 | 25.338 |
| 20240129-0442 | VDLERLARQLGTARL | STING inactive 6MXE | A1-1000/B1-1000:15-15 | 21.58 | 1.176 | 0.218 | 0.125 | 26.361 |
| 20240127-0501 | SLLEELSKPVRL | STING inactive 6MXE | A1-1000/B1-1000:12-12 | 39.932 | 1.427 | 0.218 | 0.123 | 26.259 |
| 20240129-0761 | MARSIEHELRE | STING inactive 6MXE | A1-1000/B1-1000:11-11 | 26.311 | 1.432 | 0.217 | 0.126 | 26.287 |
| 20240129-0575 | SGAFMERLRATK | STING inactive 6MXE | A1-1000/B1-1000:12-12 | 27.811 | 1.303 | 0.217 | 0.136 | 26.296 |
| 20240129-0459 | SLNEIGGRSIFLILE | STING inactive 6MXE | A1-1000/B1-1000:15-15 | 22.373 | 1.422 | 0.217 | 0.223 | 23.962 |
| 20240129-0436 | SMRRAENVRSLFSGI | STING inactive 6MXE | A1-1000/B1-1000:15-15 | 17.112 | 1.515 | 0.217 | 0.182 | 24.975 |
| 20240127-0470 | SSNPLIQMHLRR | STING inactive 6MXE | A1-1000/B1-1000:12-12 | 31.878 | 1.343 | 0.217 | 0.14 | 27.249 |
| 20240127-0127 | SSFARIALRSMDVFR | STING inactive 6MXE | A1-1000/B1-1000:15-15 | 22.774 | 1.475 | 0.217 | 0.146 | 26.308 |
| 20240127-0051 | SSVVLRSARALGIPA | STING inactive 6MXE | A1-1000/B1-1000:15-15 | 16.401 | 1.236 | 0.217 | 0.171 | 25.306 |
| 20240129-0660 | SQMFRLEVRLS | STING inactive 6MXE | A1-1000/B1-1000:11-11 | 54.696 | 1.575 | 0.216 | 0.106 | 28.644 |
| 20240129-0629 | GPARFPAPSVTP | STING inactive 6MXE | A1-1000/B1-1000:12-12 | 32.716 | 1.217 | 0.216 | 0.167 | 26.546 |
| 20240129-0628 | SVLLRFGQFLLG | STING inactive 6MXE | A1-1000/B1-1000:12-12 | 34.214 | 1.338 | 0.216 | 0.139 | 26.872 |
| 20240129-0516 | SLFDLSPKNIAV | STING inactive 6MXE | A1-1000/B1-1000:12-12 | 13.25 | 1.365 | 0.216 | 0.178 | 24.815 |
| 20240129-0446 | SLAFKAAVDINKLVS | STING inactive 6MXE | A1-1000/B1-1000:15-15 | 21.74 | 1.373 | 0.216 | 0.144 | 26.147 |
| 20240129-0410 | SSLLSVLGGRVASIN | STING inactive 6MXE | A1-1000/B1-1000:15-15 | 28.094 | 1.257 | 0.216 | 0.12 | 27.218 |
| 20240127-0637 | SAKVSQYETLS | STING inactive 6MXE | A1-1000/B1-1000:11-11 | 37.407 | 1.382 | 0.216 | 0.095 | 28.633 |
| 20240127-0552 | MIVYPERIANPR | STING inactive 6MXE | A1-1000/B1-1000:12-12 | 39.509 | 1.193 | 0.216 | 0.107 | 28.125 |
| 20240127-0525 | SLPEIRSIFHRE | STING inactive 6MXE | A1-1000/B1-1000:12-12 | 36.8 | 1.283 | 0.216 | 0.14 | 27.27 |
| 20240127-0100 | SPSLLLKSYETLTAR | STING inactive 6MXE | A1-1000/B1-1000:15-15 | 17.737 | 1.274 | 0.216 | 0.142 | 26.114 |
| 20240127-0097 | SPSLLLKSYETLTAR | STING inactive 6MXE | A1-1000/B1-1000:15-15 | 17.737 | 1.292 | 0.216 | 0.142 | 26.114 |
| 20240127-0664 | SDSFKRLMFRS | STING inactive 6MXE | A1-1000/B1-1000:11-11 | 27.206 | 1.478 | 0.215 | 0.154 | 26.117 |
| 20240129-0515 | SSVDLAPKNIAV | STING inactive 6MXE | A1-1000/B1-1000:12-12 | 40.231 | 1.360 | 0.214 | 0.108 | 28.257 |
| 20240129-0462 | MRENLLKSYEIISSR | STING inactive 6MXE | A1-1000/B1-1000:15-15 | 16.841 | 1.176 | 0.214 | 0.166 | 24.742 |
| 20240127-0716 | ASARERLLKVN | STING inactive 6MXE | A1-1000/B1-1000:11-11 | 25.973 | 1.777 | 0.214 | 0.135 | 26.217 |
| 20240127-0618 | FVNLANLTIPR | STING inactive 6MXE | A1-1000/B1-1000:11-11 | 17.272 | 1.383 | 0.214 | 0.178 | 24.219 |
| 20240127-0570 | SMKTLKPKQLWR | STING inactive 6MXE | A1-1000/B1-1000:12-12 | 30.978 | 1.401 | 0.214 | 0.158 | 26.175 |
| 20240127-0108 | KPSLLLRSYETLTSR | STING inactive 6MXE | A1-1000/B1-1000:15-15 | 19.93 | 1.312 | 0.214 | 0.146 | 25.522 |
| 20240129-0611 | SSIWSSVAGLRA | STING inactive 6MXE | A1-1000/B1-1000:12-12 | 41.199 | 1.733 | 0.213 | 0.189 | 26.795 |
| 20240129-0412 | SSLLHTLGGNVAKLN | STING inactive 6MXE | A1-1000/B1-1000:15-15 | 24.825 | 1.275 | 0.213 | 0.148 | 26.993 |
| 20240127-0701 | GNPALVKEGLL | STING inactive 6MXE | A1-1000/B1-1000:11-11 | 16.091 | 1.368 | 0.213 | 0.165 | 24.881 |
| 20240127-0526 | ALPEIREIFHRR | STING inactive 6MXE | A1-1000/B1-1000:12-12 | 39.866 | 1.242 | 0.213 | 0.126 | 27.566 |
| 20240129-0576 | SGAFMERLRATR | STING inactive 6MXE | A1-1000/B1-1000:12-12 | 24.802 | 1.225 | 0.212 | 0.136 | 25.997 |
| 20240127-0663 | SEAFRKLFFRS | STING inactive 6MXE | A1-1000/B1-1000:11-11 | 30.776 | 1.380 | 0.212 | 0.143 | 26.545 |
| 20240129-0524 | RREQMLRTARIV | STING inactive 6MXE | A1-1000/B1-1000:12-12 | 29.386 | 1.460 | 0.211 | 0.132 | 26.716 |
| 20240129-0518 | SRYERARRITTG | STING inactive 6MXE | A1-1000/B1-1000:12-12 | 30.192 | 1.277 | 0.211 | 0.126 | 26.344 |
| 20240129-0495 | MSSRLPFERGREMLA | STING inactive 6MXE | A1-1000/B1-1000:15-15 | 17.068 | 1.508 | 0.211 | 0.147 | 26.109 |
| 20240129-0458 | PLNAVGGNSINLILR | STING inactive 6MXE | A1-1000/B1-1000:15-15 | 17.619 | 1.465 | 0.211 | 0.172 | 25.12 |
| 20240127-0600 | PKPAILGIFYP | STING inactive 6MXE | A1-1000/B1-1000:11-11 | 26.477 | 1.153 | 0.211 | 0.281 | 22.556 |
| 20240127-0564 | ELKTLKPKQLWR | STING inactive 6MXE | A1-1000/B1-1000:12-12 | 31.375 | 1.447 | 0.211 | 0.142 | 26.271 |
| 20240127-0120 | SSFDRISLRSMEVFR | STING inactive 6MXE | A1-1000/B1-1000:15-15 | 25.517 | 1.388 | 0.211 | 0.135 | 27.156 |
| 20240127-0114 | SSFDRISLRSMEVFR | STING inactive 6MXE | A1-1000/B1-1000:15-15 | 25.517 | 1.422 | 0.211 | 0.135 | 27.156 |
| 20240129-0597 | SDLMLRSYEVVS | STING inactive 6MXE | A1-1000/B1-1000:12-12 | 19.234 | 1.402 | 0.210 | 0.16 | 24.995 |
| 20240127-0519 | ALPEIRSIFHVE | STING inactive 6MXE | A1-1000/B1-1000:12-12 | 30.322 | 1.339 | 0.210 | 0.142 | 26.74 |
| 20240127-0510 | SYLELLSRPVRL | STING inactive 6MXE | A1-1000/B1-1000:12-12 | 43.164 | 1.362 | 0.210 | 0.126 | 26.19 |
| 20240129-0724 | MQVAPIRTALP | STING inactive 6MXE | A1-1000/B1-1000:11-11 | 19.048 | 1.356 | 0.209 | 0.169 | 27.005 |
| 20240127-0671 | SSSFRKLFFRS | STING inactive 6MXE | A1-1000/B1-1000:11-11 | 22.153 | 1.433 | 0.209 | 0.239 | 23.966 |
| 20240127-0667 | SSSFRKLFFRS | STING inactive 6MXE | A1-1000/B1-1000:11-11 | 22.153 | 1.422 | 0.209 | 0.239 | 23.966 |
| 20240127-0602 | PKGSILGIYYP | STING inactive 6MXE | A1-1000/B1-1000:11-11 | 21.375 | 1.154 | 0.209 | 0.18 | 24.774 |
| 20240127-0601 | PRGSVLGIVYP | STING inactive 6MXE | A1-1000/B1-1000:11-11 | 40.156 | 1.243 | 0.209 | 0.13 | 28.088 |
| 20240127-0509 | SLLEKLSQPVRL | STING inactive 6MXE | A1-1000/B1-1000:12-12 | 44.476 | 1.453 | 0.209 | 0.125 | 26.525 |
| 20240127-0128 | DSFRRIALRSMSVFS | STING inactive 6MXE | A1-1000/B1-1000:15-15 | 22 | 1.436 | 0.209 | 0.151 | 26.154 |
| 20240129-0643 | MLSKLRRIWSG | STING inactive 6MXE | A1-1000/B1-1000:11-11 | 35.259 | 1.363 | 0.207 | 0.187 | 26.161 |
| 20240127-0709 | SNARERLLRVF | STING inactive 6MXE | A1-1000/B1-1000:11-11 | 26.845 | 1.716 | 0.207 | 0.149 | 26.189 |
| 20240127-0700 | GDPRLVRRGLL | STING inactive 6MXE | A1-1000/B1-1000:11-11 | 32.072 | 1.261 | 0.207 | 0.125 | 26.867 |
| 20240129-0661 | PASRIAARLAA | STING inactive 6MXE | A1-1000/B1-1000:11-11 | 33.611 | 1.517 | 0.206 | 0.141 | 26.285 |
| 20240129-0652 | SLFSRSYTITH | STING inactive 6MXE | A1-1000/B1-1000:11-11 | 32.847 | 1.366 | 0.206 | 0.12 | 28.15 |
| 20240129-0570 | MLLHRRFDELRE | STING inactive 6MXE | A1-1000/B1-1000:12-12 | 25.897 | 1.466 | 0.206 | 0.179 | 25.08 |
| 20240127-0669 | SDSFRKLFFRP | STING inactive 6MXE | A1-1000/B1-1000:11-11 | 9.339 | 1.442 | 0.206 | 0.167 | 24.667 |
| 20240127-0619 | FINLSNLTIPR | STING inactive 6MXE | A1-1000/B1-1000:11-11 | 17.943 | 1.444 | 0.206 | 0.17 | 24.675 |
| 20240127-0118 | SSFDRIALRSMSVFE | STING inactive 6MXE | A1-1000/B1-1000:15-15 | 18.972 | 1.412 | 0.206 | 0.133 | 26.224 |
| 20240127-0088 | SSSEEHLRGLRLQSR | STING inactive 6MXE | A1-1000/B1-1000:15-15 | 23.634 | 1.549 | 0.206 | 0.17 | 25.035 |
| 20240129-0704 | SAPGLLPPLLP | STING inactive 6MXE | A1-1000/B1-1000:11-11 | 41.892 | 1.110 | 0.205 | 0.209 | 23.947 |
| 20240129-0692 | SSLLARAVSVN | STING inactive 6MXE | A1-1000/B1-1000:11-11 | 36.33 | 1.423 | 0.205 | 0.177 | 26.461 |
| 20240129-0689 | SSLLARAVSVN | STING inactive 6MXE | A1-1000/B1-1000:11-11 | 36.33 | 1.447 | 0.205 | 0.177 | 26.461 |
| 20240129-0685 | LRRSVEVLSAL | STING inactive 6MXE | A1-1000/B1-1000:11-11 | 22.761 | 1.268 | 0.205 | 0.124 | 26.432 |
| 20240129-0479 | SNKTLPVTARGRLLR | STING inactive 6MXE | A1-1000/B1-1000:15-15 | 34.1 | 1.303 | 0.205 | 0.135 | 26.876 |
| 20240127-0705 | SDAAARFARVS | STING inactive 6MXE | A1-1000/B1-1000:11-11 | 25.485 | 1.705 | 0.205 | 0.145 | 26.317 |
| 20240127-0662 | SDSFYSLFFRS | STING inactive 6MXE | A1-1000/B1-1000:11-11 | 30.039 | 1.429 | 0.205 | 0.129 | 26.838 |
| 20240127-0624 | FKHLASLVIPV | STING inactive 6MXE | A1-1000/B1-1000:11-11 | 18.595 | 1.426 | 0.205 | 0.171 | 25.129 |
| 20240127-0520 | ALREIRRIFHVE | STING inactive 6MXE | A1-1000/B1-1000:12-12 | 26.991 | 1.344 | 0.205 | 0.173 | 25.773 |
| 20240127-0494 | MAVVDMNRSLLS | STING inactive 6MXE | A1-1000/B1-1000:12-12 | 17.895 | 1.409 | 0.205 | 0.157 | 25.025 |
| 20240127-0474 | SSNPLMQMHLRR | STING inactive 6MXE | A1-1000/B1-1000:12-12 | 26.035 | 1.305 | 0.205 | 0.145 | 26.377 |
| 20240129-0763 | SASSIEHELRS | STING inactive 6MXE | A1-1000/B1-1000:11-11 | 29.254 | 1.555 | 0.204 | 0.142 | 26.147 |
| 20240129-0578 | MRLYLSTGPILP | STING inactive 6MXE | A1-1000/B1-1000:12-12 | 39.826 | 1.328 | 0.204 | 0.118 | 28.469 |
| 20240129-0498 | MVRSTAEMSPKVKAP | STING inactive 6MXE | A1-1000/B1-1000:15-15 | 32.256 | 1.124 | 0.204 | 0.118 | 27.408 |
| 20240129-0475 | SASVPRFAHPGELLP | STING inactive 6MXE | A1-1000/B1-1000:15-15 | 28.971 | 1.195 | 0.204 | 0.112 | 26.917 |
| 20240129-0411 | SSLLKNLGGKVASLS | STING inactive 6MXE | A1-1000/B1-1000:15-15 | 24.562 | 1.295 | 0.204 | 0.146 | 26.721 |
| 20240127-0615 | FFVMPTMIFPR | STING inactive 6MXE | A1-1000/B1-1000:11-11 | 19.286 | 1.650 | 0.204 | 0.15 | 25.453 |
| 20240127-0611 | WKELANLRIPV | STING inactive 6MXE | A1-1000/B1-1000:11-11 | 31.08 | 1.398 | 0.204 | 0.153 | 26.444 |
| 20240127-0553 | MIVYPERISNVR | STING inactive 6MXE | A1-1000/B1-1000:12-12 | 36.974 | 1.194 | 0.204 | 0.108 | 28.104 |
| 20240127-0546 | MIVYPERIGNVR | STING inactive 6MXE | A1-1000/B1-1000:12-12 | 41.54 | 1.198 | 0.204 | 0.107 | 28.332 |
| 20240127-0124 | SSFDRIALRSMSVFD | STING inactive 6MXE | A1-1000/B1-1000:15-15 | 18.093 | 1.444 | 0.204 | 0.136 | 26.135 |
| 20240129-0756 | RRLLLRAYEVT | STING inactive 6MXE | A1-1000/B1-1000:11-11 | 39.176 | 1.297 | 0.203 | 0.14 | 27.798 |
| 20240129-0461 | MRKNLLKAYEIISSK | STING inactive 6MXE | A1-1000/B1-1000:15-15 | 29.627 | 1.208 | 0.203 | 0.186 | 24.172 |
| 20240127-0690 | GNPNLIRLGRL | STING inactive 6MXE | A1-1000/B1-1000:11-11 | 36.979 | 1.381 | 0.203 | 0.12 | 27.607 |
| 20240127-0665 | SSSFRRLFFRS | STING inactive 6MXE | A1-1000/B1-1000:11-11 | 22.439 | 1.457 | 0.203 | 0.22 | 24.335 |
| 20240129-0712 | SLPKVFKDIRL | STING inactive 6MXE | A1-1000/B1-1000:11-11 | 23.659 | 1.311 | 0.202 | 0.13 | 26.814 |
| 20240129-0493 | SSSRLPFERGRRDLA | STING inactive 6MXE | A1-1000/B1-1000:15-15 | 24.148 | 1.532 | 0.202 | 0.19 | 24.941 |
| 20240127-0605 | PSGSILGIFKP | STING inactive 6MXE | A1-1000/B1-1000:11-11 | 14.71 | 1.194 | 0.202 | 0.175 | 24.678 |
| 20240127-0563 | SMKTSRPKLLEV | STING inactive 6MXE | A1-1000/B1-1000:12-12 | 35.59 | 1.502 | 0.202 | 0.132 | 27.126 |
| 20240127-0115 | SSLDKIRLKSMSVFD | STING inactive 6MXE | A1-1000/B1-1000:15-15 | 18.912 | 1.443 | 0.202 | 0.14 | 26.348 |
| 20240129-0664 | ASPLIARRLSS | STING inactive 6MXE | A1-1000/B1-1000:11-11 | 20.326 | 1.566 | 0.201 | 0.121 | 26.405 |
| 20240129-0499 | MVRSVAELSPPVTAP | STING inactive 6MXE | A1-1000/B1-1000:15-15 | 24.348 | 1.218 | 0.201 | 0.144 | 25.638 |
| 20240127-0636 | VATLETYYTLS | STING inactive 6MXE | A1-1000/B1-1000:11-11 | 33.595 | 1.406 | 0.201 | 0.119 | 27.686 |
| 20240127-0626 | SARVSQYETLA | STING inactive 6MXE | A1-1000/B1-1000:11-11 | 32.913 | 1.274 | 0.201 | 0.151 | 27.174 |
| 20240127-0513 | TLPEIREIFHVE | STING inactive 6MXE | A1-1000/B1-1000:12-12 | 34.41 | 1.335 | 0.201 | 0.129 | 27.354 |
| 20240127-0489 | SAVVAMNRSLLS | STING inactive 6MXE | A1-1000/B1-1000:12-12 | 18.029 | 1.478 | 0.201 | 0.153 | 25.49 |
| 20240129-0717 | GPLLLRPIQRW | STING inactive 6MXE | A1-1000/B1-1000:11-11 | 34.731 | 1.302 | 0.200 | 0.175 | 26.78 |
| 20240129-0580 | RRLYLSTGPVLP | STING inactive 6MXE | A1-1000/B1-1000:12-12 | 37.609 | 1.348 | 0.200 | 0.122 | 28.152 |
| 20240129-0497 | MVKSVAEMSPKVRAP | STING inactive 6MXE | A1-1000/B1-1000:15-15 | 25.53 | 1.141 | 0.200 | 0.12 | 26.627 |
| 20240127-0689 | GDPRLIRLGRL | STING inactive 6MXE | A1-1000/B1-1000:11-11 | 21.142 | 1.252 | 0.200 | 0.177 | 24.704 |
| 20240127-0606 | PPGSILGIFYP | STING inactive 6MXE | A1-1000/B1-1000:11-11 | 33.259 | 1.192 | 0.200 | 0.128 | 27.357 |
| 20240127-0596 | PPPSILGIFKP | STING inactive 6MXE | A1-1000/B1-1000:11-11 | 15.592 | 1.227 | 0.200 | 0.167 | 24.821 |
| 20240127-0121 | SSMDRIRLRSMSVFR | STING inactive 6MXE | A1-1000/B1-1000:15-15 | 19.515 | 1.423 | 0.200 | 0.228 | 24.267 |
| 20240129-0746 | VVSFDQRAFAR | STING inactive 6MXE | A1-1000/B1-1000:11-11 | 21.154 | 1.366 | 0.199 | 0.128 | 26.302 |
| 20240129-0666 | GLISDAADAYG | STING inactive 6MXE | A1-1000/B1-1000:11-11 | 13.397 | 1.429 | 0.199 | 0.158 | 25.067 |
| 20240129-0748 | VVSFDERAWAR | STING inactive 6MXE | A1-1000/B1-1000:11-11 | 21.163 | 1.409 | 0.198 | 0.147 | 26.011 |
| 20240129-0713 | PNPLPEKIVFP | STING inactive 6MXE | A1-1000/B1-1000:11-11 | 17.272 | 1.401 | 0.198 | 0.131 | 25.859 |
| 20240129-0703 | EAPQLLPPLLP | STING inactive 6MXE | A1-1000/B1-1000:11-11 | 44.039 | 1.048 | 0.198 | 0.171 | 24.943 |
| 20240129-0478 | SVKTLPVEARGRLLK | STING inactive 6MXE | A1-1000/B1-1000:15-15 | 25.298 | 1.221 | 0.198 | 0.147 | 25.33 |
| 20240127-0686 | SAPLKALRPLG | STING inactive 6MXE | A1-1000/B1-1000:11-11 | 34.208 | 1.234 | 0.198 | 0.137 | 27.095 |
| 20240127-0683 | SAPLKALRPLG | STING inactive 6MXE | A1-1000/B1-1000:11-11 | 34.208 | 1.215 | 0.198 | 0.137 | 27.095 |
| 20240127-0595 | PPGAVLGIFRP | STING inactive 6MXE | A1-1000/B1-1000:11-11 | 32.082 | 1.229 | 0.198 | 0.137 | 27.376 |
| 20240129-0682 | SSRLSRLTSGS | STING inactive 6MXE | A1-1000/B1-1000:11-11 | 24.037 | 1.821 | 0.197 | 0.206 | 24.901 |
| 20240127-0607 | PKPSILGIFYP | STING inactive 6MXE | A1-1000/B1-1000:11-11 | 33.61 | 1.153 | 0.197 | 0.129 | 27.34 |
| 20240127-0516 | ALPEIRRGFHVS | STING inactive 6MXE | A1-1000/B1-1000:12-12 | 46.657 | 1.355 | 0.197 | 0.169 | 26.865 |
| 20240127-0704 | GNPRLVREGLL | STING inactive 6MXE | A1-1000/B1-1000:11-11 | 17.51 | 1.372 | 0.196 | 0.148 | 25.587 |
| 20240127-0680 | SVPLKALRPLG | STING inactive 6MXE | A1-1000/B1-1000:11-11 | 34.135 | 1.254 | 0.196 | 0.129 | 27.301 |
| 20240127-0554 | MIAYPLRIAFPR | STING inactive 6MXE | A1-1000/B1-1000:12-12 | 34.994 | 1.246 | 0.196 | 0.14 | 26.518 |
| 20240127-0521 | SLPEIRRHFHVR | STING inactive 6MXE | A1-1000/B1-1000:12-12 | 24.511 | 1.346 | 0.196 | 0.157 | 26.093 |
| 20240127-0496 | MAVVEMNRSLLS | STING inactive 6MXE | A1-1000/B1-1000:12-12 | 18.354 | 1.370 | 0.196 | 0.156 | 25.29 |
| 20240127-0481 | MAVVEMNRSLLS | STING inactive 6MXE | A1-1000/B1-1000:12-12 | 18.354 | 1.377 | 0.196 | 0.156 | 25.29 |
| 20240129-0764 | MAYSIEHLVRE | STING inactive 6MXE | A1-1000/B1-1000:11-11 | 24.184 | 1.485 | 0.195 | 0.159 | 25.668 |
| 20240129-0662 | PAPLIHERLAS | STING inactive 6MXE | A1-1000/B1-1000:11-11 | 20.939 | 1.473 | 0.195 | 0.152 | 25.21 |
| 20240129-0640 | GAVDMSRFALAS | STING inactive 6MXE | A1-1000/B1-1000:12-12 | 24.283 | 1.269 | 0.195 | 0.134 | 26.516 |
| 20240129-0639 | GAVDYSRFYLKS | STING inactive 6MXE | A1-1000/B1-1000:12-12 | 25.815 | 1.249 | 0.195 | 0.145 | 25.507 |
| 20240129-0480 | SNKTLPVERRGRALR | STING inactive 6MXE | A1-1000/B1-1000:15-15 | 29.908 | 1.353 | 0.195 | 0.123 | 26.914 |
| 20240127-0610 | FVELANITIPR | STING inactive 6MXE | A1-1000/B1-1000:11-11 | 16.544 | 1.484 | 0.195 | 0.17 | 24.409 |
| 20240127-0604 | PRGSILGIFYP | STING inactive 6MXE | A1-1000/B1-1000:11-11 | 32.413 | 1.186 | 0.195 | 0.133 | 26.854 |
| 20240127-0523 | SLPEIREIFHVE | STING inactive 6MXE | A1-1000/B1-1000:12-12 | 32.194 | 1.278 | 0.195 | 0.14 | 27.12 |
| 20240127-0518 | ALPEIREIFHVE | STING inactive 6MXE | A1-1000/B1-1000:12-12 | 35.515 | 1.306 | 0.195 | 0.133 | 27.106 |
| 20240127-0515 | SLPEIREIFHVE | STING inactive 6MXE | A1-1000/B1-1000:12-12 | 32.194 | 1.291 | 0.195 | 0.14 | 27.12 |
| 20240127-0126 | DVFDRISLRSMSVFR | STING inactive 6MXE | A1-1000/B1-1000:15-15 | 17.922 | 1.542 | 0.195 | 0.13 | 26.074 |
| 20240129-0579 | MKLYLSTGPVLP | STING inactive 6MXE | A1-1000/B1-1000:12-12 | 36.802 | 1.305 | 0.194 | 0.127 | 27.938 |
| 20240127-0703 | GDPRLIKLGRL | STING inactive 6MXE | A1-1000/B1-1000:11-11 | 25.804 | 1.271 | 0.194 | 0.139 | 26.093 |
| 20240127-0648 | SSSAAPLPLLP | STING inactive 6MXE | A1-1000/B1-1000:11-11 | 18.844 | 1.394 | 0.194 | 0.178 | 24.552 |
| 20240127-0642 | RSSAAPLPLLP | STING inactive 6MXE | A1-1000/B1-1000:11-11 | 17.46 | 1.455 | 0.194 | 0.18 | 24.256 |
| 20240127-0641 | SSSAAKLPRIP | STING inactive 6MXE | A1-1000/B1-1000:11-11 | 32.941 | 1.497 | 0.194 | 0.119 | 27.451 |
| 20240127-0622 | FINLSNITIPR | STING inactive 6MXE | A1-1000/B1-1000:11-11 | 16.893 | 1.454 | 0.194 | 0.16 | 24.777 |
| 20240127-0613 | FIHLANMVIPR | STING inactive 6MXE | A1-1000/B1-1000:11-11 | 16.716 | 1.457 | 0.194 | 0.165 | 24.848 |
| 20240127-0555 | MIVYPDKIANVR | STING inactive 6MXE | A1-1000/B1-1000:12-12 | 20.552 | 1.181 | 0.194 | 0.164 | 24.86 |
| 20240129-0683 | PSVLSKKFSKE | STING inactive 6MXE | A1-1000/B1-1000:11-11 | 31.981 | 1.823 | 0.193 | 0.136 | 27.13 |
| 20240129-0636 | MSTSSPLPPTVP | STING inactive 6MXE | A1-1000/B1-1000:12-12 | 17.271 | 1.233 | 0.193 | 0.13 | 26.367 |
| 20240129-0635 | MSHDGPLPPTPP | STING inactive 6MXE | A1-1000/B1-1000:12-12 | 19.954 | 1.261 | 0.193 | 0.137 | 26.144 |
| 20240129-0581 | ASASYETHRIFR | STING inactive 6MXE | A1-1000/B1-1000:12-12 | 24.598 | 1.609 | 0.193 | 0.16 | 25.784 |
| 20240129-0567 | SFLLELRESSPE | STING inactive 6MXE | A1-1000/B1-1000:12-12 | 40.613 | 1.434 | 0.193 | 0.127 | 27.588 |
| 20240129-0546 | GPLAGRIQSSLA | STING inactive 6MXE | A1-1000/B1-1000:12-12 | 15.828 | 1.630 | 0.193 | 0.16 | 24.875 |
| 20240129-0494 | MSSRLPSERGRADLA | STING inactive 6MXE | A1-1000/B1-1000:15-15 | 16.226 | 1.586 | 0.193 | 0.13 | 26.36 |
| 20240127-0656 | SSSAMPLPLLP | STING inactive 6MXE | A1-1000/B1-1000:11-11 | 19.222 | 1.379 | 0.193 | 0.174 | 24.673 |
| 20240127-0647 | ASSAAPLPLIP | STING inactive 6MXE | A1-1000/B1-1000:11-11 | 19.074 | 1.444 | 0.193 | 0.176 | 24.548 |
| 20240127-0597 | PPGSVLGIFKP | STING inactive 6MXE | A1-1000/B1-1000:11-11 | 15.717 | 1.178 | 0.193 | 0.159 | 25.362 |
| 20240127-0561 | SMKTSKPKMLWR | STING inactive 6MXE | A1-1000/B1-1000:12-12 | 27.494 | 1.436 | 0.193 | 0.171 | 25.118 |
| 20240129-0733 | DRKSAISGIVR | STING inactive 6MXE | A1-1000/B1-1000:11-11 | 22.039 | 1.244 | 0.192 | 0.133 | 26.087 |
| 20240129-0702 | SAPQLLPPLLP | STING inactive 6MXE | A1-1000/B1-1000:11-11 | 44.501 | 1.109 | 0.192 | 0.156 | 25.165 |
| 20240129-0700 | RSSLLNMYRTV | STING inactive 6MXE | A1-1000/B1-1000:11-11 | 33.701 | 1.836 | 0.192 | 0.146 | 26.708 |
| 20240129-0630 | GPAKFPAPSVRR | STING inactive 6MXE | A1-1000/B1-1000:12-12 | 29.315 | 1.244 | 0.192 | 0.122 | 26.513 |
| 20240127-0698 | GDPRLIRVGRL | STING inactive 6MXE | A1-1000/B1-1000:11-11 | 20.619 | 1.269 | 0.192 | 0.176 | 24.727 |
| 20240129-0765 | LVDASAIARAR | STING inactive 6MXE | A1-1000/B1-1000:11-11 | 18.356 | 1.012 | 0.191 | 0.148 | 25.57 |
| 20240129-0714 | RDPLPRKIVWP | STING inactive 6MXE | A1-1000/B1-1000:11-11 | 29.597 | 1.378 | 0.191 | 0.116 | 26.61 |
| 20240129-0693 | PELVLSRSFMQ | STING inactive 6MXE | A1-1000/B1-1000:11-11 | 27.26 | 1.679 | 0.191 | 0.145 | 26.974 |
| 20240129-0500 | MVRSVAELSPPVKAP | STING inactive 6MXE | A1-1000/B1-1000:15-15 | 24.35 | 1.125 | 0.191 | 0.121 | 26.699 |
| 20240129-0477 | SVKTLPEVVRGRRLR | STING inactive 6MXE | A1-1000/B1-1000:15-15 | 26.337 | 1.228 | 0.191 | 0.132 | 26.123 |
| 20240127-0697 | GDPRLIRRGLL | STING inactive 6MXE | A1-1000/B1-1000:11-11 | 26.927 | 1.229 | 0.191 | 0.127 | 26.422 |
| 20240127-0692 | GDPRLIRRGLL | STING inactive 6MXE | A1-1000/B1-1000:11-11 | 26.927 | 1.214 | 0.191 | 0.127 | 26.422 |
| 20240127-0668 | SSAFRKLFFRP | STING inactive 6MXE | A1-1000/B1-1000:11-11 | 29.943 | 1.404 | 0.191 | 0.132 | 26.598 |
| 20240127-0614 | FIHLPNIVIPR | STING inactive 6MXE | A1-1000/B1-1000:11-11 | 27.765 | 1.399 | 0.191 | 0.26 | 23.517 |
| 20240127-0609 | FIHLPNIVIPR | STING inactive 6MXE | A1-1000/B1-1000:11-11 | 27.765 | 1.440 | 0.191 | 0.26 | 23.517 |
| 20240127-0517 | ALPEIREIFHME | STING inactive 6MXE | A1-1000/B1-1000:12-12 | 19.898 | 1.333 | 0.191 | 0.153 | 25.787 |
| 20240129-0631 | SYARKPAPAVVP | STING inactive 6MXE | A1-1000/B1-1000:12-12 | 28.905 | 1.302 | 0.190 | 0.119 | 26.691 |
| 20240129-0612 | MSIWSNVSGLRS | STING inactive 6MXE | A1-1000/B1-1000:12-12 | 36.261 | 1.729 | 0.190 | 0.129 | 26.922 |
| 20240129-0582 | SMSAYFTHRIFR | STING inactive 6MXE | A1-1000/B1-1000:12-12 | 29.752 | 1.571 | 0.190 | 0.254 | 23.051 |
| 20240129-0435 | SMRRARAVRSMFRGM | STING inactive 6MXE | A1-1000/B1-1000:15-15 | 26.069 | 1.606 | 0.190 | 0.135 | 25.896 |
| 20240127-0688 | SAPLKSIRPLG | STING inactive 6MXE | A1-1000/B1-1000:11-11 | 33.366 | 1.300 | 0.190 | 0.143 | 26.722 |
| 20240127-0617 | FKKLSNMTIPV | STING inactive 6MXE | A1-1000/B1-1000:11-11 | 27.414 | 1.445 | 0.190 | 0.117 | 26.871 |
| 20240127-0565 | SMRTSRPKELWR | STING inactive 6MXE | A1-1000/B1-1000:12-12 | 25.143 | 1.440 | 0.190 | 0.155 | 25.539 |
| 20240129-0736 | DRKNAITGIVR | STING inactive 6MXE | A1-1000/B1-1000:11-11 | 32.973 | 1.193 | 0.189 | 0.144 | 26.788 |
| 20240129-0716 | SNPLPEKIVFP | STING inactive 6MXE | A1-1000/B1-1000:11-11 | 21.417 | 1.410 | 0.189 | 0.122 | 26.294 |
| 20240129-0708 | SSNLRKTVEIS | STING inactive 6MXE | A1-1000/B1-1000:11-11 | 20.943 | 1.327 | 0.189 | 0.162 | 24.875 |
| 20240129-0707 | SSLLRKSVEIS | STING inactive 6MXE | A1-1000/B1-1000:11-11 | 19.718 | 1.306 | 0.189 | 0.179 | 24.22 |
| 20240129-0583 | ASAAAQVHAIFR | STING inactive 6MXE | A1-1000/B1-1000:12-12 | 37.093 | 1.673 | 0.189 | 0.122 | 27.626 |
| 20240129-0577 | MPLHLSTGPVLP | STING inactive 6MXE | A1-1000/B1-1000:12-12 | 24.577 | 1.307 | 0.189 | 0.139 | 25.759 |
| 20240129-0530 | APMWRAPPIRLP | STING inactive 6MXE | A1-1000/B1-1000:12-12 | 52.115 | 1.426 | 0.189 | 0.147 | 26.374 |
| 20240127-0699 | GNPALIRRGLL | STING inactive 6MXE | A1-1000/B1-1000:11-11 | 19.289 | 1.308 | 0.189 | 0.135 | 25.444 |
| 20240127-0693 | GNPALIRRGLL | STING inactive 6MXE | A1-1000/B1-1000:11-11 | 19.289 | 1.296 | 0.189 | 0.135 | 25.444 |
| 20240127-0691 | GNPALIRRGLL | STING inactive 6MXE | A1-1000/B1-1000:11-11 | 19.289 | 1.301 | 0.189 | 0.135 | 25.444 |
| 20240127-0635 | SARVSQYETLS | STING inactive 6MXE | A1-1000/B1-1000:11-11 | 32.124 | 1.376 | 0.189 | 0.133 | 27.205 |
| 20240127-0593 | PKGSVLGIVRP | STING inactive 6MXE | A1-1000/B1-1000:11-11 | 28.076 | 1.250 | 0.189 | 0.152 | 26.292 |
| 20240127-0575 | SMKTSKPQQLWR | STING inactive 6MXE | A1-1000/B1-1000:12-12 | 27.867 | 1.467 | 0.189 | 0.161 | 25.449 |
| 20240127-0117 | SSFNRIALRSMSVFS | STING inactive 6MXE | A1-1000/B1-1000:15-15 | 21.106 | 1.498 | 0.189 | 0.13 | 26.537 |
| 20240127-0711 | SNARERFLKVS | STING inactive 6MXE | A1-1000/B1-1000:11-11 | 23.039 | 1.678 | 0.188 | 0.149 | 25.656 |
| 20240127-0666 | SSSFRKLQFRP | STING inactive 6MXE | A1-1000/B1-1000:11-11 | 24.078 | 1.481 | 0.188 | 0.197 | 24.688 |
| 20240127-0643 | MSSAAPLPRIP | STING inactive 6MXE | A1-1000/B1-1000:11-11 | 28.735 | 1.471 | 0.188 | 0.114 | 26.823 |
| 20240127-0574 | SMVTSKPKMLWR | STING inactive 6MXE | A1-1000/B1-1000:12-12 | 24.896 | 1.411 | 0.188 | 0.168 | 25.178 |
| 20240127-0562 | SMVTSRPVESWR | STING inactive 6MXE | A1-1000/B1-1000:12-12 | 23.634 | 1.499 | 0.188 | 0.133 | 25.966 |
| 20240127-0488 | MAVVEMNKSLLS | STING inactive 6MXE | A1-1000/B1-1000:12-12 | 20.107 | 1.343 | 0.188 | 0.152 | 25.726 |
| 20240129-0739 | PTIVESRLLES | STING inactive 6MXE | A1-1000/B1-1000:11-11 | 36.676 | 1.671 | 0.187 | 0.113 | 28.291 |
| 20240129-0695 | EVKVLKRSVMQ | STING inactive 6MXE | A1-1000/B1-1000:11-11 | 21.613 | 1.635 | 0.187 | 0.162 | 26.314 |
| 20240129-0544 | ASLTALVLSRPP | STING inactive 6MXE | A1-1000/B1-1000:12-12 | 50.573 | 1.606 | 0.187 | 0.149 | 26.368 |
| 20240127-0620 | FKELSNITIPV | STING inactive 6MXE | A1-1000/B1-1000:11-11 | 17.035 | 1.446 | 0.187 | 0.161 | 24.938 |
| 20240127-0568 | SLKTSKPKLLWR | STING inactive 6MXE | A1-1000/B1-1000:12-12 | 27.264 | 1.425 | 0.187 | 0.166 | 25.015 |
| 20240127-0558 | MIVYPEKIAYPR | STING inactive 6MXE | A1-1000/B1-1000:12-12 | 27.998 | 1.218 | 0.187 | 0.148 | 26.09 |
| 20240129-0701 | EPPQLLPPLLP | STING inactive 6MXE | A1-1000/B1-1000:11-11 | 47.2 | 1.069 | 0.186 | 0.142 | 26.01 |
| 20240129-0533 | GPKAAPLPIRLD | STING inactive 6MXE | A1-1000/B1-1000:12-12 | 20.117 | 1.214 | 0.186 | 0.143 | 25.684 |
| 20240129-0532 | MPMIRAPPIRLP | STING inactive 6MXE | A1-1000/B1-1000:12-12 | 50.976 | 1.448 | 0.186 | 0.109 | 26.742 |
| 20240129-0531 | MPEIRFPPIRTP | STING inactive 6MXE | A1-1000/B1-1000:12-12 | 27.875 | 1.482 | 0.186 | 0.122 | 26.237 |
| 20240129-0529 | MEKIEAPPVRLP | STING inactive 6MXE | A1-1000/B1-1000:12-12 | 53.195 | 1.467 | 0.186 | 0.108 | 26.8 |
| 20240127-0594 | PKGSVLGIYRP | STING inactive 6MXE | A1-1000/B1-1000:11-11 | 32.252 | 1.146 | 0.186 | 0.172 | 26.393 |
| 20240127-0572 | SMVTSKPKLLWQ | STING inactive 6MXE | A1-1000/B1-1000:12-12 | 23.701 | 1.439 | 0.186 | 0.124 | 26.538 |
| 20240127-0125 | SSLDRIRLKSMSVFR | STING inactive 6MXE | A1-1000/B1-1000:15-15 | 19.063 | 1.468 | 0.186 | 0.156 | 25.755 |
| 20240129-0696 | PVLTLSRSLML | STING inactive 6MXE | A1-1000/B1-1000:11-11 | 28.309 | 1.535 | 0.185 | 0.136 | 26.297 |
| 20240129-0672 | PVASRPLSMPR | STING inactive 6MXE | A1-1000/B1-1000:11-11 | 37.364 | 1.429 | 0.185 | 0.14 | 27.264 |
| 20240129-0650 | SLYSRSYTITH | STING inactive 6MXE | A1-1000/B1-1000:11-11 | 29.226 | 1.325 | 0.185 | 0.148 | 26.841 |
| 20240129-0634 | MSHSGPLPPTVP | STING inactive 6MXE | A1-1000/B1-1000:12-12 | 18.571 | 1.136 | 0.185 | 0.143 | 25.75 |
| 20240129-0633 | MSHSGPLPPTVP | STING inactive 6MXE | A1-1000/B1-1000:12-12 | 18.571 | 1.225 | 0.185 | 0.143 | 25.75 |
| 20240129-0541 | ESLRSLVVSQPP | STING inactive 6MXE | A1-1000/B1-1000:12-12 | 21.458 | 1.561 | 0.185 | 0.151 | 25.275 |
| 20240129-0536 | GPLASPLPIRLD | STING inactive 6MXE | A1-1000/B1-1000:12-12 | 26.579 | 1.248 | 0.185 | 0.12 | 26.418 |
| 20240127-0695 | GDPRLVRVGRL | STING inactive 6MXE | A1-1000/B1-1000:11-11 | 20.672 | 1.280 | 0.185 | 0.172 | 24.714 |
| 20240127-0655 | SSAAAPLPRLP | STING inactive 6MXE | A1-1000/B1-1000:11-11 | 27.713 | 1.471 | 0.185 | 0.115 | 26.742 |
| 20240127-0652 | ASSAMKLPLLP | STING inactive 6MXE | A1-1000/B1-1000:11-11 | 21.319 | 1.411 | 0.185 | 0.148 | 25.859 |
| 20240127-0629 | VARLSQYETLS | STING inactive 6MXE | A1-1000/B1-1000:11-11 | 31.166 | 1.314 | 0.185 | 0.137 | 27.114 |
| 20240127-0566 | SLKTSKPKQLWR | STING inactive 6MXE | A1-1000/B1-1000:12-12 | 27.543 | 1.431 | 0.185 | 0.195 | 24.287 |
| 20240127-0528 | ALPEIREIFHVS | STING inactive 6MXE | A1-1000/B1-1000:12-12 | 14.465 | 1.310 | 0.185 | 0.149 | 25.569 |
| 20240129-0534 | GAMASPLPIRLD | STING inactive 6MXE | A1-1000/B1-1000:12-12 | 20.981 | 1.282 | 0.184 | 0.143 | 25.599 |
| 20240127-0603 | PKPAVLGIYYP | STING inactive 6MXE | A1-1000/B1-1000:11-11 | 29.63 | 1.192 | 0.184 | 0.136 | 26.339 |
| 20240129-0767 | LVDASRIARAR | STING inactive 6MXE | A1-1000/B1-1000:11-11 | 26.846 | 1.065 | 0.183 | 0.134 | 26.233 |
| 20240129-0766 | LVDASRIARAR | STING inactive 6MXE | A1-1000/B1-1000:11-11 | 26.846 | 1.107 | 0.183 | 0.134 | 26.233 |
| 20240129-0651 | SLFLRSYTKIH | STING inactive 6MXE | A1-1000/B1-1000:11-11 | 20.487 | 1.435 | 0.183 | 0.166 | 25.415 |
| 20240129-0548 | GPIAGKIHSSAS | STING inactive 6MXE | A1-1000/B1-1000:12-12 | 21.034 | 1.641 | 0.183 | 0.167 | 24.867 |
| 20240129-0535 | GAMASPLPINLK | STING inactive 6MXE | A1-1000/B1-1000:12-12 | 21.071 | 1.340 | 0.183 | 0.138 | 25.852 |
| 20240127-0694 | GNPRLIREGRL | STING inactive 6MXE | A1-1000/B1-1000:11-11 | 31.325 | 1.314 | 0.183 | 0.159 | 26.186 |
| 20240129-0706 | SRNLRRTVEIS | STING inactive 6MXE | A1-1000/B1-1000:11-11 | 19.203 | 1.246 | 0.182 | 0.173 | 24.439 |
| 20240129-0646 | SSSSRHPLSVR | STING inactive 6MXE | A1-1000/B1-1000:11-11 | 24.452 | 1.378 | 0.182 | 0.131 | 26.362 |
| 20240129-0645 | SSSSRHPLSVR | STING inactive 6MXE | A1-1000/B1-1000:11-11 | 24.452 | 1.371 | 0.182 | 0.131 | 26.362 |
| 20240129-0538 | PKAPPVIEVLSL | STING inactive 6MXE | A1-1000/B1-1000:12-12 | 32.033 | 1.219 | 0.182 | 0.11 | 27.198 |
| 20240127-0646 | MSRAAPLPRLP | STING inactive 6MXE | A1-1000/B1-1000:11-11 | 27.284 | 1.468 | 0.182 | 0.117 | 26.559 |
| 20240129-0711 | SLPKVFRDIRL | STING inactive 6MXE | A1-1000/B1-1000:11-11 | 24.854 | 1.295 | 0.181 | 0.131 | 26.714 |
| 20240129-0550 | GPVLSVPKIIGK | STING inactive 6MXE | A1-1000/B1-1000:12-12 | 12.413 | 1.211 | 0.181 | 0.137 | 25.842 |
| 20240129-0540 | PKARPVMEVLSL | STING inactive 6MXE | A1-1000/B1-1000:12-12 | 25.364 | 1.290 | 0.181 | 0.132 | 26.375 |
| 20240127-0649 | SSRAAPLPRLP | STING inactive 6MXE | A1-1000/B1-1000:11-11 | 25.487 | 1.462 | 0.181 | 0.12 | 26.456 |
| 20240127-0644 | RSRAMKLPLLP | STING inactive 6MXE | A1-1000/B1-1000:11-11 | 20.91 | 1.451 | 0.181 | 0.135 | 26.032 |
| 20240127-0567 | SMKTLKPKLLWR | STING inactive 6MXE | A1-1000/B1-1000:12-12 | 26.061 | 1.404 | 0.181 | 0.157 | 25.66 |
| 20240127-0465 | SSSPLIRIHELR | STING inactive 6MXE | A1-1000/B1-1000:12-12 | 23.869 | 1.393 | 0.181 | 0.157 | 25.511 |
| 20240129-0668 | GLISERAEEAG | STING inactive 6MXE | A1-1000/B1-1000:11-11 | 30.36 | 1.508 | 0.180 | 0.164 | 26.26 |
| 20240129-0647 | SSSSRHPTSVR | STING inactive 6MXE | A1-1000/B1-1000:11-11 | 30.151 | 1.552 | 0.180 | 0.128 | 26.556 |
| 20240127-0623 | FKVLSNITIPV | STING inactive 6MXE | A1-1000/B1-1000:11-11 | 30.545 | 1.457 | 0.180 | 0.121 | 27.264 |
| 20240127-0569 | SMVTSKPKQLWR | STING inactive 6MXE | A1-1000/B1-1000:12-12 | 24.621 | 1.423 | 0.180 | 0.146 | 25.675 |
| 20240129-0715 | RNPLPRKIVFP | STING inactive 6MXE | A1-1000/B1-1000:11-11 | 23.466 | 1.445 | 0.179 | 0.13 | 26.311 |
| 20240129-0658 | SHMFRVELRNS | STING inactive 6MXE | A1-1000/B1-1000:11-11 | 23.783 | 1.474 | 0.179 | 0.174 | 24.933 |
| 20240129-0632 | SYAKRPAPSVKR | STING inactive 6MXE | A1-1000/B1-1000:12-12 | 26.424 | 1.312 | 0.179 | 0.124 | 26.542 |
| 20240127-0653 | RRRAAPLPRLP | STING inactive 6MXE | A1-1000/B1-1000:11-11 | 27.207 | 1.507 | 0.179 | 0.118 | 26.563 |
| 20240129-0705 | SSNLRRSVEIS | STING inactive 6MXE | A1-1000/B1-1000:11-11 | 19.958 | 1.325 | 0.178 | 0.171 | 24.383 |
| 20240127-0650 | SSSAMKLPLLP | STING inactive 6MXE | A1-1000/B1-1000:11-11 | 22.13 | 1.431 | 0.178 | 0.139 | 26.14 |
| 20240127-0621 | FKHLKNLTIPV | STING inactive 6MXE | A1-1000/B1-1000:11-11 | 24.103 | 1.392 | 0.178 | 0.122 | 26.522 |
| 20240127-0599 | PPGSVLGIFRP | STING inactive 6MXE | A1-1000/B1-1000:11-11 | 24.007 | 1.200 | 0.178 | 0.123 | 26.487 |
| 20240129-0734 | SRKNAISGIVR | STING inactive 6MXE | A1-1000/B1-1000:11-11 | 27.839 | 1.216 | 0.177 | 0.132 | 25.944 |
| 20240129-0731 | APVLRETRINL | STING inactive 6MXE | A1-1000/B1-1000:11-11 | 18.796 | 1.303 | 0.177 | 0.186 | 24.348 |
| 20240129-0722 | MEVAPIRVAQP | STING inactive 6MXE | A1-1000/B1-1000:11-11 | 16.904 | 1.280 | 0.177 | 0.124 | 25.946 |
| 20240129-0681 | GSVLSKKTSGK | STING inactive 6MXE | A1-1000/B1-1000:11-11 | 28.26 | 1.789 | 0.177 | 0.158 | 26.031 |
| 20240129-0659 | SERFVVELRLS | STING inactive 6MXE | A1-1000/B1-1000:11-11 | 23.199 | 1.537 | 0.177 | 0.17 | 24.556 |
| 20240127-0679 | SAPLRPLRPLG | STING inactive 6MXE | A1-1000/B1-1000:11-11 | 30.519 | 1.227 | 0.177 | 0.147 | 26.073 |
| 20240127-0673 | AAALKAIRPLG | STING inactive 6MXE | A1-1000/B1-1000:11-11 | 21.135 | 1.286 | 0.177 | 0.129 | 26.145 |
| 20240127-0651 | SSRAMPLPRLP | STING inactive 6MXE | A1-1000/B1-1000:11-11 | 26.491 | 1.402 | 0.177 | 0.118 | 26.613 |
| 20240127-0598 | PKPAVLGIVKP | STING inactive 6MXE | A1-1000/B1-1000:11-11 | 19.328 | 1.268 | 0.177 | 0.134 | 25.932 |
| 20240129-0710 | SLPKVFRSIRL | STING inactive 6MXE | A1-1000/B1-1000:11-11 | 22.834 | 1.265 | 0.176 | 0.186 | 24.69 |
| 20240129-0709 | SLPKVFRSIRL | STING inactive 6MXE | A1-1000/B1-1000:11-11 | 22.834 | 1.324 | 0.176 | 0.186 | 24.69 |
| 20240127-0654 | LSRAAPLPRLP | STING inactive 6MXE | A1-1000/B1-1000:11-11 | 27.047 | 1.477 | 0.176 | 0.126 | 26.346 |
| 20240129-0735 | SRSSAITGLVR | STING inactive 6MXE | A1-1000/B1-1000:11-11 | 33.817 | 1.333 | 0.175 | 0.147 | 26.912 |
| 20240129-0648 | SSSSRHPLAVR | STING inactive 6MXE | A1-1000/B1-1000:11-11 | 28.281 | 1.452 | 0.175 | 0.122 | 26.622 |
| 20240127-0687 | SAPLKAIRPLG | STING inactive 6MXE | A1-1000/B1-1000:11-11 | 30.464 | 1.263 | 0.175 | 0.136 | 26.396 |
| 20240127-0678 | SAPLKAIRPLG | STING inactive 6MXE | A1-1000/B1-1000:11-11 | 30.464 | 1.246 | 0.175 | 0.136 | 26.396 |
| 20240127-0113 | SSLARIRLRSMSVFR | STING inactive 6MXE | A1-1000/B1-1000:15-15 | 19.727 | 1.496 | 0.175 | 0.197 | 24.547 |
| 20240129-0721 | MQVAPIITAQP | STING inactive 6MXE | A1-1000/B1-1000:11-11 | 30.97 | 1.396 | 0.174 | 0.109 | 26.736 |
| 20240129-0542 | PQLRLLVVSRPP | STING inactive 6MXE | A1-1000/B1-1000:12-12 | 18.759 | 1.511 | 0.174 | 0.167 | 25.295 |
| 20240129-0729 | SVVLRPTAINL | STING inactive 6MXE | A1-1000/B1-1000:11-11 | 30.123 | 1.358 | 0.173 | 0.159 | 25.892 |
| 20240129-0723 | MEVSPIITFQP | STING inactive 6MXE | A1-1000/B1-1000:11-11 | 35.571 | 1.336 | 0.173 | 0.156 | 25.443 |
| 20240129-0687 | LLRSVEVLSAR | STING inactive 6MXE | A1-1000/B1-1000:11-11 | 17.788 | 1.255 | 0.173 | 0.185 | 24.59 |
| 20240129-0670 | PVAKRPLGLPR | STING inactive 6MXE | A1-1000/B1-1000:11-11 | 24.073 | 1.466 | 0.173 | 0.12 | 26.643 |
| 20240129-0669 | MVAKRVLSLPR | STING inactive 6MXE | A1-1000/B1-1000:11-11 | 38.889 | 1.433 | 0.173 | 0.131 | 26.932 |
| 20240129-0665 | GLIQEQADAYG | STING inactive 6MXE | A1-1000/B1-1000:11-11 | 24.951 | 1.445 | 0.173 | 0.129 | 26.158 |
| 20240127-0682 | SVPLRPLRPLG | STING inactive 6MXE | A1-1000/B1-1000:11-11 | 20.158 | 1.252 | 0.173 | 0.167 | 25.203 |
| 20240129-0547 | GPVAGKVVSSAS | STING inactive 6MXE | A1-1000/B1-1000:12-12 | 25.144 | 1.640 | 0.172 | 0.126 | 26.754 |
| 20240127-0685 | SAPLRALRPLG | STING inactive 6MXE | A1-1000/B1-1000:11-11 | 27.645 | 1.231 | 0.172 | 0.137 | 26.148 |
| 20240127-0681 | SAPLRALRPLG | STING inactive 6MXE | A1-1000/B1-1000:11-11 | 27.645 | 1.211 | 0.172 | 0.137 | 26.148 |
| 20240127-0677 | SAPLRALRPLG | STING inactive 6MXE | A1-1000/B1-1000:11-11 | 27.645 | 1.250 | 0.172 | 0.137 | 26.148 |
| 20240127-0675 | SAPLRALRPLG | STING inactive 6MXE | A1-1000/B1-1000:11-11 | 27.645 | 1.232 | 0.172 | 0.137 | 26.148 |
| 20240127-0674 | SAPLRALRPLG | STING inactive 6MXE | A1-1000/B1-1000:11-11 | 27.645 | 1.257 | 0.172 | 0.137 | 26.148 |
| 20240129-0732 | SMVLRETAINL | STING inactive 6MXE | A1-1000/B1-1000:11-11 | 26.07 | 1.267 | 0.171 | 0.13 | 26.096 |
| 20240127-0645 | MSSAMKLPRLP | STING inactive 6MXE | A1-1000/B1-1000:11-11 | 25.25 | 1.463 | 0.171 | 0.122 | 26.567 |
| 20240129-0762 | MASSIEVLLRE | STING inactive 6MXE | A1-1000/B1-1000:11-11 | 23.441 | 1.549 | 0.170 | 0.165 | 25.25 |
| 20240129-0684 | SSRLSRKTSRE | STING inactive 6MXE | A1-1000/B1-1000:11-11 | 26.64 | 1.808 | 0.170 | 0.152 | 25.93 |
| 20240129-0671 | PVASRVRSMPR | STING inactive 6MXE | A1-1000/B1-1000:11-11 | 35.14 | 1.448 | 0.170 | 0.125 | 26.488 |
| 20240129-0657 | MERFVVELRNS | STING inactive 6MXE | A1-1000/B1-1000:11-11 | 19.144 | 1.523 | 0.170 | 0.135 | 25.901 |
| 20240127-0684 | SVPLKPLRPLG | STING inactive 6MXE | A1-1000/B1-1000:11-11 | 30.669 | 1.244 | 0.170 | 0.138 | 26.415 |
| 20240127-0676 | SVPLKPLRPLG | STING inactive 6MXE | A1-1000/B1-1000:11-11 | 30.669 | 1.246 | 0.170 | 0.138 | 26.415 |
| 20240127-0616 | FKKLANITIPV | STING inactive 6MXE | A1-1000/B1-1000:11-11 | 21.038 | 1.442 | 0.170 | 0.133 | 26.122 |
| 20240129-0543 | EQLRLLVVSQPP | STING inactive 6MXE | A1-1000/B1-1000:12-12 | 25.541 | 1.440 | 0.169 | 0.144 | 25.648 |
| 20240129-0686 | MRRSVEVLSAR | STING inactive 6MXE | A1-1000/B1-1000:11-11 | 17.094 | 1.285 | 0.168 | 0.177 | 24.686 |
| 20240127-0612 | FVHMPNMVFPR | STING inactive 6MXE | A1-1000/B1-1000:11-11 | 19.165 | 1.496 | 0.168 | 0.145 | 25.572 |

| **Table S2 Hematological (CBC) and serum biochemical parameters of mice in each group at day 14 post-treatment** | | | | | | |
| --- | --- | --- | --- | --- | --- | --- |
| **Parameter** | **Unit** | **Control (PBS)** | **HeraDerm** | **Free SCP-1** | **Gel-SCP-1** | **Statistical result** |
| **Hematological Parameters (CBC)** |  |  |  |  |  |  |
| White blood cell count (WBC) | ×10⁹/L | 10.24 ± 1.35 | 9.87 ± 1.21 | 10.52 ± 1.48 | 9.95 ± 1.16 | ns |
| Red blood cell count (RBC) | ×10¹²/L | 8.62 ± 0.54 | 8.47 ± 0.49 | 8.55 ± 0.51 | 8.51 ± 0.46 | ns |
| Hemoglobin (HGB) | g/L | 138.6 ± 7.23 | 135.2 ± 6.85 | 137.4 ± 7.01 | 136.1 ± 6.58 | ns |
| Platelet count (PLT) | ×10⁹/L | 892.4 ± 105.6 | 875.3 ± 98.7 | 901.5 ± 112.3 | 884.6 ± 95.4 | ns |
| Neutrophil percentage (NEUT%) | % | 22.36 ± 3.15 | 21.84 ± 2.98 | 23.05 ± 3.27 | 22.01 ± 2.86 | ns |
| Lymphocyte percentage (LYMPH%) | % | 72.54 ± 3.42 | 73.12 ± 3.25 | 71.86 ± 3.51 | 72.89 ± 3.18 | ns |
| **Serum Biochemical Parameters** |  |  |  |  |  |  |
| Alanine aminotransferase (ALT) | U/L | 38.52 ± 5.34 | 37.86 ± 4.95 | 39.15 ± 5.62 | 38.04 ± 4.78 | ns |
| Aspartate aminotransferase (AST) | U/L | 92.45 ± 10.23 | 90.18 ± 9.56 | 93.72 ± 10.85 | 91.06 ± 9.24 | ns |
| Blood urea nitrogen (BUN) | mmol/L | 7.85 ± 0.86 | 7.62 ± 0.79 | 7.94 ± 0.91 | 7.71 ± 0.75 | ns |
| Serum creatinine (Scr) | μmol/L | 32.46 ± 4.12 | 31.85 ± 3.87 | 33.02 ± 4.35 | 32.17 ± 3.76 | ns |

Note: Data are presented as mean ± SD, n = 6 mice per group. ns indicates no statistically significant difference (*p* ≥ 0.05) among all groups, determined by one-way ANOVA followed by Tukey’s post-hoc test.
